# Supplementary material for: Calculation of the Isobaric Heat Capacities of the Liquid and Solid Phase of Organic Compounds at 298.15K by Means of the Group-Additivity Method
Source: Molecules. 2020 Mar 4;25(5):1147. doi: 10.3390/molecules25051147 (PMC7179130; doi:10.3390/molecules25051147)
Supplement: Supplementary file 1 [file molecules-25-01147-s001.zip › S03. Experimental vs. calculated Cp(liq,298) Data Table.doc]

| **Molecules sorted by calc values** |  |  |  |  |  |
| --- | --- | --- | --- | --- | --- |
| **Molecule name** | **Cp(liq,298) exp** | **Cp(liq,298) calc** | **Cp(liq,298) test** | **Deviation** | **Dev. in %** |
| Acetonitrile | 91.70 | 85.80 | 85.90 | 5.80 | 6.32 |
| Acetaldehyde | 89.05 | 92.00 | 92.30 | -3.25 | -3.65 |
| Methanol | 81.92 | 95.60 | 96.50 | -14.58 | -17.80 |
| Hydrazine | 92.89 | 97.30 |  | -4.41 | -4.75 |
| Formamide | 107.90 | 100.90 |  | 7.00 | 6.49 |
| Bromoethane | 105.80 | 101.00 | 100.70 | 5.10 | 4.82 |
| Nitromethane | 105.98 | 102.20 | 101.90 | 4.08 | 3.85 |
| 1,1,1-Trifluoroethane | 109.60 | 103.90 | 103.20 | 6.40 | 5.84 |
| Ethyl iodide | 115.10 | 104.80 | 103.90 | 11.20 | 9.73 |
| Formic acid | 99.04 | 107.20 | 108.60 | -9.56 | -9.65 |
| Cyanoethylene | 110.88 | 107.20 | 106.50 | 4.38 | 3.95 |
| Acetylchloride | 117.20 | 107.60 | 106.30 | 10.90 | 9.30 |
| Oxetane | 99.60 | 109.60 | 110.40 | -10.80 | -10.84 |
| trans-1,2-Dichloroethylene | 114.24 | 112.80 |  | 1.44 | 1.26 |
| cis-1,2-Dichloroethylene | 113.80 | 112.80 |  | 1.00 | 0.88 |
| Vinylidenechloride | 111.29 | 112.90 |  | -1.61 | -1.45 |
| Ethanethiol | 117.90 | 113.20 | 112.80 | 5.10 | 4.33 |
| Methyl formate | 119.10 | 113.50 | 115.70 | 3.40 | 2.85 |
| Dimethyl sulfide | 118.10 | 114.90 | 114.40 | 3.70 | 3.13 |
| Trifluoroacetonitrile | 118.00 | 115.70 | 114.50 | 3.50 | 2.97 |
| Furan | 114.56 | 115.70 | 117.20 | -2.64 | -2.30 |
| Propanenitrile | 119.30 | 115.90 | 115.40 | 3.90 | 3.27 |
| Thietane | 113.39 | 116.00 | 117.40 | -4.01 | -3.54 |
| cis-2-Butene | 127.00 | 119.60 | 119.30 | 7.70 | 6.06 |
| trans-2-Butene | 124.30 | 119.60 | 119.60 | 4.70 | 3.78 |
| 2-Oxetanone | 122.10 | 119.60 | 119.70 | 2.40 | 1.97 |
| Methyloxiran | 120.40 | 119.60 | 119.90 | 0.50 | 0.42 |
| 1,3-Dioxolane | 122.16 | 120.90 | 120.60 | 1.56 | 1.28 |
| Cyclopentadiene | 115.30 | 121.20 | 120.20 | -4.90 | -4.25 |
| 2-Butyne | 124.30 | 122.00 | 121.00 | 3.30 | 2.65 |
| Propanal | 134.70 | 122.10 | 120.80 | 13.90 | 10.32 |
| Allyl chloride | 125.10 | 122.10 | 122.20 | 2.90 | 2.32 |
| 1,1-Dichloroethane | 126.27 | 123.20 | 122.70 | 3.57 | 2.83 |
| Propanone | 124.70 | 124.30 | 124.50 | 0.20 | 0.16 |
| Ethanol | 115.90 | 125.20 | 125.60 | -9.70 | -8.37 |
| 1-Butene | 128.96 | 125.50 | 125.50 | 3.46 | 2.68 |
| 1-Chloro-1,1-difluoroethane | 130.50 | 126.70 | 124.30 | 6.20 | 4.75 |
| Pyrrole | 127.74 | 126.80 | 122.60 | 5.14 | 4.02 |
| Ethylenedichloride | 128.81 | 127.30 | 127.00 | 1.81 | 1.41 |
| 1-Bromo-2-chloroethane | 130.10 | 127.60 | 127.40 | 2.70 | 2.08 |
| 1,2-Dibromoethane | 134.72 | 127.90 | 125.00 | 9.72 | 7.21 |
| Cyclopentene | 122.38 | 127.90 | 128.10 | -5.72 | -4.67 |
| N-Methylformamide | 123.90 | 128.20 |  | -4.30 | -3.47 |
| Acetic acid | 123.10 | 128.70 | 129.10 | -6.00 | -4.87 |
| Tetrahydrofuran | 123.90 | 128.90 | 129.10 | -5.20 | -4.20 |
| Spiro[2.2]pentane | 134.52 | 129.30 | 128.30 | 6.22 | 4.62 |
| Cyclopropanecarbonitrile | 115.50 | 130.60 | 132.30 | -16.80 | -14.55 |
| Cyclopentane | 128.80 | 130.70 | 130.90 | -2.10 | -1.63 |
| 1-Chloropropane | 131.30 | 130.80 | 130.80 | 0.50 | 0.38 |
| 2-Bromopropane | 135.60 | 130.90 | 131.00 | 4.60 | 3.39 |
| 1-Bromopropane | 133.46 | 131.00 | 130.80 | 2.66 | 1.99 |
| Benzene | 136.80 | 132.00 | 131.60 | 5.20 | 3.80 |
| Methylhydrazine | 134.93 | 132.90 |  | 2.03 | 1.50 |
| 1,3-Dioxolan-2-one | 133.90 | 132.90 | 133.10 | 0.80 | 0.60 |
| Trichloroethylene | 128.90 | 133.70 |  | -4.80 | -3.72 |
| Perfluoroethane | 131.40 | 133.80 | 135.40 | -4.00 | -3.04 |
| Pyridine | 133.00 | 133.90 | 134.10 | -1.10 | -0.83 |
| Butane | 140.90 | 134.20 | 134.20 | 6.70 | 4.76 |
| Nitroethane | 134.22 | 134.20 | 134.10 | 0.12 | 0.09 |
| 1-Iodopropane | 136.20 | 134.80 | 134.80 | 1.40 | 1.03 |
| Methyl acetate | 140.56 | 135.00 | 135.20 | 5.36 | 3.81 |
| 2,3-Dihydrofuran | 122.10 | 135.10 | 136.60 | -14.50 | -11.88 |
| Thiolane | 140.16 | 137.10 | 136.80 | 3.36 | 2.40 |
| Propanoyl chloride | 147.30 | 137.60 | 136.40 | 10.90 | 7.40 |
| Crotonaldehyde | 148.60 | 137.60 | 138.90 | 9.70 | 6.53 |
| 2-Methylacrylonitrile | 126.30 | 137.70 | 137.60 | -11.30 | -8.95 |
| Cyclopropylamine | 147.11 | 138.30 | 135.20 | 11.91 | 8.10 |
| gamma-Butyrolactone | 141.40 | 138.90 | 138.90 | 2.50 | 1.77 |
| 1,1,1-Trichloroethane | 143.93 | 139.60 | 138.90 | 5.03 | 3.49 |
| trans-1,3-Pentadiene | 149.33 | 141.00 | 140.90 | 8.43 | 5.65 |
| Methylenecyclobutane | 133.60 | 141.00 | 141.50 | -7.90 | -5.91 |
| cis-1,3-Pentadiene | 146.57 | 141.00 | 140.90 | 5.67 | 3.87 |
| 2-Propanethiol | 145.35 | 142.50 | 141.20 | 4.15 | 2.86 |
| Methylnitrate | 156.90 | 142.80 |  | 14.10 | 8.99 |
| Ethyl methyl sulfide | 144.60 | 142.80 | 142.90 | 1.70 | 1.18 |
| 1-Cyanobicyclobutane | 131.80 | 143.00 | 145.60 | -13.80 | -10.47 |
| Ethyl formate | 144.30 | 143.10 | 143.10 | 1.20 | 0.83 |
| 1-Propanethiol | 144.56 | 143.30 | 143.10 | 1.46 | 1.01 |
| Isobutyronitrile | 156.10 | 143.90 | 144.00 | 12.10 | 7.75 |
| 2,3-Pentadiene | 152.34 | 144.80 |  | 7.54 | 4.95 |
| Butyronitrile | 134.20 | 145.90 | 145.40 | -11.20 | -8.35 |
| 2-Methylfuran | 143.72 | 146.10 | 148.00 | -4.28 | -2.98 |
| Epichlorohydrin | 131.60 | 146.30 | 147.30 | -15.70 | -11.93 |
| Allyl alcohol | 146.30 | 146.60 | 147.40 | -1.10 | -0.75 |
| Dimethyl disulfide | 146.11 | 146.60 | 147.00 | -0.89 | -0.61 |
| 1,4-Pentadiene | 146.82 | 146.90 | 146.90 | -0.08 | -0.05 |
| 1,3-Dioxane | 143.90 | 147.10 | 148.10 | -4.20 | -2.92 |
| Fluorobenzene | 146.29 | 147.10 | 146.80 | -0.51 | -0.35 |
| Isoprene | 151.08 | 147.30 | 147.20 | 3.88 | 2.57 |
| 1,3-Cyclohexadiene | 141.30 | 147.40 | 147.30 | -6.00 | -4.25 |
| Ethan-1,2-diol | 149.80 | 148.70 | 149.80 | 0.00 | 0.00 |
| Quadricyclane | 157.60 | 149.10 | 146.60 | 11.00 | 6.98 |
| trans-2-Pentene | 157.00 | 149.70 | 149.90 | 7.10 | 4.52 |
| Ethylepoxide | 147.00 | 149.70 | 149.80 | -2.80 | -1.90 |
| cis-2-Pentene | 151.80 | 149.70 | 149.70 | 2.10 | 1.38 |
| Pyrrolidine | 156.57 | 149.80 | 147.80 | 8.77 | 5.60 |
| 1,1,2-Trichloroethane | 150.90 | 149.80 | 150.40 | 0.50 | 0.33 |
| Acrylic acid | 144.20 | 150.10 | 149.30 | -5.10 | -3.54 |
| Trimethylethylene | 152.80 | 150.10 | 150.50 | 2.30 | 1.51 |
| 1-Methylpyrrole | 150.06 | 150.10 |  | -0.04 | -0.03 |
| 1,2-Pentadiene | 150.83 | 150.70 |  | 0.13 | 0.09 |
| N,N-Dimethylformamide | 148.36 | 151.00 |  | -2.64 | -1.78 |
| 3-Methyl-1,2-butadiene | 152.42 | 151.10 | 150.40 | 2.02 | 1.33 |
| Cyclobutanecarbonitrile | 146.00 | 151.20 | 153.10 | -7.10 | -4.86 |
| 1,4-Cyclohexadiene | 142.20 | 151.30 | 152.00 | -9.80 | -6.89 |
| Chlorobenzene | 153.78 | 151.30 | 151.90 | 1.88 | 1.22 |
| Isopropylamine | 164.00 | 151.60 | 150.10 | 13.90 | 8.48 |
| Butanal | 163.70 | 152.10 | 151.60 | 12.10 | 7.39 |
| 1-Methylimidazole | 147.20 | 153.20 |  | -6.00 | -4.08 |
| 1,1-Dichloropropane | 152.82 | 153.20 | 153.20 | -0.38 | -0.25 |
| 1-Propylamine | 161.90 | 153.30 | 153.20 | 8.70 | 5.37 |
| 1,4-Dioxane | 150.65 | 153.30 | 153.80 | -3.15 | -2.09 |
| Iodobenzene | 158.40 | 153.40 |  | 5.00 | 3.16 |
| 2-Pyrrolidone | 169.50 | 153.50 | 151.00 | 18.50 | 10.91 |
| 3-Methyl-1-butene | 156.10 | 153.60 | 154.60 | 1.50 | 0.96 |
| Cyclohexene | 152.90 | 154.10 | 154.50 | -1.60 | -1.05 |
| 2-Butanone | 158.40 | 154.40 | 154.30 | 4.10 | 2.59 |
| Tetrachloroethylene | 157.70 | 154.50 | 148.00 | 9.70 | 6.15 |
| 2-Methylthiophene | 149.83 | 154.50 | 157.00 | -7.17 | -4.79 |
| Tetrahydropyran | 149.60 | 155.10 | 154.80 | -5.20 | -3.48 |
| Chloroacetic acid | 179.90 | 155.30 | 154.60 | 25.30 | 14.06 |
| 1-Propanol | 146.88 | 155.30 | 155.80 | -8.92 | -6.07 |
| Dimethoxymethane | 163.60 | 155.40 | 153.50 | 10.10 | 6.17 |
| 1-Pentene | 155.30 | 155.60 | 156.30 | -1.00 | -0.64 |
| 3-Methylcyclopentene | 152.30 | 156.00 | 155.30 | -3.00 | -1.97 |
| Isopentene | 157.30 | 156.00 | 156.00 | 1.30 | 0.83 |
| Methyl acrylate | 158.80 | 156.40 | 155.80 | 3.00 | 1.89 |
| Cyclohexane | 158.10 | 156.80 | 156.70 | 1.40 | 0.89 |
| Chlorocyclopentane | 152.40 | 157.20 | 156.90 | -4.50 | -2.95 |
| 2,5-Norbornadiene | 161.20 | 157.30 | 155.80 | 5.40 | 3.35 |
| 1,2-Dichloropropane | 156.81 | 157.30 | 156.60 | 0.21 | 0.13 |
| 1,3-Dichloropropane | 157.00 | 157.40 | 157.10 | -0.10 | -0.06 |
| Chloral | 151.50 | 157.50 | 159.10 | -7.60 | -5.02 |
| Succinonitrile | 145.60 | 157.70 | 157.30 | -11.70 | -8.04 |
| 1,2-Dibromopropane | 172.80 | 157.80 | 152.70 | 20.10 | 11.63 |
| Methyltrichlorosilane | 163.10 | 157.80 |  | 5.30 | 3.25 |
| 1,3-Dibromopropane | 163.70 | 157.90 | 155.00 | 8.70 | 5.31 |
| 1-Methylcyclopentene | 153.10 | 158.40 | 158.80 | -5.70 | -3.72 |
| Methylbenzene | 158.70 | 158.60 | 158.20 | 0.50 | 0.32 |
| Propanoic acid | 158.60 | 158.70 | 158.00 | 0.60 | 0.38 |
| Cyclopentanone | 154.50 | 158.80 | 159.70 | -5.20 | -3.37 |
| Isobutyl chloride | 158.60 | 158.80 | 158.80 | -0.20 | -0.13 |
| Methylcyclopentane | 158.70 | 158.80 | 158.50 | 0.20 | 0.13 |
| Isobutyl bromide | 163.70 | 159.10 | 158.90 | 4.80 | 2.93 |
| 2-Picoline | 158.41 | 159.20 | 159.80 | -1.39 | -0.88 |
| 2-Methyltetrahydrofuran | 156.89 | 159.50 | 159.90 | -3.01 | -1.92 |
| Dimethyl carbonate | 157.90 | 159.50 | 159.40 | -1.50 | -0.95 |
| 3-Methylpyridine | 158.69 | 160.50 | 160.80 | -2.11 | -1.33 |
| 4-Methylpyridine | 158.99 | 160.50 | 160.80 | -1.81 | -1.14 |
| 3-Chloro-1,1,1-trifluoropropane | 171.08 | 160.60 | 159.60 | 11.48 | 6.71 |
| 2-Chlorobutane | 160.90 | 160.70 | 161.00 | -0.10 | -0.06 |
| 1-Chlorobutane | 159.64 | 160.80 | 160.90 | -1.26 | -0.79 |
| 1-Bromobutane | 162.20 | 161.10 | 161.10 | 1.10 | 0.68 |
| N-Methylpyrrolidine | 167.75 | 161.30 | 160.70 | 7.05 | 4.20 |
| 2-Cyanopropionaldehyde | 169.50 | 161.90 | 160.70 | 8.80 | 5.19 |
| 1,2-Difluorobenzene | 159.03 | 162.10 | 162.00 | -2.97 | -1.87 |
| 1,4-Difluorobenzene | 159.10 | 162.10 | 161.80 | -2.70 | -1.70 |
| 1,3-Difluorobenzene | 159.11 | 162.10 | 161.50 | -2.39 | -1.50 |
| Isopentane | 164.85 | 162.30 | 162.50 | 2.35 | 1.43 |
| Ethyl ether | 172.50 | 162.50 | 162.70 | 9.80 | 5.68 |
| Bromobenzene | 155.23 | 162.60 | 162.20 | -6.97 | -4.49 |
| Isobutyl iodide | 165.50 | 162.90 | 162.90 | 2.60 | 1.57 |
| Methylpropylether | 165.40 | 162.90 | 163.00 | 2.40 | 1.45 |
| Thiane | 163.30 | 163.20 | 163.50 | -0.20 | -0.12 |
| Propylenecarbonate | 167.40 | 163.50 | 163.40 | 4.00 | 2.39 |
| 1-Cyanobicyclo[2.1.0]pentane | 164.00 | 163.50 | 162.40 | 1.60 | 0.98 |
| Isopropyl methyl ether | 161.90 | 163.50 | 163.30 | -1.40 | -0.86 |
| 2-Methoxy-1-propene | 162.30 | 163.60 | 162.70 | -0.40 | -0.25 |
| Furfural | 162.80 | 164.00 | 166.30 | -3.50 | -2.15 |
| 1-Nitropropane | 160.40 | 164.20 | 164.30 | -3.90 | -2.43 |
| Pentane | 167.19 | 164.20 | 164.20 | 2.99 | 1.79 |
| Ethyl acetate | 169.06 | 164.60 | 164.80 | 4.26 | 2.52 |
| 1-Iodobutane | 164.50 | 164.90 | 166.40 | -1.90 | -1.16 |
| Tetrahydro-2H-pyran-2-one | 170.70 | 165.00 | 165.10 | 5.60 | 3.28 |
| Methyl propanoate | 172.74 | 165.10 | 165.00 | 7.74 | 4.48 |
| 3-Methylthiolane | 172.85 | 165.20 | 164.00 | 8.85 | 5.12 |
| 1,3-Diiodopropane | 169.20 | 165.50 | 163.70 | 5.50 | 3.25 |
| 2-Methylthiolane | 172.00 | 166.30 | 165.70 | 6.30 | 3.66 |
| 1,3,5-Cycloheptatriene | 162.76 | 166.80 | 167.80 | -5.04 | -3.10 |
| Dichlorodimethylsilane | 171.50 | 167.40 |  | 4.10 | 2.39 |
| Pivalonitrile | 179.37 | 167.60 | 167.20 | 12.17 | 6.78 |
| Butanoyl chloride | 170.70 | 167.70 | 167.20 | 3.50 | 2.05 |
| 1,2-Dimethylhydrazine | 171.04 | 168.60 |  | 2.44 | 1.43 |
| Cyclopentanethiol | 165.20 | 169.00 | 169.20 | -4.00 | -2.42 |
| t-Butylmercaptan | 174.90 | 169.50 | 168.20 | 6.70 | 3.83 |
| 1,1,1-Trichlorotrifluoroethane | 168.60 | 169.50 | 168.90 | -0.30 | -0.18 |
| Benzonitrile | 165.20 | 170.40 | 170.70 | -5.50 | -3.33 |
| Cyclopentanecarbonitrile | 167.40 | 170.50 | 170.00 | -2.60 | -1.55 |
| Diethyl sulphide | 171.42 | 170.60 | 170.50 | 0.92 | 0.54 |
| 1,3-Dichlorobenzene | 170.18 | 170.60 | 170.70 | -0.52 | -0.31 |
| 1,2-Dichlorobenzene | 170.90 | 170.60 | 170.80 | 0.10 | 0.06 |
| 2-Methyl-1-propanethiol | 171.88 | 171.40 | 170.80 | 1.08 | 0.63 |
| Isopropyl methyl sulfide | 172.38 | 172.00 | 172.10 | 0.28 | 0.16 |
| 1,1,2,2-Tetrachloroethane | 168.00 | 172.30 | 175.50 | -7.50 | -4.46 |
| 1,2-Diaminoethane | 172.60 | 172.30 | 173.20 | -0.60 | -0.35 |
| Ethylnitrate | 170.30 | 172.40 |  | -2.10 | -1.23 |
| 2-Butanethiol | 171.20 | 172.50 | 172.70 | -1.50 | -0.88 |
| 1,2-Dibromotetrafluoroethane | 173.80 | 172.80 | 159.40 | 14.40 | 8.29 |
| Methyl propyl sulfide | 171.63 | 172.80 | 172.40 | -0.77 | -0.45 |
| 1,3-Dioxepane | 167.00 | 173.20 | 175.50 | -8.50 | -5.09 |
| n-Propylformate | 171.50 | 173.20 | 175.70 | -4.20 | -2.45 |
| 1-Butanethiol | 172.30 | 173.30 | 173.90 | -1.60 | -0.93 |
| Norcarane | 187.90 | 173.60 | 174.40 | 13.50 | 7.18 |
| 2-Fluorotoluene | 171.20 | 173.70 | 173.90 | -2.70 | -1.58 |
| 4-Fluorotoluene | 173.70 | 173.70 | 173.60 | 0.10 | 0.06 |
| Pivalaldehyde | 185.58 | 173.80 | 173.70 | 11.88 | 6.40 |
| Morpholine | 164.80 | 174.10 | 174.70 | -9.90 | -6.01 |
| Colamine | 166.14 | 174.40 | 175.10 | -8.96 | -5.39 |
| Dimethylacetamide | 175.60 | 174.50 | 175.20 | 0.40 | 0.23 |
| 1,1-Difluoro-1,2,2-trichloroethane | 167.04 | 175.80 | 183.40 | -16.36 | -9.79 |
| Methylvinyldichlorosilane | 177.90 | 175.80 |  | 2.10 | 1.18 |
| Piperidine | 179.86 | 175.90 | 176.30 | 3.56 | 1.98 |
| N-Methyl-2-pyrrolidone | 166.26 | 176.30 | 179.20 | -12.94 | -7.78 |
| Phenylisocyanate | 186.20 | 176.40 | 166.70 | 19.50 | 10.47 |
| Benzaldehyde | 177.90 | 176.60 | 176.50 | 1.40 | 0.79 |
| 7-Oxabicyclo[4.1.0]heptane | 166.10 | 176.90 | 177.40 | -11.30 | -6.80 |
| 3,3-Dimethyl-1-butene | 188.30 | 177.30 | 176.70 | 11.60 | 6.16 |
| 2-Propanol | 154.43 | 177.70 | 178.90 | -24.47 | -15.85 |
| Dichloroacetic acid | 188.00 | 177.80 | 175.60 | 12.40 | 6.60 |
| 3-Methylpyrrolidine | 188.28 | 177.80 | 177.40 | 10.88 | 5.78 |
| 2-Chlorotoluene | 166.80 | 177.90 | 178.40 | -11.60 | -6.95 |
| Cyclopentylamine | 181.21 | 178.10 | 177.80 | 3.41 | 1.88 |
| 2,5-Dimethylthiophene | 178.32 | 178.40 |  | -0.08 | -0.04 |
| Propan-1,3-diol | 176.40 | 178.70 | 179.90 | -3.50 | -1.98 |
| Ethylacetamide | 179.90 | 179.20 | 178.50 | 1.40 | 0.78 |
| Dimethylmalononitrile | 179.50 | 179.40 | 179.00 | 0.50 | 0.28 |
| cis-2-Hexene | 178.36 | 179.80 | 179.80 | -1.44 | -0.81 |
| Styrene | 201.90 | 180.00 | 179.50 | 22.40 | 11.09 |
| Cycloheptene | 171.70 | 180.20 | 180.60 | -8.90 | -5.18 |
| Methacrylic acid | 161.10 | 180.50 | 182.10 | -21.00 | -13.04 |
| Tetramethylethene | 174.68 | 180.60 | 181.00 | -6.32 | -3.62 |
| 3-Methylenecyclobutanenitrile | 190.80 | 180.80 | 179.20 | 11.60 | 6.08 |
| 1-Ethylimidazole | 186.82 | 180.80 |  | 6.02 | 3.22 |
| 2-Methylpropylamine | 183.20 | 181.30 | 181.30 | 1.90 | 1.04 |
| Aniline | 178.70 | 181.60 | 180.70 | -2.00 | -1.12 |
| Bromo-2-chlorobenzene | 176.90 | 181.80 | 182.20 | -5.30 | -3.00 |
| N-Methylpropionamide | 179.00 | 181.80 | 183.50 | -4.50 | -2.51 |
| 3-Bromochlorobenzene | 181.90 | 181.80 | 182.20 | -0.30 | -0.16 |
| 4-Methylcyclohexene | 180.42 | 182.10 | 182.00 | -1.58 | -0.88 |
| n-Valeraldehyde | 174.39 | 182.20 | 183.80 | -9.41 | -5.40 |
| Nitrobenzene | 186.60 | 182.30 | 182.30 | 4.30 | 2.30 |
| Cyclooctatetraene | 185.18 | 182.30 | 183.70 | 1.48 | 0.80 |
| 1,1-Dimethylcyclopentane | 186.61 | 182.50 | 182.50 | 4.11 | 2.20 |
| 3-Methyl-2-butanone | 180.00 | 182.50 | 182.50 | -2.50 | -1.39 |
| Indole | 192.00 | 182.70 | 172.40 | 19.60 | 10.21 |
| 1-Cyanobicyclo[3.1.0]hexane | 170.30 | 182.90 | 182.70 | -12.40 | -7.28 |
| Cycloheptane | 180.61 | 183.00 | 183.00 | -2.39 | -1.32 |
| Diethylamine | 169.20 | 183.30 | 183.70 | -14.50 | -8.57 |
| 1-Butylamine | 179.20 | 183.30 | 182.80 | -3.60 | -2.01 |
| 2-Methyl-1-propanol | 181.05 | 183.40 | 183.60 | -2.55 | -1.41 |
| Benzo[b]furan | 178.70 | 183.50 | 184.50 | -5.80 | -3.25 |
| Bromocyclohexane | 182.10 | 183.60 | 184.10 | -2.00 | -1.10 |
| 1,2,3-Trichloropropane | 183.60 | 183.90 | 183.40 | 0.20 | 0.11 |
| Methyl cellosolve | 176.40 | 184.00 | 184.40 | -8.00 | -4.54 |
| 2-Bromoiodobenzene | 179.90 | 184.00 |  | -4.10 | -2.28 |
| 3-Bromoiodobenzene | 183.10 | 184.00 |  | -0.90 | -0.49 |
| Ethynylbenzene | 180.10 | 184.30 | 186.10 | -6.00 | -3.33 |
| Diethylketone | 190.90 | 184.50 | 184.50 | 6.40 | 3.35 |
| Ethylidenecyclopentane | 181.20 | 184.50 | 185.00 | -3.80 | -2.10 |
| 2,6-Lutidine | 185.17 | 184.50 | 184.00 | 1.17 | 0.63 |
| Methyl-n-propylketone | 184.10 | 184.50 | 184.60 | -0.50 | -0.27 |
| Ethyltrichlorosilane | 192.60 | 184.60 |  | 8.00 | 4.15 |
| 1,2,3-Tribromopropane | 166.50 | 184.70 | 192.80 | -26.30 | -15.80 |
| Perfluoropropane | 181.60 | 184.70 | 185.90 | -4.30 | -2.37 |
| Cyclohexanone | 182.20 | 184.90 | 184.80 | -2.60 | -1.43 |
| Methylcyclohexane | 184.96 | 184.90 | 184.90 | 0.06 | 0.03 |
| (Chloromethyl)benzene | 182.40 | 185.20 | 185.30 | -2.90 | -1.59 |
| 1,2-Dimethylbenzene | 187.65 | 185.20 | 185.10 | 2.55 | 1.36 |
| 1,3-Dimethylbenzene | 183.18 | 185.20 | 185.50 | -2.32 | -1.27 |
| 1,4-Dimethylbenzene | 183.65 | 185.20 | 185.20 | -1.55 | -0.84 |
| 1-Butanol | 177.16 | 185.40 | 185.80 | -8.64 | -4.88 |
| 1-Hexene | 183.30 | 185.60 | 185.60 | -2.30 | -1.25 |
| N-Methylmorpholine | 199.26 | 185.70 | 185.30 | 13.96 | 7.01 |
| 2,3-Lutidine | 189.55 | 185.80 | 185.40 | 4.15 | 2.19 |
| 2,4-Lutidine | 184.76 | 185.80 | 186.40 | -1.64 | -0.89 |
| 2,5-Lutidine | 184.70 | 185.80 | 186.10 | -1.40 | -0.76 |
| Neohexane | 189.67 | 186.00 | 186.00 | 3.67 | 1.93 |
| Allyl acetate | 184.10 | 186.00 | 186.30 | -2.20 | -1.20 |
| Methylenecyclohexane | 177.40 | 186.50 | 186.50 | -9.10 | -5.13 |
| 3-Chloro-1,1,1,3,3-pentafluoropropane | 196.60 | 186.60 | 182.30 | 14.30 | 7.27 |
| 2-Methylpropanoic acid | 181.70 | 186.80 | 187.60 | -5.90 | -3.25 |
| Indene | 186.94 | 186.80 | 186.70 | 0.24 | 0.13 |
| cis-1,2-Dimethylcyclopentane | 190.80 | 186.90 | 186.90 | 3.90 | 2.04 |
| trans-1,2-Dimethylcyclopentane | 188.70 | 186.90 | 186.90 | 1.80 | 0.95 |
| Methyl methacrylate | 188.70 | 186.90 | 187.00 | 1.70 | 0.90 |
| trans-1,3-Dimethylcyclopentane | 188.70 | 186.90 | 187.40 | 1.30 | 0.69 |
| 3,4-Lutidine | 196.00 | 187.10 | 185.10 | 10.90 | 5.56 |
| 3,5-Lutidine | 184.55 | 187.10 | 187.40 | -2.85 | -1.54 |
| cis-Bicyclo[3.3.0]octane | 213.40 | 187.30 | 186.90 | 26.50 | 12.42 |
| 1,2-Dichlorobutane | 195.60 | 187.30 | 186.70 | 8.90 | 4.55 |
| trans-Bicyclo[3.3.0]octane | 180.30 | 187.30 | 187.20 | -6.90 | -3.83 |
| 1,4-Dichlorobutane | 184.00 | 187.50 | 188.20 | -4.20 | -2.28 |
| N-Methylpiperidine | 184.90 | 187.50 | 187.30 | -2.40 | -1.30 |
| Pentanedinitrile | 183.30 | 187.70 | 188.20 | -4.90 | -2.67 |
| 2-Nitropropane | 170.30 | 187.90 |  | -17.60 | -10.33 |
| 1,4-Dibromobutane | 191.20 | 188.00 | 185.10 | 6.10 | 3.19 |
| t-Butylamine | 191.71 | 188.10 | 187.60 | 4.11 | 2.14 |
| 1-Ethylcyclopentene | 188.30 | 188.40 | 188.80 | -0.50 | -0.27 |
| Benzotrifluoride | 188.45 | 188.50 | 188.30 | 0.15 | 0.08 |
| Pentachloroethane | 196.20 | 188.70 | 186.60 | 9.60 | 4.89 |
| Ethylbenzene | 185.78 | 188.70 | 188.50 | -2.72 | -1.46 |
| Butanoic acid | 177.70 | 188.80 | 190.40 | -12.70 | -7.15 |
| Ethylcyclopentane | 187.40 | 188.80 | 188.70 | -1.30 | -0.69 |
| Isopentyl chloride | 179.50 | 188.90 | 188.60 | -9.10 | -5.07 |
| endo-2-Methylnorbornane | 184.30 | 189.20 | 188.70 | -4.40 | -2.39 |
| exo-2-Methylnorbornane | 185.80 | 189.20 | 188.70 | -2.90 | -1.56 |
| Isopentyl bromide | 187.00 | 189.20 | 188.00 | -1.00 | -0.53 |
| 1,2,4-Trichlorobenzene | 194.90 | 189.80 | 187.50 | 7.40 | 3.80 |
| 2,5-Dimethyltetrahydrofuran | 188.13 | 190.20 | 191.60 | -3.47 | -1.84 |
| 2,3-Dimethylbutane | 188.80 | 190.30 | 190.50 | -1.70 | -0.90 |
| MTBE | 187.50 | 190.50 | 189.80 | -2.30 | -1.23 |
| 1-Chloropentane | 188.12 | 190.90 | 191.00 | -2.88 | -1.53 |
| 1-Bromopentane | 191.33 | 191.10 | 190.90 | 0.43 | 0.22 |
| Caprolactone | 196.80 | 191.20 | 191.70 | 5.10 | 2.59 |
| 1,3-Dihydroisobenzofuran | 188.70 | 191.70 | 191.70 | -3.00 | -1.59 |
| Glyme | 191.14 | 191.70 | 191.30 | -0.16 | -0.08 |
| Benzoyl chloride | 187.00 | 192.10 | 199.80 | -12.80 | -6.84 |
| Phenol | 200.00 | 192.20 | 192.00 | 8.00 | 4.00 |
| 1,2,3,4-Tetrafluorobenzene | 189.90 | 192.20 | 192.60 | -2.70 | -1.42 |
| 1,2,3,5-Tetrafluorobenzene | 190.30 | 192.20 | 192.50 | -2.20 | -1.16 |
| Methyl bicyclobutane-1-carboxylate | 192.90 | 192.20 | 191.60 | 1.30 | 0.67 |
| 1,2,4,5-Tetrafluorobenzene | 192.20 | 192.20 | 192.60 | -0.40 | -0.21 |
| 2-Methylpentane | 193.96 | 192.30 | 193.00 | 0.96 | 0.49 |
| 3-Methylpentane | 190.83 | 192.30 | 191.50 | -0.67 | -0.35 |
| Ethyl-n-propylether | 197.20 | 192.50 | 192.40 | 4.80 | 2.43 |
| Isopentyliodide | 178.70 | 193.00 | 194.20 | -15.50 | -8.67 |
| n-Butyl methyl ether | 192.48 | 193.00 | 192.70 | -0.22 | -0.11 |
| 1,2-Dibromobenzene | 196.80 | 193.10 | 191.80 | 5.00 | 2.54 |
| 1,3-Dibromobenzene | 192.00 | 193.10 | 193.60 | -1.60 | -0.83 |
| Indane | 190.25 | 193.50 | 193.50 | -3.25 | -1.71 |
| 1,1-Dimethylsilacyclobutane | 197.53 | 193.80 | 192.70 | 4.83 | 2.45 |
| Hexane | 197.66 | 194.30 | 194.30 | 3.36 | 1.70 |
| Perfluorocyclobutane | 209.60 | 194.60 | 191.40 | 18.20 | 8.68 |
| Ethyl propanoate | 204.06 | 194.70 | 194.90 | 9.16 | 4.49 |
| n-Propyl acetate | 194.10 | 194.70 | 194.80 | -0.70 | -0.36 |
| Tetramethylsilane | 204.10 | 194.90 | 188.10 | 16.00 | 7.84 |
| Diethylmethylamine | 200.00 | 194.90 | 194.20 | 5.80 | 2.90 |
| 1-Iodopentane | 193.60 | 194.90 | 195.00 | -1.40 | -0.72 |
| Butyric acid methylester | 198.16 | 195.10 | 194.70 | 3.46 | 1.75 |
| Naphthalene | 196.06 | 195.10 | 195.00 | 1.06 | 0.54 |
| Cyclohexanethiol | 192.63 | 195.20 | 195.60 | -2.97 | -1.54 |
| Isopropylacetate | 196.60 | 195.20 | 195.80 | 0.80 | 0.41 |
| 1-Chloro-3-isocyanatobenzene | 187.00 | 195.70 | 204.80 | -17.80 | -9.52 |
| 1,1,1,3-Tetrachloropropane | 196.40 | 196.30 | 194.70 | 1.70 | 0.87 |
| Cyclohexanecarbonitrile | 177.80 | 196.70 | 197.40 | -19.60 | -11.02 |
| 4-Fluoroaniline | 195.20 | 196.70 | 196.40 | -1.20 | -0.61 |
| Isoquinoline | 196.16 | 197.00 | 195.10 | 1.06 | 0.54 |
| Furfuranol | 204.01 | 197.30 | 195.00 | 9.01 | 4.42 |
| Valeryl chloride | 187.90 | 197.70 | 205.20 | -17.30 | -9.21 |
| Benzyl mercaptan | 202.81 | 197.80 | 198.10 | 4.71 | 2.32 |
| Anisole | 199.00 | 198.50 | 200.40 | -1.40 | -0.70 |
| Cyclopentyl methyl sulfide | 193.18 | 198.60 | 199.00 | -5.82 | -3.01 |
| Quinoline | 199.20 | 198.90 |  | 0.30 | 0.15 |
| Methyl t-butyl sulfide | 199.95 | 199.00 | 198.70 | 1.25 | 0.63 |
| t-Amyl mercaptan | 198.15 | 199.50 | 200.10 | -1.95 | -0.98 |
| 1,1,1-Trichloro-3,3,3-trifluoropropane | 199.91 | 199.50 | 200.50 | -0.59 | -0.30 |
| 4-Methylpent-3-en-2-one | 212.50 | 200.40 | 199.50 | 13.00 | 6.12 |
| Methyl cyclobutanecarboxylate | 190.80 | 200.40 | 200.60 | -9.80 | -5.14 |
| 3-Methyl-2-butanethiol | 198.95 | 200.60 | 201.00 | -2.05 | -1.03 |
| Propylenediamine | 205.64 | 200.70 | 198.30 | 7.34 | 3.57 |
| Ethyl propyl sulfide | 198.41 | 200.70 | 200.50 | -2.09 | -1.05 |
| 3-Chloroaniline | 198.70 | 200.90 | 201.70 | -3.00 | -1.51 |
| 2-Chloroaniline | 199.00 | 200.90 | 200.90 | -1.90 | -0.95 |
| Isobutyl formate | 214.20 | 201.30 | 199.10 | 15.10 | 7.05 |
| 3-Methyl-1-butanethiol | 200.33 | 201.40 | 202.00 | -1.67 | -0.83 |
| 2-Methyl-1-butanethiol | 200.70 | 201.40 | 201.50 | -0.80 | -0.40 |
| 2-Chloronitrobenzene | 212.30 | 201.60 | 201.00 | 11.30 | 5.32 |
| Hexamethylenimine | 205.00 | 202.00 | 201.80 | 3.20 | 1.56 |
| Diethyl disulfide | 204.01 | 202.30 | 201.50 | 2.51 | 1.23 |
| 1,3-Diaminopropane | 203.80 | 202.40 | 203.70 | 0.10 | 0.05 |
| 2,3-Dihydrobenzofuran | 188.64 | 202.90 | 203.30 | -14.66 | -7.77 |
| n-Butylmethylsulfide | 200.92 | 202.90 | 203.00 | -2.08 | -1.04 |
| Isopropylnitrate | 191.10 | 203.10 |  | -12.00 | -6.28 |
| Trimethylvinylsilane | 198.00 | 203.20 | 204.90 | -6.90 | -3.48 |
| n-Butylformate | 200.25 | 203.30 | 205.70 | -5.45 | -2.72 |
| 1-Pentanethiol | 200.40 | 203.40 | 203.80 | -3.40 | -1.70 |
| 1,5-Cyclooctadiene | 208.10 | 203.60 | 204.00 | 4.10 | 1.97 |
| 4-Methylpiperidine | 209.00 | 204.00 | 202.30 | 6.70 | 3.21 |
| Cyclopentanol | 185.40 | 204.30 | 205.60 | -20.20 | -10.90 |
| 2-Methylpiperidine | 205.00 | 204.30 | 203.90 | 1.10 | 0.54 |
| N,N-Dimethylpropionamide | 209.00 | 204.60 | 202.70 | 6.30 | 3.01 |
| 2-Acetylthiophene | 210.70 | 204.70 | 201.60 | 9.10 | 4.32 |
| Pinacolone | 206.90 | 206.20 | 206.00 | 0.90 | 0.43 |
| cis- Cyclooctene | 207.80 | 206.30 | 205.70 | 2.10 | 1.01 |
| Ethyl cyanoacetate | 220.22 | 206.40 | 205.50 | 14.72 | 6.68 |
| Pentafluorobenzene | 210.79 | 207.30 | 206.70 | 4.09 | 1.94 |
| N-(1-Methylethyl)ethanamide | 211.00 | 207.60 | 209.60 | 1.40 | 0.66 |
| 2-Butanol | 196.67 | 207.70 | 208.70 | -12.03 | -6.12 |
| Benzylamine | 207.18 | 207.70 | 208.10 | -0.92 | -0.44 |
| m-Toluidine | 216.70 | 208.30 | 207.10 | 9.60 | 4.43 |
| o-Toluidine | 209.60 | 208.30 | 207.20 | 2.40 | 1.15 |
| 1,1-Dimethylcyclohexane | 209.24 | 208.60 | 208.90 | 0.34 | 0.16 |
| Tetramethylene glycol | 204.20 | 208.80 | 207.80 | -3.60 | -1.76 |
| 2-Nitrotoluene | 202.10 | 208.90 | 209.50 | -7.40 | -3.66 |
| 3-Nitrotoluene | 202.50 | 208.90 | 209.20 | -6.70 | -3.31 |
| Acetophenone | 205.50 | 208.90 | 209.30 | -3.80 | -1.85 |
| N-Methylaniline | 207.10 | 208.90 | 209.60 | -2.50 | -1.21 |
| Cyclooctane | 215.53 | 209.10 | 209.50 | 6.03 | 2.80 |
| 3-Dimethylaminopropanenitrile | 212.50 | 209.20 | 209.50 | 3.00 | 1.41 |
| Propylacetamide | 207.94 | 209.30 | 209.10 | -1.16 | -0.56 |
| Ethyl cyclopropanecarboxylate | 213.00 | 209.40 | 208.60 | 4.40 | 2.07 |
| Benzyl alcohol | 215.90 | 209.80 | 209.80 | 6.10 | 2.83 |
| N,2-Dimethylpropanamide | 209.20 | 209.80 | 211.90 | -2.70 | -1.29 |
| Acetylacetone (enol form) | 208.40 | 210.20 | 233.80 | -25.40 | -12.19 |
| Allylcyclopentane | 202.90 | 210.20 | 210.80 | -7.90 | -3.89 |
| alpha-Methylstyrene | 202.10 | 210.50 | 209.30 | -7.20 | -3.56 |
| Tetrahydrofurfuryl alcohol | 190.00 | 210.70 | 212.40 | -22.40 | -11.79 |
| Ethylidenecyclohexane | 203.80 | 210.70 | 211.70 | -7.90 | -3.88 |
| Suberone | 211.80 | 211.00 | 210.20 | 1.60 | 0.76 |
| 2-Chlorophenol | 217.50 | 211.50 | 210.20 | 7.30 | 3.36 |
| 3-Chlorophenol | 211.00 | 211.50 | 211.50 | -0.50 | -0.24 |
| N-Methylbutylamide | 207.00 | 211.80 | 212.70 | -5.70 | -2.75 |
| Hemimellitene | 216.44 | 211.80 | 212.20 | 4.24 | 1.96 |
| 1,3,5-Trimethylbenzene | 207.85 | 211.80 | 211.50 | -3.65 | -1.76 |
| 1,2,4-Trimethylbenzene | 213.11 | 211.80 | 212.00 | 1.11 | 0.52 |
| 1-Amino-3-methoxypropane | 225.52 | 212.10 | 211.60 | 13.92 | 6.17 |
| Caproic aldehyde | 210.40 | 212.20 | 212.40 | -2.00 | -0.95 |
| Ethylisopropylketone | 187.90 | 212.50 | 212.80 | -24.90 | -13.25 |
| Methyl isobutyl ketone | 211.90 | 212.50 | 212.80 | -0.90 | -0.42 |
| 2-Isopropylthiophene | 211.38 | 212.60 |  | -1.22 | -0.58 |
| cis-1,3-Dimethylcyclohexane | 209.37 | 213.00 | 213.80 | -4.43 | -2.12 |
| trans-1,2-Dimethylcyclohexane | 209.41 | 213.00 | 212.80 | -3.39 | -1.62 |
| trans-1,4-Dimethylcyclohexane | 210.25 | 213.00 | 212.60 | -2.35 | -1.12 |
| cis-1,2-Dimethylcyclohexane | 210.87 | 213.00 | 212.80 | -1.93 | -0.92 |
| cis-1,4-Dimethylcyclohexane | 212.09 | 213.00 | 213.50 | -1.41 | -0.66 |
| trans-1,3-Dimethylcyclohexane | 212.84 | 213.00 | 213.80 | -0.96 | -0.45 |
| N-Ethylmorpholine | 207.32 | 213.20 | 213.80 | -6.48 | -3.13 |
| Amylamine | 218.00 | 213.40 | 212.70 | 5.30 | 2.43 |
| trans-Hydrindan | 209.70 | 213.40 | 214.20 | -4.50 | -2.15 |
| Isopentyl alcohol | 209.60 | 213.40 | 213.00 | -3.40 | -1.62 |
| cis-Hydrindan | 214.18 | 213.40 | 214.20 | -0.02 | -0.01 |
| 2-Ethoxyethanol | 210.30 | 213.60 | 213.30 | -3.00 | -1.43 |
| Ethyl dichloroacetate | 209.60 | 213.70 | 214.30 | -4.70 | -2.24 |
| Triptane | 213.51 | 214.00 | 213.80 | -0.29 | -0.14 |
| 1-Chloronaphthalene | 212.60 | 214.40 | 214.80 | -2.20 | -1.03 |
| 3-Hexanone | 216.90 | 214.50 | 214.50 | 2.40 | 1.11 |
| Butylmethylketone | 213.40 | 214.50 | 214.00 | -0.60 | -0.28 |
| 3,3-Bis(chloromethyl)oxetane | 218.80 | 214.60 | 214.30 | 4.50 | 2.06 |
| 1-Ethylpiperidine | 232.06 | 215.00 | 213.30 | 18.76 | 8.08 |
| Ethylcyclohexane | 211.79 | 215.00 | 214.30 | -2.51 | -1.19 |
| 2-Hydroxybenzaldehyde | 222.00 | 215.30 | 234.60 | -12.60 | -5.68 |
| 1-Pentanol | 208.14 | 215.40 | 215.70 | -7.56 | -3.63 |
| Bis(2-chloroethyl) ether | 220.90 | 215.70 | 215.20 | 5.70 | 2.58 |
| 1-Heptene | 211.79 | 215.70 | 215.30 | -3.51 | -1.66 |
| Cellosolve acetate | 203.00 | 215.80 | 215.90 | -12.90 | -6.35 |
| 2,2-Dimethylpentane | 221.12 | 216.00 | 215.90 | 5.22 | 2.36 |
| 3,3-Dimethylpentane | 214.80 | 216.00 | 216.80 | -2.00 | -0.93 |
| Cumene | 215.40 | 216.70 | 216.90 | -1.50 | -0.70 |
| Methyl pivalate | 223.00 | 216.80 | 216.50 | 6.50 | 2.91 |
| 3-Methylbutanoic acid | 197.10 | 216.90 | 217.60 | -20.50 | -10.40 |
| 1,5-Dichloropentane | 213.40 | 217.50 | 218.00 | -4.60 | -2.16 |
| Tetrahydrodicyclopentadiene | 236.50 | 217.80 | 217.30 | 19.20 | 8.12 |
| 3,4-Dihydro-1H-2-benzopyran | 217.77 | 217.90 | 217.50 | 0.27 | 0.12 |
| 1,5-Dibromopentane | 220.84 | 218.00 | 217.50 | 3.34 | 1.51 |
| 1,4-Dimethylpiperazine | 210.11 | 218.10 | 220.50 | -10.39 | -4.95 |
| Isocumene | 214.72 | 218.70 | 218.60 | -3.88 | -1.81 |
| Diethylcarbonate | 218.55 | 218.70 | 218.30 | 0.25 | 0.11 |
| o-Cresol | 229.75 | 218.80 | 218.90 | 10.85 | 4.72 |
| m-Cresol | 224.93 | 218.80 | 219.90 | 5.03 | 2.24 |
| p-Cresol | 221.03 | 218.80 | 219.10 | 1.93 | 0.87 |
| Pentanoic acid | 210.00 | 218.90 | 218.10 | -8.10 | -3.86 |
| Propylcyclopentane | 216.27 | 218.90 | 218.90 | -2.63 | -1.22 |
| Methyl benzoate | 221.30 | 219.50 | 218.90 | 2.40 | 1.08 |
| 1,2,3,4-Tetrahydronaphthalene | 217.44 | 219.60 | 219.90 | -2.46 | -1.13 |
| Ethyl t-butyl ether | 218.00 | 220.10 | 220.50 | -2.50 | -1.15 |
| 5,6,7,8-Tetrahydroquinoline | 217.41 | 220.20 | 220.70 | -3.29 | -1.51 |
| t-Butyl vinyl ether | 217.60 | 220.40 | 222.00 | -4.40 | -2.02 |
| 2,4-Dimethylpentane | 224.22 | 220.40 | 220.50 | 3.72 | 1.66 |
| 2,3-Dimethylpentane | 218.30 | 220.40 | 220.00 | -1.70 | -0.78 |
| Methyl t-pentyl ether | 222.00 | 220.60 | 221.10 | 0.90 | 0.41 |
| Isobutyl vinyl ether | 231.80 | 220.90 | 217.70 | 14.10 | 6.08 |
| Dichlorodiethylsilane | 214.70 | 220.90 |  | -6.20 | -2.89 |
| 1-Chlorohexane | 216.73 | 220.90 | 221.20 | -4.47 | -2.06 |
| Hexyl bromide | 219.70 | 221.20 | 221.00 | -1.30 | -0.59 |
| Ethyl 2-chloropropionate | 220.50 | 221.20 | 220.30 | 0.20 | 0.09 |
| 2,5-Dioxaheptane | 224.70 | 221.30 | 221.40 | 3.30 | 1.47 |
| 2-Methylnaphthalene | 228.00 | 221.70 | 221.50 | 6.50 | 2.85 |
| 1-Methylnaphthalene | 224.39 | 221.70 | 219.70 | 4.69 | 2.09 |
| Cinnamaldehyde | 232.60 | 222.10 | 222.20 | 10.40 | 4.47 |
| t-Butylacetate | 231.00 | 222.20 | 221.00 | 10.00 | 4.33 |
| 3-Ethylpentane | 219.58 | 222.40 | 222.40 | -2.82 | -1.28 |
| Hexafluorobenzene | 221.60 | 222.40 | 223.90 | -2.30 | -1.04 |
| Triethylamine | 219.90 | 222.40 | 221.90 | -2.00 | -0.91 |
| 2-Methylhexane | 222.92 | 222.40 | 222.30 | 0.62 | 0.28 |
| Dipropyl ether | 221.45 | 222.60 | 223.00 | -1.55 | -0.70 |
| Isobutyl acetate | 233.80 | 222.70 | 222.20 | 11.60 | 4.96 |
| Butyl vinyl ether | 231.80 | 222.90 | 219.80 | 12.00 | 5.18 |
| Diisopropyl ether | 216.74 | 223.70 | 224.80 | -8.06 | -3.72 |
| Trichlorotoluene | 218.67 | 224.10 | 224.80 | -6.13 | -2.80 |
| 2-Methylquinoline | 224.72 | 224.20 |  | 0.52 | 0.23 |
| Heptane | 225.33 | 224.40 | 224.40 | 0.93 | 0.41 |
| Ethyl butanoate | 220.10 | 224.70 | 225.00 | -4.90 | -2.23 |
| Propyl propanoate | 226.65 | 224.70 | 224.40 | 2.25 | 0.99 |
| n-Butyl acetate | 225.10 | 224.70 | 224.80 | 0.30 | 0.13 |
| 1-Iodohexane | 222.50 | 225.00 | 224.10 | -1.60 | -0.72 |
| Methyl valerate | 226.11 | 225.20 | 225.40 | 0.71 | 0.31 |
| 8-Methylquinoline | 224.50 | 225.50 |  | -1.00 | -0.45 |
| cis-Bicyclo[6.1.0]nonane | 235.10 | 225.80 | 224.60 | 10.50 | 4.47 |
| Dimethyl malonate | 210.10 | 226.00 | 226.40 | -16.30 | -7.76 |
| 2-Methyl-2-propanol | 218.60 | 226.50 | 227.50 | -8.90 | -4.07 |
| Cyclohexyl formate | 219.70 | 226.50 | 227.60 | -7.90 | -3.60 |
| Chloropentafluorobenzene | 223.17 | 226.60 | 227.00 | -3.83 | -1.72 |
| Ethoxybenzene | 228.50 | 228.10 | 228.50 | 0.00 | 0.00 |
| Heptamethylenimine | 230.00 | 228.20 | 226.00 | 4.00 | 1.74 |
| 3,4-Dihydro-2H-1-benzopyran | 214.00 | 229.00 | 230.80 | -16.80 | -7.85 |
| Diisopropyl sulfide | 232.00 | 229.10 | 225.40 | 6.60 | 2.84 |
| Ethyl cyclobutanecarboxylate | 213.00 | 230.00 | 231.50 | -18.50 | -8.69 |
| Ethyl trichloroacetate | 230.10 | 230.10 | 230.80 | -0.70 | -0.30 |
| Cyclohexanol | 213.40 | 230.40 | 229.20 | -15.80 | -7.40 |
| Dipropyl sulfide | 225.48 | 230.70 | 230.10 | -4.62 | -2.05 |
| trans-3-Hexen-1-ol | 237.20 | 230.90 | 230.50 | 6.70 | 2.82 |
| cis-3-Hexen-1-ol | 234.00 | 230.90 | 231.10 | 2.90 | 1.24 |
| Indole-3-carboxyaldehyde | 220.50 | 231.10 | 241.70 | -21.20 | -9.61 |
| 1,3-Butyleneglycol | 218.89 | 231.20 | 232.90 | -14.01 | -6.40 |
| 1,2-Butanediol | 230.82 | 231.20 | 232.00 | -1.18 | -0.51 |
| 1,4-Diisocyanatobutane | 225.10 | 231.30 | 236.20 | -11.10 | -4.93 |
| N,N-Dimethylaniline | 213.40 | 231.70 | 235.50 | -22.10 | -10.36 |
| DL-Lactic acid | 210.50 | 232.30 | 233.10 | -22.60 | -10.74 |
| Lactic acid | 210.50 | 232.30 | 233.10 | -22.60 | -10.74 |
| 3-Azabicyclo[3.2.2]nonane | 238.90 | 232.50 | 232.50 | 6.40 | 2.68 |
| 1,4-Diaminobutane | 236.30 | 232.50 | 233.20 | 3.10 | 1.31 |
| 2,4,4-Trimethyl-2-nitropentane | 232.50 | 232.50 |  | 0.00 | 0.00 |
| Tetramethylurea | 229.60 | 232.60 |  | -3.00 | -1.31 |
| 1-Hexanethiol | 230.71 | 233.50 | 233.80 | -3.09 | -1.34 |
| 2,3,4,5,6-Pentafluorotoluene | 225.80 | 233.90 | 235.00 | -9.20 | -4.07 |
| Phenyltrichlorosilane | 220.70 | 234.00 |  | -13.30 | -6.03 |
| 2-(Ethylamino)ethanol | 227.00 | 234.50 | 235.00 | -8.00 | -3.52 |
| 2,6-Xylidine | 238.86 | 234.90 | 234.30 | 4.56 | 1.91 |
| 3-Methyl-2-butanol | 245.90 | 235.80 | 235.50 | 10.40 | 4.23 |
| Cyclohexanemethanol | 236.50 | 236.10 | 235.40 | 1.10 | 0.47 |
| Allylcyclohexane | 233.50 | 236.40 | 237.10 | -3.60 | -1.54 |
| N-Ethylaniline | 239.30 | 236.40 | 237.00 | 2.30 | 0.96 |
| Tetrahydro-2H-pyran-2-methanol | 222.00 | 236.90 | 237.50 | -15.50 | -6.98 |
| 1,1-Diethoxyethane | 237.70 | 236.90 |  | 0.80 | 0.34 |
| Tetramethyoxysilane | 240.50 | 237.00 | 236.90 | 3.60 | 1.50 |
| Diethyleneglycol | 243.90 | 237.10 | 235.70 | 8.20 | 3.36 |
| 3,3-Dimethyl-1-butanol | 236.08 | 237.10 | 237.30 | -1.22 | -0.52 |
| 1,2,3,4-Tetrahydroquinoline | 236.04 | 237.40 | 238.70 | -2.66 | -1.13 |
| 2,2,3,3-Tetramethylbutane | 232.20 | 237.70 | 238.10 | -5.90 | -2.54 |
| 3-Pentanol | 239.70 | 237.80 | 237.40 | 2.30 | 0.96 |
| Diisobutylene | 240.20 | 237.80 | 238.50 | 1.70 | 0.71 |
| 2-Phenylethylamine | 239.24 | 237.80 | 238.00 | 1.24 | 0.52 |
| 3,4-Dimethylpentanal | 235.60 | 238.30 | 238.40 | -2.80 | -1.19 |
| 1,2,4,5-Tetramethylbenzene | 220.10 | 238.40 | 239.30 | -19.20 | -8.72 |
| Prehnitene | 244.30 | 238.40 | 238.00 | 6.30 | 2.58 |
| Glycidyl isopropyl ether | 259.04 | 238.70 | 235.30 | 23.74 | 9.16 |
| 3-Nitrobenzotrifluoride | 223.80 | 238.80 | 240.70 | -16.90 | -7.55 |
| 1,5-Pentanediol | 233.20 | 238.90 | 239.70 | -6.50 | -2.79 |
| 1-Phenyl-1-propanone | 243.20 | 239.00 | 239.10 | 4.10 | 1.69 |
| Levulinic acid | 246.70 | 239.10 | 238.70 | 8.00 | 3.24 |
| 2-Aminoisobutanol | 229.50 | 239.20 |  | -9.70 | -4.23 |
| Butylacetamide | 236.89 | 239.40 | 239.60 | -2.71 | -1.14 |
| trans-Decalin | 229.17 | 239.60 | 240.20 | -11.03 | -4.81 |
| cis-Decalin | 231.79 | 239.60 | 240.20 | -8.41 | -3.63 |
| Phenethyl alcohol | 252.64 | 239.80 | 239.90 | 12.74 | 5.04 |
| trans-2-Octene | 239.30 | 239.90 | 240.00 | -0.70 | -0.29 |
| 3-Methylhexanal | 246.00 | 240.30 | 239.40 | 6.60 | 2.68 |
| t-Butylbenzene | 241.59 | 240.50 | 240.60 | 0.99 | 0.41 |
| 2,4-Dimethyl-3-pentanone | 233.70 | 240.60 | 240.70 | -7.00 | -3.00 |
| Nitrocyclohexane | 258.32 | 240.70 |  | 17.62 | 6.82 |
| Dimethyl maleate | 263.20 | 241.50 | 240.10 | 23.10 | 8.78 |
| N-Methylvaleramide | 238.40 | 241.90 | 243.30 | -4.90 | -2.06 |
| 4-Octyne | 233.60 | 242.30 | 247.00 | -13.40 | -5.74 |
| n-Heptaldehyde | 230.78 | 242.30 | 242.80 | -12.02 | -5.21 |
| 2-Methyl-3-hexanone | 241.50 | 242.60 | 242.40 | -0.90 | -0.37 |
| Biphenyl | 250.90 | 243.20 | 242.60 | 8.30 | 3.31 |
| Dipropylamine | 252.70 | 243.40 | 241.80 | 10.90 | 4.31 |
| Butanedioic acid | 238.60 | 243.40 | 243.00 | -4.40 | -1.84 |
| n-Hexylamine | 246.00 | 243.40 | 243.10 | 2.90 | 1.18 |
| p-Cymene | 242.30 | 243.40 | 242.50 | -0.20 | -0.08 |
| 2-Methyl-1-pentanol | 248.40 | 243.50 | 243.50 | 4.90 | 1.97 |
| Bicyclopentyl | 238.90 | 243.50 | 243.40 | -4.50 | -1.88 |
| 2-Ethyl-1-butanol | 246.65 | 243.50 | 243.60 | 3.05 | 1.24 |
| 2-Propoxyethanol | 241.60 | 243.70 | 243.90 | -2.30 | -0.95 |
| 2,2,3-Trimethylpentane | 245.60 | 244.10 | 244.00 | 1.60 | 0.65 |
| 2,3,3-Trimethylpentane | 245.56 | 244.10 | 244.00 | 1.56 | 0.64 |
| Isooctane | 242.49 | 244.10 | 243.40 | -0.91 | -0.38 |
| 2-Isopropoxyethanol | 238.80 | 244.30 | 245.00 | -6.20 | -2.60 |
| 4-Heptanone | 246.30 | 244.60 | 244.00 | 2.30 | 0.93 |
| 2-Heptanone | 243.60 | 244.60 | 244.30 | -0.70 | -0.29 |
| Ethyl acetoacetate | 241.80 | 244.90 | 245.20 | -3.40 | -1.41 |
| Propylcyclohexane | 242.04 | 245.00 | 245.00 | -2.96 | -1.22 |
| 1,1-Dimethylindane | 249.40 | 245.30 | 245.20 | 4.20 | 1.68 |
| Methyl levulinate | 231.10 | 245.40 | 245.50 | -14.40 | -6.23 |
| 1-Hexanol | 242.70 | 245.50 | 245.80 | -3.10 | -1.28 |
| Caprylene | 241.21 | 245.80 | 245.70 | -4.49 | -1.86 |
| n-Butyl acrylate | 251.00 | 246.10 | 246.80 | 4.20 | 1.67 |
| 3,3-Dimethylhexane | 246.60 | 246.10 | 246.20 | 0.40 | 0.16 |
| Methyl o-toluate | 242.50 | 246.20 | 246.20 | -3.70 | -1.53 |
| Methyl m-toluate | 248.70 | 246.20 | 246.00 | 2.70 | 1.09 |
| Ethyl pivalate | 251.50 | 246.40 | 246.80 | 4.70 | 1.87 |
| 3-(Dimethylamino)propylamine | 248.71 | 246.60 | 244.40 | 4.31 | 1.73 |
| 4,7-Dimethylindane | 241.50 | 246.70 | 246.90 | -5.40 | -2.24 |
| 4,6-Dimethylindane | 240.90 | 246.70 | 246.20 | -5.30 | -2.20 |
| 1,6-Dichlorohexane | 240.39 | 247.60 | 247.30 | -6.91 | -2.87 |
| Dihydropinene | 251.26 | 247.80 | 248.40 | 2.86 | 1.14 |
| Ethyl 2,3-dichloropropionate | 248.90 | 247.80 | 247.40 | 1.50 | 0.60 |
| Cyclohexyl acetate | 245.40 | 247.90 | 248.30 | -2.90 | -1.18 |
| 4-Methoxyaniline | 236.00 | 248.20 | 250.80 | -14.80 | -6.27 |
| 2,7-Dimethylnaphthalene | 251.85 | 248.40 | 247.80 | 4.05 | 1.61 |
| 2,3,4-Trimethylpentane | 247.32 | 248.50 | 249.40 | -2.08 | -0.84 |
| Butylbenzene | 243.50 | 248.80 | 248.40 | -4.90 | -2.01 |
| n-Hexanoic acid | 225.10 | 248.90 | 249.40 | -24.30 | -10.80 |
| Butylcyclopentane | 245.35 | 249.00 | 248.70 | -3.35 | -1.37 |
| Trimethylethylenediamine | 255.60 | 249.10 | 246.00 | 9.60 | 3.76 |
| Ethyl benzoate | 246.00 | 249.10 | 248.50 | -2.50 | -1.02 |
| Benzyl acetate | 250.20 | 249.10 | 249.50 | 0.70 | 0.28 |
| 1,1-Dimethoxy-3-cyanopropane | 253.60 | 249.50 |  | 4.10 | 1.62 |
| 1-Octyne | 242.14 | 250.10 | 252.70 | -10.56 | -4.36 |
| Ethyl t-amyl ether | 243.00 | 250.20 | 250.50 | -7.50 | -3.09 |
| 2,5-Dimethylhexane | 249.20 | 250.50 | 249.20 | 0.00 | 0.00 |
| Diethyl cellosolve | 259.40 | 250.90 | 251.20 | 8.20 | 3.16 |
| 1-Chloroheptane | 245.71 | 251.00 | 251.10 | -5.39 | -2.19 |
| Heptyl bromide | 249.41 | 251.30 | 252.20 | -2.79 | -1.12 |
| 2,5-Dioxaoctane | 248.90 | 251.30 | 251.40 | -2.50 | -1.00 |
| Phthaloyl chloride | 248.50 | 252.20 | 267.20 | -18.70 | -7.53 |
| 3-Methylheptane | 250.20 | 252.40 | 252.80 | -2.60 | -1.04 |
| 4-Methylheptane | 251.08 | 252.40 | 252.90 | -1.82 | -0.72 |
| 2-Methylheptane | 252.00 | 252.40 | 252.20 | -0.20 | -0.08 |
| Isoamyl acetate | 251.20 | 252.80 | 253.00 | -1.80 | -0.72 |
| 3-Carene | 254.26 | 253.00 | 254.50 | -0.24 | -0.09 |
| 2,4-Dibromophenol | 259.40 | 253.30 | 251.80 | 7.60 | 2.93 |
| Dimethyl methylmalonate | 261.70 | 254.10 | 254.50 | 7.20 | 2.75 |
| Octane | 255.68 | 254.40 | 254.50 | 1.18 | 0.46 |
| Amyl acetate | 276.10 | 254.80 | 254.80 | 21.30 | 7.71 |
| Ethyl pentanoate | 256.81 | 254.80 | 254.90 | 1.91 | 0.74 |
| Ethanediol diacetate | 269.20 | 255.10 | 254.30 | 14.90 | 5.53 |
| Diethyl oxalate | 260.70 | 255.10 | 254.90 | 5.80 | 2.22 |
| 1-Iodoheptane | 251.80 | 255.10 | 255.20 | -3.40 | -1.35 |
| Methyl hexanoate | 245.30 | 255.20 | 255.00 | -9.70 | -3.95 |
| 1-Ethyl-3-methylimidazolium bromide | 264.80 | 256.40 | 254.70 | 10.10 | 3.81 |
| Cycloheptanol | 250.22 | 256.50 | 257.10 | -6.88 | -2.75 |
| 2-Methyl-2-butanol | 247.30 | 256.60 | 256.30 | -9.00 | -3.64 |
| Pentafluoronitrobenzene | 272.96 | 257.60 | 254.90 | 18.06 | 6.62 |
| Ethyl N,N-dimethylglycinate | 244.20 | 257.90 | 257.10 | -12.90 | -5.28 |
| Diethanolamine | 251.30 | 257.90 | 258.70 | -7.40 | -2.94 |
| N,N-Dimethyl-o-toluidine | 280.30 | 258.30 | 255.60 | 24.70 | 8.81 |
| cis-2-Methylcyclohexanol | 268.95 | 258.50 | 258.30 | 10.65 | 3.96 |
| trans-2-Methylcyclohexanol | 262.98 | 258.50 | 258.30 | 4.68 | 1.78 |
| 2-Methoxyphenol | 240.00 | 258.70 | 261.40 | -21.40 | -8.92 |
| trans-Decahydroquinoline | 254.81 | 258.90 | 260.90 | -6.09 | -2.39 |
| 1-Hexen-3-ol | 269.30 | 259.20 | 259.20 | 10.10 | 3.75 |
| 2,4-Dinitrotoluene | 255.20 | 259.30 | 259.90 | -4.70 | -1.84 |
| Sulcatone | 268.80 | 260.50 | 259.20 | 9.60 | 3.57 |
| Diglycolamine | 246.84 | 260.70 | 262.30 | -15.46 | -6.26 |
| s-Phenethyl alcohol | 257.45 | 262.20 | 263.70 | -6.25 | -2.43 |
| Dipropyl disulfide | 262.46 | 262.50 | 262.90 | -0.44 | -0.17 |
| 1-Amino-2-(2-hydroxyethoxy)ethane | 258.60 | 262.80 | 262.10 | -3.50 | -1.35 |
| 2-(Isopropylamino)ethanol | 258.82 | 262.90 | 264.30 | -5.48 | -2.12 |
| 1-Heptanethiol | 259.32 | 263.50 | 263.40 | -4.08 | -1.57 |
| Perfluorotoluene | 262.30 | 263.80 | 264.60 | -2.30 | -0.88 |
| Camphor | 271.20 | 264.70 | 264.50 | 6.70 | 2.47 |
| 2,4,6-Trimethyl-2-cyclohexen-1-one | 258.60 | 264.80 | 266.00 | -7.40 | -2.86 |
| 2,5,6-Trimethyl-2-cyclohexen-1-one | 258.40 | 264.80 | 264.50 | -6.10 | -2.36 |
| Veratrole | 274.00 | 265.10 | 260.80 | 13.20 | 4.82 |
| 1,3-Dimethyladamantane | 258.31 | 265.20 | 265.10 | -6.79 | -2.63 |
| Butanoic anhydride | 283.70 | 265.30 | 264.20 | 19.50 | 6.87 |
| 4-Ethylacetophenone | 260.70 | 265.60 | 265.70 | -5.00 | -1.92 |
| 3-Methyl-2-pentanol | 275.89 | 265.90 | 265.20 | 10.69 | 3.87 |
| 4-Methyl-2-pentanol | 272.34 | 265.90 | 265.10 | 7.24 | 2.66 |
| Cyclohexaneethanol | 266.00 | 266.20 | 266.10 | -0.10 | -0.04 |
| Fenchone | 269.80 | 266.40 | 266.20 | 3.60 | 1.33 |
| t-Butylcyclohexane | 264.80 | 266.80 | 266.40 | -1.60 | -0.60 |
| 2,2,3,3-Tetramethylpentane | 271.50 | 267.80 | 267.70 | 3.80 | 1.40 |
| 2,2,4,4-Tetramethylpentane | 266.30 | 267.80 | 268.10 | -1.80 | -0.68 |
| 2-Hexanol | 256.31 | 267.90 | 267.70 | -11.39 | -4.44 |
| 3-Phenylpropylamine | 265.59 | 267.90 | 267.90 | -2.31 | -0.87 |
| 3-Hexanol | 269.27 | 267.90 | 269.10 | 0.17 | 0.06 |
| Ethyl lactate | 254.00 | 268.20 | 269.60 | -15.60 | -6.14 |
| Bis(2-hydroxyethyl)methylamine | 262.20 | 269.50 | 269.30 | -7.10 | -2.71 |
| 2-Phenyltoluene | 275.77 | 269.80 | 270.20 | 5.57 | 2.02 |
| Hydrocinnamyl alcohol | 280.74 | 269.90 | 269.60 | 11.14 | 3.97 |
| N-Cyclopentylpiperidine | 282.02 | 270.00 | 268.60 | 13.42 | 4.76 |
| 1-Phenyl-1-methylsilacyclobutane | 273.20 | 270.00 | 272.20 | 1.00 | 0.37 |
| 2-Ethylhexanal | 267.80 | 270.40 | 271.80 | -4.00 | -1.49 |
| Neopentylbenzene | 271.90 | 270.50 | 270.40 | 1.50 | 0.55 |
| 1-Methyl-4-isopropylcyclohexene | 257.80 | 270.70 | 271.50 | -13.70 | -5.31 |
| 2-t-Butoxyethanol | 273.45 | 271.30 | 270.80 | 2.65 | 0.97 |
| 1-Ethyladamantane | 258.45 | 271.50 | 271.90 | -13.45 | -5.20 |
| Diphenyl ether | 268.60 | 272.20 | 271.30 | -2.70 | -1.01 |
| Caprylic aldehyde | 259.40 | 272.40 | 274.20 | -14.80 | -5.71 |
| 1,8-Cineole | 270.72 | 272.70 | 273.50 | -2.78 | -1.03 |
| Diphenylmethane | 266.10 | 273.20 | 273.70 | -7.60 | -2.86 |
| Pentanedioic acid | 270.50 | 273.50 | 276.00 | -5.50 | -2.03 |
| 2-Diethylaminoethanol | 281.20 | 273.60 | 272.50 | 8.70 | 3.09 |
| 2-(Phenoxymethyl)oxirane | 276.00 | 273.70 | 273.20 | 2.80 | 1.01 |
| Butylcellosolve | 271.66 | 273.80 | 273.50 | -1.84 | -0.68 |
| 2-Octanone | 273.26 | 274.60 | 274.70 | -1.44 | -0.53 |
| Ethyl levulinate | 276.40 | 275.00 | 274.60 | 1.80 | 0.65 |
| Butylcyclohexane | 271.04 | 275.10 | 275.20 | -4.16 | -1.53 |
| 2-Acetoxyanisole | 278.50 | 275.20 | 273.40 | 5.10 | 1.83 |
| Ethyl cyclohexanecarboxylate | 271.50 | 275.50 | 275.40 | -3.90 | -1.44 |
| 1-Heptanol | 274.81 | 275.50 | 274.90 | -0.09 | -0.03 |
| 1-Nonene | 270.36 | 275.80 | 275.40 | -5.04 | -1.86 |
| Tetraethylmethane | 278.20 | 276.10 | 276.50 | 1.70 | 0.61 |
| n-Butylmethacrylate | 273.80 | 276.60 | 277.30 | -3.50 | -1.28 |
| Di-t-butyl ether | 276.10 | 277.70 | 278.70 | -2.60 | -0.94 |
| Methyl dimethylmalonate | 259.30 | 277.80 | 278.70 | -19.40 | -7.48 |
| Diisobutyl ether | 276.08 | 278.70 | 278.30 | -2.22 | -0.80 |
| Dipropyl carbonate | 275.55 | 278.80 | 280.00 | -4.45 | -1.61 |
| Enanthic acid | 267.31 | 279.00 | 279.20 | -11.89 | -4.45 |
| 1-Methylcyclohexanol | 279.05 | 279.20 | 277.00 | 2.05 | 0.73 |
| 2-Phenoxyethanol | 294.63 | 279.30 | 277.10 | 17.53 | 5.95 |
| Dimethylphenylvinylsilane | 284.80 | 279.40 | 276.10 | 8.70 | 3.05 |
| Diethylene glycol dimethyl ether | 274.10 | 280.10 | 280.10 | -6.00 | -2.19 |
| 1-Chlorooctane | 275.03 | 281.10 | 280.80 | -5.77 | -2.10 |
| 1-Bromooctane | 272.16 | 281.30 | 281.90 | -9.74 | -3.58 |
| 2,5-Dioxanonane | 282.00 | 281.40 | 281.40 | 0.60 | 0.21 |
| Di-n-butylether | 278.16 | 282.70 | 282.90 | -4.74 | -1.70 |
| 1,2,3,4-Tetrahydrophenanthrene | 278.26 | 282.80 | 283.90 | -5.64 | -2.03 |
| Isopentyl propionate | 285.20 | 282.90 | 283.30 | 1.90 | 0.67 |
| Di-t-butylsulfide | 281.74 | 283.10 | 282.40 | -0.66 | -0.23 |
| 1-Piperazineethanamine | 284.90 | 283.20 | 279.00 | 5.90 | 2.07 |
| Nonane | 284.43 | 284.50 | 284.80 | -0.37 | -0.13 |
| n-Hexylacetate | 282.80 | 284.80 | 285.00 | -2.20 | -0.78 |
| Butyl butanoate | 282.00 | 284.80 | 284.10 | -2.10 | -0.74 |
| Diethyl malonate | 285.00 | 285.20 | 286.00 | -1.00 | -0.35 |
| Methylenanthate | 285.10 | 285.30 | 285.30 | -0.20 | -0.07 |
| 2-Piperidineethanol | 289.02 | 285.50 | 284.30 | 4.72 | 1.63 |
| 1-Propyl-3-methylimidazolium bromide | 281.40 | 286.40 | 287.60 | -6.20 | -2.20 |
| 3-Methyl-3-pentanol | 293.30 | 286.60 | 284.40 | 8.90 | 3.03 |
| 2-Methyl-2-pentanol | 289.03 | 286.60 | 287.40 | 1.63 | 0.56 |
| N,N-Diethylaniline | 274.50 | 286.80 | 288.90 | -14.40 | -5.25 |
| Ethyl salicylate | 283.07 | 287.90 | 307.90 | -24.83 | -8.77 |
| Carvone | 279.34 | 288.60 | 289.20 | -9.86 | -3.53 |
| Di-n-butylsulfide | 284.34 | 290.90 | 291.60 | -7.26 | -2.55 |
| Tolane | 297.50 | 291.10 | 287.60 | 9.90 | 3.33 |
| 1,2-Hexanediol | 293.10 | 291.30 | 290.90 | 2.20 | 0.75 |
| 1,6-Hexamethylene diisocyanate | 294.00 | 291.40 | 289.30 | 4.70 | 1.60 |
| Pulegone | 284.50 | 293.30 | 291.90 | -7.40 | -2.60 |
| Benzophenone | 289.59 | 293.40 | 294.40 | -4.81 | -1.66 |
| 1-Octanethiol | 300.40 | 293.60 | 292.70 | 7.70 | 2.56 |
| 2,4-Dimethyl-3-pentanol | 312.00 | 294.00 | 292.80 | 19.20 | 6.15 |
| Ethyl trans-cinnamate | 296.36 | 294.70 | 294.10 | 2.26 | 0.76 |
| 1-(2-Aminoethyl)-4-methylpiperazine | 291.60 | 294.80 | 296.90 | -5.30 | -1.82 |
| 1,1-Diphenylethylene | 299.20 | 295.00 | 294.80 | 4.40 | 1.47 |
| Bicyclohexyl | 283.00 | 295.80 | 295.40 | -12.40 | -4.38 |
| 5-Methyl-2-hexanol | 295.20 | 295.90 | 297.20 | -2.00 | -0.68 |
| N-Cyclohexylpiperidine | 287.76 | 296.10 | 296.70 | -8.94 | -3.11 |
| Cyclohexanepropanol | 293.00 | 296.20 | 296.40 | -3.40 | -1.16 |
| 2,2'-Dimethylbiphenyl | 298.06 | 296.40 | 296.50 | 1.56 | 0.52 |
| 3,3'-Bitolyl | 295.45 | 296.40 | 295.80 | -0.35 | -0.12 |
| 2-Hydroxyethyl-2',2'-dimethylpropionate | 308.00 | 297.60 | 298.00 | 10.00 | 3.25 |
| 3-Heptanol | 314.20 | 297.90 | 297.50 | 16.70 | 5.32 |
| 4-Heptanol | 306.77 | 297.90 | 297.60 | 9.17 | 2.99 |
| 2-Heptanol | 298.63 | 297.90 | 298.40 | 0.23 | 0.08 |
| 1,1,4,7-Tetramethylindane | 302.50 | 298.50 | 298.30 | 4.20 | 1.39 |
| 2,3-Dihydro-1,1,4,6-tetramethyl-1H-indene | 299.60 | 298.50 | 298.70 | 0.90 | 0.30 |
| 1,7-Heptanediol | 297.00 | 299.00 | 299.00 | -2.00 | -0.67 |
| L-Menthone | 290.80 | 299.20 | 299.90 | -9.10 | -3.13 |
| Benzhydryl chloride | 290.40 | 299.70 | 300.70 | -10.30 | -3.55 |
| 2-Methyldiphenylmethane | 296.58 | 299.80 | 299.70 | -3.12 | -1.05 |
| 2-Ethylbiphenyl | 302.73 | 299.80 | 299.90 | 2.83 | 0.93 |
| 1-Ethyl-3-methylimidazolium thiocyanate | 281.45 | 300.00 |  | -18.55 | -6.59 |
| 2,6-Dimethylheptan-4-one | 297.30 | 300.70 | 301.10 | -3.80 | -1.28 |
| 1,1-Diphenylethane | 295.00 | 301.30 | 301.80 | -6.80 | -2.31 |
| Tetraethylsilane | 297.70 | 301.90 | 314.50 | -16.80 | -5.64 |
| n-Nonanal | 290.40 | 302.40 | 302.60 | -12.20 | -4.20 |
| 1,2-Diphenylethane | 320.10 | 303.30 | 302.60 | 17.50 | 5.47 |
| Di-n-butylamine | 292.90 | 303.50 | 304.90 | -12.00 | -4.10 |
| 2-Ethyl-1-hexanol | 317.50 | 303.60 | 303.30 | 14.20 | 4.47 |
| 2-Methyl-1-heptanol | 313.00 | 303.60 | 303.60 | 9.40 | 3.00 |
| n-Octylamine | 302.00 | 303.60 | 303.50 | -1.50 | -0.50 |
| 5-Methyl-1-heptanol | 304.20 | 303.60 | 304.10 | 0.10 | 0.03 |
| Neral | 304.60 | 304.20 | 304.00 | 0.60 | 0.20 |
| 5-Nonanone | 303.60 | 304.70 | 304.80 | -1.20 | -0.40 |
| Propyl levulinate | 317.00 | 305.10 | 304.70 | 12.30 | 3.88 |
| cis,trans,trans-1,5,9-Cyclododecatriene | 287.76 | 305.30 | 306.00 | -18.24 | -6.34 |
| Hexamethyldisiloxane | 311.30 | 305.30 |  | 6.00 | 1.93 |
| 1-Octanol | 312.10 | 305.60 | 305.60 | 6.50 | 2.08 |
| 1-Decene | 300.83 | 305.90 | 305.60 | -4.77 | -1.59 |
| Dimethyl phthalate | 303.10 | 307.10 | 307.40 | -4.30 | -1.42 |
| 1-Ethyl-3-methylimidazolium tetrafluoroborate | 308.10 | 307.60 | 310.80 | -2.70 | -0.88 |
| Cyclohexyl butanoate | 300.70 | 308.10 | 307.50 | -6.80 | -2.26 |
| n-Octanoic acid | 304.00 | 309.00 | 310.30 | -6.30 | -2.07 |
| Ethyl hydrocinnamate | 286.60 | 309.30 | 309.40 | -22.80 | -7.96 |
| 2,4,5-Trinitrotoluene | 311.70 | 309.60 | 312.90 | -1.20 | -0.38 |
| 1,2-Dipropoxyethane | 309.00 | 311.00 | 311.50 | -2.50 | -0.81 |
| 1-Chlorononane | 296.44 | 311.10 | 311.40 | -14.96 | -5.05 |
| 1-Bromononane | 308.32 | 311.40 | 311.50 | -3.18 | -1.03 |
| Tripropylamine | 301.00 | 312.60 | 313.90 | -12.90 | -4.29 |
| 4-Methylnonane | 317.36 | 312.60 | 312.20 | 5.16 | 1.63 |
| 3-Methylnonane | 308.99 | 312.60 | 312.20 | -3.21 | -1.04 |
| 5-Methylnonane | 314.43 | 312.60 | 311.60 | 2.83 | 0.90 |
| 2-Methylnonane | 313.30 | 312.60 | 312.90 | 0.40 | 0.13 |
| 1-Ethyl-3-methylimidazolium dicyanamide | 314.64 | 313.00 | 312.70 | 1.94 | 0.62 |
| Benzyldimethylvinylsilane | 312.20 | 314.50 | 315.50 | -3.30 | -1.06 |
| Decane | 316.32 | 314.50 | 314.50 | 1.82 | 0.58 |
| Di-t-butyldisulfide | 313.63 | 314.80 | 315.90 | -2.27 | -0.72 |
| Pentyl butyrate | 311.70 | 314.90 | 314.70 | -3.00 | -0.96 |
| Ethylene glycol dipropionate | 331.80 | 315.30 | 315.00 | 16.80 | 5.06 |
| Diethyl succinate | 330.50 | 315.30 | 314.40 | 16.10 | 4.87 |
| Methyl octanoate | 314.23 | 315.40 | 315.40 | -1.17 | -0.37 |
| 1-Butyl-3-methylimidazolium iodide | 314.00 | 316.50 | 317.00 | -3.00 | -0.96 |
| 1-Butyl-3-methylimidazolium chloride | 317.00 | 316.50 | 317.70 | -0.70 | -0.22 |
| 1-Butyl-3-methylimidazolium bromide | 316.70 | 316.50 | 316.50 | 0.20 | 0.06 |
| 2-Methyl-2-hexanol | 313.54 | 316.70 | 317.10 | -3.56 | -1.14 |
| 3-Methylbenzophenone | 325.10 | 320.00 | 320.10 | 5.00 | 1.54 |
| Dipropylene glycol | 322.10 | 320.20 | 319.80 | 2.30 | 0.71 |
| Triethanolamine | 309.27 | 320.50 | 324.10 | -14.83 | -4.80 |
| Diphenylmethylamine | 301.20 | 320.70 | 321.80 | -20.60 | -6.84 |
| 1-Ethyl-3-methylimidazolium acetate | 321.90 | 321.00 | 320.50 | 1.40 | 0.43 |
| 2,5-Dimethyl-3-hexanol | 339.40 | 324.00 | 325.60 | 13.80 | 4.07 |
| Triethyleneglycol | 327.60 | 325.50 | 325.30 | 2.30 | 0.70 |
| 4-Methyl-3-heptanol | 309.20 | 326.00 | 327.10 | -17.90 | -5.79 |
| 6-Methyl-3-heptanol | 310.50 | 326.00 | 325.90 | -15.40 | -4.96 |
| 4-Methyl-2-heptanol | 312.50 | 326.00 | 325.90 | -13.40 | -4.29 |
| 6-Methyl-2-heptanol | 315.10 | 326.00 | 325.90 | -10.80 | -3.43 |
| 2-Methyl-4-heptanol | 331.80 | 326.00 | 327.20 | 4.60 | 1.39 |
| 1,3-Dimethylimidazolium methosulfate | 341.00 | 326.20 | 325.40 | 15.60 | 4.57 |
| 4-Isopropylbiphenyl | 343.90 | 327.90 | 327.00 | 16.90 | 4.91 |
| 3-Octanol | 338.50 | 328.00 | 328.50 | 10.00 | 2.95 |
| 4-Octanol | 332.09 | 328.00 | 327.70 | 4.39 | 1.32 |
| 2-Octanol | 330.10 | 328.00 | 327.90 | 2.20 | 0.67 |
| Benzyl ether | 307.00 | 331.60 | 332.60 | -25.60 | -8.34 |
| Capraldehyde | 319.70 | 332.50 | 334.40 | -14.70 | -4.60 |
| N-Methyl-2-hydroxyethylammonium propionate | 328.00 | 333.30 | 335.90 | -7.90 | -2.41 |
| Heptanedioic acid | 334.30 | 333.60 | 333.70 | 0.60 | 0.18 |
| Butyl 4-oxopentanoate | 333.70 | 335.10 | 335.10 | -1.40 | -0.42 |
| 1-Nonanol | 341.00 | 335.70 | 335.10 | 5.90 | 1.73 |
| 1-Undecene | 329.95 | 335.90 | 335.60 | -5.65 | -1.71 |
| 4-Allyl-2-methoxyphenol | 343.10 | 336.80 | 335.50 | 7.60 | 2.22 |
| trans-Geraniol | 346.10 | 337.40 | 336.50 | 9.60 | 2.77 |
| 5-Cyano-5H-dibenzo[a,d]cycloheptene | 347.50 | 337.80 | 337.10 | 10.40 | 2.99 |
| Cyclohexyl valerate | 323.00 | 338.10 | 338.10 | -15.10 | -4.67 |
| Isopulegol | 357.60 | 338.40 | 336.60 | 21.00 | 5.87 |
| Pelargonic acid | 362.37 | 339.10 | 338.80 | 23.57 | 6.50 |
| Diethyleneglycol diethyl ether | 347.50 | 339.30 | 339.40 | 8.10 | 2.33 |
| 1-Ethyltetrahydrothiophenium dicyanamide | 335.38 | 339.60 | 341.60 | -6.22 | -1.85 |
| 1-Benzyl-3-methylimidazolium chloride | 339.40 | 340.90 | 340.90 | -1.50 | -0.44 |
| 1-Chlorodecane | 335.37 | 341.20 | 340.80 | -5.43 | -1.62 |
| 1-Bromodecane | 338.73 | 341.40 | 341.20 | -2.47 | -0.73 |
| Perfluoromethylcyclohexane | 353.10 | 341.50 |  | 11.60 | 3.29 |
| 2-Methyldecane | 341.21 | 342.60 | 342.90 | -1.69 | -0.50 |
| Tetraethylurea | 345.70 | 342.70 |  | 3.00 | 0.87 |
| Undecane | 345.05 | 344.60 | 344.60 | 0.45 | 0.13 |
| n-Octylacetate | 339.70 | 345.00 | 344.70 | -5.00 | -1.47 |
| Ethyl caprylate | 343.13 | 345.00 | 345.20 | -2.07 | -0.60 |
| 1-Ethyl-3-methylimidazolium methanesulfonate | 345.50 | 345.40 | 333.30 | 12.20 | 3.53 |
| Methyl nonanoate | 345.63 | 345.40 | 345.50 | 0.13 | 0.04 |
| delta-Undecanolactone | 342.70 | 346.00 | 346.10 | -3.40 | -0.99 |
| 4-Methyl-4-heptanol | 366.90 | 346.80 | 346.50 | 20.40 | 5.56 |
| 2-Methyl-2-heptanol | 337.60 | 346.80 | 346.50 | -8.90 | -2.64 |
| 1-Ethylpyridinium triflate | 351.80 | 348.60 | 347.80 | 4.00 | 1.14 |
| 1,4-Di-t-butylbenzene | 347.84 | 348.90 | 348.80 | -0.96 | -0.28 |
| beta-Citronellol | 357.90 | 349.60 | 350.40 | 7.50 | 2.10 |
| 1-Ethyl-3-methylimidazolium hexafluorophosphate | 343.60 | 352.90 | 352.50 | -8.90 | -2.59 |
| 1-Ethyl-3-methylimidazolium methylsulfate | 341.00 | 353.70 | 356.00 | -15.00 | -4.40 |
| 1-Decanethiol | 350.41 | 353.70 | 354.40 | -3.99 | -1.14 |
| o-Terphenyl | 369.05 | 354.30 | 353.80 | 15.25 | 4.13 |
| 2,3:7,8-Dibenzocycloocta-2,7-dien-1-one | 364.80 | 354.90 | 353.60 | 11.20 | 3.07 |
| Tetraethoxysilane | 364.40 | 355.40 | 357.50 | 6.90 | 1.89 |
| 2,2,4,6,6-Pentamethylheptane | 350.98 | 356.00 | 355.80 | -4.82 | -1.37 |
| Isoamyl lactate | 364.02 | 356.40 | 355.80 | 8.22 | 2.26 |
| Dibutyl acetal | 352.20 | 357.10 |  | -4.90 | -1.39 |
| 3-Nonanol | 373.63 | 358.00 | 359.20 | 14.43 | 3.86 |
| 5-Nonanol | 370.75 | 358.00 | 357.10 | 13.65 | 3.68 |
| 4-Nonanol | 367.86 | 358.00 | 358.00 | 9.86 | 2.68 |
| 2-Nonanol | 356.32 | 358.00 | 358.90 | -2.58 | -0.72 |
| 1-Butyl-3-methylimidazolium thiocyanate | 385.00 | 360.10 |  | 24.90 | 6.47 |
| 2-(2-(2-Methoxyethoxy)ethoxy)ethanol | 357.05 | 360.80 | 362.00 | -4.95 | -1.39 |
| 3,7-Dimethyl-1-octanol | 367.21 | 361.80 | 361.70 | 5.51 | 1.50 |
| Diethyleneglycol monobutyl ether | 354.89 | 362.20 | 362.40 | -7.51 | -2.12 |
| beta-Ionone | 382.80 | 362.60 | 361.00 | 21.80 | 5.69 |
| N-Methyl-2-hydroxyethylammonium butanoate | 361.00 | 363.30 | 364.50 | -3.50 | -0.97 |
| Octanedioic acid | 366.20 | 363.60 | 362.40 | 3.80 | 1.04 |
| n-Decylamine | 361.00 | 363.70 | 363.70 | -2.70 | -0.75 |
| 1-Ethyl-3-methylimidazolium trifluoromethylsulfonate | 362.80 | 363.80 | 364.00 | -1.20 | -0.33 |
| 2-Ethylhexyl acrylate | 355.10 | 364.40 | 363.70 | -8.60 | -2.42 |
| 6-Undecanone | 362.70 | 364.80 | 365.00 | -2.30 | -0.63 |
| Heptylcyclohexane | 363.20 | 365.30 | 365.40 | -2.20 | -0.61 |
| Hexamethylcyclotrisiloxane | 360.00 | 365.50 | 361.60 | -1.60 | -0.44 |
| n-Decyl alcohol | 377.00 | 365.70 | 364.90 | 12.10 | 3.21 |
| 1-Dodecene | 360.66 | 366.00 | 366.00 | -5.34 | -1.48 |
| Diethyl terephthalate | 380.70 | 366.30 | 365.90 | 14.80 | 3.89 |
| Diethyl phthalate | 366.10 | 366.30 | 367.70 | -1.60 | -0.44 |
| 1-Butyl-3-methylimidazolium tetrafluoroborate | 364.80 | 367.70 | 367.80 | -3.00 | -0.82 |
| 2,5,8,11-Tetraoxododecane | 367.78 | 368.50 | 368.50 | -0.72 | -0.20 |
| n-Decanoic acid | 361.10 | 369.20 | 368.90 | -7.80 | -2.16 |
| 1,2-Dibutoxyethane | 350.00 | 371.10 | 370.80 | -20.80 | -5.94 |
| 1-Bromoundecane | 373.23 | 371.50 | 370.00 | 3.23 | 0.87 |
| 1-Butyl-3-methylimidazolium dicyanoamide | 365.00 | 373.10 | 375.60 | -10.60 | -2.90 |
| 1-Methoxydecane | 370.80 | 373.30 | 372.70 | -1.90 | -0.51 |
| Tridecanolactone | 398.30 | 374.20 | 372.30 | 26.00 | 6.53 |
| Dodecane | 376.10 | 374.70 | 374.70 | 1.40 | 0.37 |
| Ethylene glycol dibutyrate | 380.30 | 375.40 | 376.20 | 4.10 | 1.08 |
| Methyl decanoate | 372.03 | 375.50 | 375.50 | -3.47 | -0.93 |
| Glyceryltriacetate | 384.70 | 376.30 | 375.40 | 9.30 | 2.42 |
| 1-Butyl-3-methylpyridinium tetrafluoroborate | 388.00 | 379.10 | 376.40 | 11.60 | 2.99 |
| 1-Butyl-3-methylimidazolium acetate | 383.20 | 381.20 | 379.70 | 3.50 | 0.91 |
| Pseudoionone | 382.70 | 382.10 | 383.00 | -0.30 | -0.08 |
| 1-Propyl-3-methylimidazolium hexafluorophosphate | 374.40 | 383.00 | 383.30 | -8.90 | -2.38 |
| 1-Ethyl-3-methylimidazolium ethosulfate | 378.00 | 383.30 | 382.10 | -4.10 | -1.08 |
| Linalool | 372.40 | 384.10 | 385.00 | -12.60 | -3.38 |
| 2-(2'-Hydroxyethoxy)ethyl pivalate | 368.83 | 386.00 | 386.40 | -17.57 | -4.76 |
| 5-Decanol | 405.77 | 388.10 | 387.70 | 18.07 | 4.45 |
| 3,7-Dimethyl-6-octen-1-yn-3-ol | 385.30 | 388.40 | 389.70 | -4.40 | -1.14 |
| 6,10-Dimethyl-4,5,9-undecatrien-2-one | 382.70 | 391.80 | 395.40 | -12.70 | -3.32 |
| 1-Benzyl-3-methylimidazolium tetrafluoroborate | 387.50 | 392.10 | 392.80 | -5.30 | -1.37 |
| Diethylene glycol monopentyl ether | 373.62 | 392.20 | 392.40 | -18.78 | -5.03 |
| N-((2-Aminoethyl)-2-aminoethyl)piperazine | 391.00 | 392.50 | 393.70 | -2.70 | -0.69 |
| N-Methyl-2-hydroxyethylammonium pentanoate | 401.00 | 393.40 | 389.60 | 11.40 | 2.84 |
| Nonanedioic acid | 398.10 | 393.70 | 392.50 | 5.60 | 1.41 |
| 1-Ethyl-3-methylpyridinium ethylsulfate | 389.00 | 394.70 | 398.00 | -9.00 | -2.31 |
| 1-Undecanol | 406.34 | 395.80 | 395.20 | 11.14 | 2.74 |
| 1-Tridecene | 391.80 | 396.10 | 395.70 | -3.90 | -1.00 |
| Nonyl acrylate | 417.90 | 396.40 | 395.70 | 22.20 | 5.31 |
| Octyl methacrylate | 386.00 | 396.80 | 396.50 | -10.50 | -2.72 |
| 1-Butyl-1-methylpyrrolidinium dicyanamide | 413.00 | 397.80 | 391.50 | 21.50 | 5.21 |
| Dibenzo[a,e]cyclooctene-5,11-dicarbonitrile | 417.00 | 397.90 | 395.70 | 21.30 | 5.11 |
| n-Undecanoic acid | 415.10 | 399.20 | 399.60 | 15.50 | 3.73 |
| 1,3-Bis(trimethylsilyl)propane | 394.34 | 399.30 | 403.30 | -8.96 | -2.27 |
| 1-Butyltetrahydrothiophenium dicyanamide | 395.19 | 399.70 | 400.70 | -5.51 | -1.40 |
| 1-Bromododecane | 399.31 | 401.60 | 402.40 | -3.09 | -0.77 |
| Tributylamine | 391.83 | 402.80 | 403.30 | -11.47 | -2.93 |
| Tris-(carboethoxy)methane | 419.80 | 403.80 | 402.90 | 16.90 | 4.03 |
| Tridecane | 407.10 | 404.70 | 404.00 | 3.10 | 0.76 |
| Octyl butanoate | 383.28 | 405.10 | 405.60 | -22.32 | -5.82 |
| Hexyl hexanoate | 394.92 | 405.10 | 405.20 | -10.28 | -2.60 |
| Butyl octanoate | 397.41 | 405.10 | 405.40 | -7.99 | -2.01 |
| Ethyl decanoate | 403.34 | 405.10 | 405.30 | -1.96 | -0.49 |
| Methyl undecanoate | 406.24 | 405.50 | 405.50 | 0.74 | 0.18 |
| 1-Butyl-3-methylimidazolium trifluoroacetate | 408.20 | 411.10 | 412.90 | -4.70 | -1.15 |
| 1,4-Bis-(1-isocyanato-1-methylethyl)benzene | 415.10 | 412.10 | 403.70 | 11.40 | 2.75 |
| 1-Butyl-3-methylimidazolium hexafluorophosphate | 407.70 | 413.00 | 414.20 | -6.50 | -1.59 |
| 1-Butyl-3-methylimidazolium methosulfate | 416.00 | 413.80 | 410.60 | 5.40 | 1.30 |
| Tetraglycol | 428.90 | 413.90 | 412.00 | 16.90 | 3.94 |
| Perfluorobicyclo[4.4.0]dec-1,6-ene | 428.40 | 416.00 | 417.40 | 11.00 | 2.57 |
| 6,10-Dimethyl-2-undecanone | 428.40 | 421.00 | 420.50 | 7.90 | 1.84 |
| Decanedioic acid | 430.00 | 423.80 | 422.70 | 7.30 | 1.70 |
| 1-Butyl-3-methylimidazolium trifluoromethylsulfonate | 417.00 | 423.90 | 426.00 | -9.00 | -2.16 |
| 1-Dodecanol | 438.42 | 425.80 | 425.80 | 12.62 | 2.88 |
| Oxacyclohexadecan-2-one | 444.60 | 426.40 | 424.30 | 20.30 | 4.57 |
| Nonyl methacrylate | 419.30 | 426.90 | 427.60 | -8.30 | -1.98 |
| 1-Hexyl-3-methylimidazolium tetrafluoroborate | 416.00 | 427.80 | 431.10 | -15.10 | -3.63 |
| Lauric acid | 404.28 | 429.30 | 429.70 | -25.42 | -6.29 |
| Decylcyclopentane | 426.52 | 429.30 | 429.50 | -2.98 | -0.70 |
| Tetradecane | 438.90 | 434.80 | 435.10 | 3.80 | 0.87 |
| Octamethyltrisiloxane | 420.60 | 435.00 |  | -14.40 | -3.42 |
| Dibutyl succinate | 436.24 | 435.50 | 434.50 | 1.74 | 0.40 |
| Methyl laurate | 432.68 | 435.60 | 435.50 | -2.82 | -0.65 |
| 1-Pentyl-3-methylimidazolium hexafluorophosphate | 437.40 | 443.10 | 443.80 | -6.40 | -1.46 |
| 1-Butyl-1-methylpyrrolidinium trifluoromethanesulfonate | 435.00 | 448.60 | 453.30 | -18.30 | -4.21 |
| Perfluoro-trans-decalin | 446.80 | 451.20 |  | -4.40 | -0.98 |
| Perfluoro-cis-decalin | 449.80 | 451.20 |  | -1.40 | -0.31 |
| Undecanedioic acid | 461.90 | 453.80 | 454.00 | 7.90 | 1.71 |
| Decylcyclohexane | 455.60 | 455.50 | 455.20 | 0.40 | 0.09 |
| 1-Tridecanol | 476.00 | 455.90 | 455.00 | 21.00 | 4.41 |
| Decyl methacrylate | 452.90 | 456.90 | 456.50 | -3.60 | -0.79 |
| 2,5,8,11,14-Pentaoxopentadecane | 457.10 | 456.90 | 456.50 | 0.60 | 0.13 |
| Diethyleneglycol-di-n-butylether | 452.00 | 459.50 | 460.10 | -8.10 | -1.79 |
| 1,4,7,10,13,16-Hexaoxacyclooctadecane | 460.00 | 459.80 | 460.60 | -0.60 | -0.13 |
| 1H,1H-Perfluorooctan-1-ol | 468.60 | 460.40 | 458.80 | 9.80 | 2.09 |
| 1-Bromotetradecane | 450.86 | 461.70 | 462.20 | -11.34 | -2.52 |
| 4-Cyano-3-fluorophenyl 4-butylbenzoate | 478.16 | 463.40 | 463.30 | 14.86 | 3.11 |
| Pentadecane | 469.90 | 464.80 | 464.80 | 5.10 | 1.09 |
| Ethyl dodecanoate | 464.00 | 465.20 | 465.00 | -1.00 | -0.22 |
| Methyl tridecanoate | 468.40 | 465.70 | 465.00 | 3.40 | 0.73 |
| Tripropionine | 481.30 | 466.50 | 467.70 | 13.60 | 2.83 |
| 2,2,4,4,6,8,8-Heptamethylnonane | 458.80 | 467.90 | 470.50 | -11.70 | -2.55 |
| Tetrapropoxysilane | 460.10 | 475.60 | 479.60 | -19.50 | -4.24 |
| Triethyleneglycol monopentylether | 479.80 | 480.60 | 480.80 | -1.00 | -0.21 |
| Dodecanedioic acid | 493.80 | 483.90 | 482.00 | 11.80 | 2.39 |
| 1-Hexyl-3-methylimidazolium trifluoromethylsulfonate | 502.30 | 484.00 | 479.30 | 23.00 | 4.58 |
| Myristyl alcohol | 505.80 | 486.00 | 485.70 | 20.10 | 3.97 |
| Cetene | 485.83 | 486.20 | 486.30 | -0.47 | -0.10 |
| 1-Ethyl-3-methylimidazolium toluenesulfonate | 484.20 | 486.30 | 488.30 | -4.10 | -0.85 |
| Dibutyl phthalate | 476.00 | 486.50 | 487.20 | -11.20 | -2.35 |
| Octamethylcyclotetrasiloxane | 495.94 | 487.30 | 482.10 | 13.84 | 2.79 |
| 1-Octyl-3-methylimidazolium tetrafluoroborate | 498.00 | 488.00 | 485.90 | 12.10 | 2.43 |
| N-Ethylpyridinium bis(trifluoromethylsulfonyl)amide | 502.15 | 491.50 | 490.70 | 11.45 | 2.28 |
| Cetane | 501.61 | 494.90 | 494.60 | 7.01 | 1.40 |
| Methyl tetradecanoate | 497.91 | 495.70 | 496.10 | 1.81 | 0.36 |
| Pentaglycol | 515.50 | 502.30 | 502.10 | 13.40 | 2.60 |
| 1-Heptyl-3-methylimidazolium hexafluorophosphate | 500.60 | 503.20 | 504.40 | -3.80 | -0.76 |
| Hexaethylcyclohexane | 530.10 | 505.70 | 503.60 | 26.50 | 5.00 |
| 1-Ethyl-3-methylimidazolium bis(trifluoromethanesulfonyl) amide | 500.00 | 506.70 | 507.10 | -7.10 | -1.42 |
| 1,1,1,3,5,5,5-Heptamethyl-3-phenyltrisiloxane | 519.60 | 511.20 |  | 8.40 | 1.62 |
| 1H,1H-Perfluorononan-1-ol | 515.90 | 511.30 | 510.60 | 5.30 | 1.03 |
| 1,13-Tridecanedioic acid | 525.70 | 513.90 | 513.20 | 12.50 | 2.38 |
| 1-Pentadecanol | 535.10 | 516.00 | 516.10 | 19.00 | 3.55 |
| Heptadecane | 534.34 | 525.00 | 525.10 | 9.24 | 1.73 |
| Ethyl myristate | 526.32 | 525.30 | 525.30 | 1.02 | 0.19 |
| Methyl pentadecanoate | 523.00 | 525.80 | 525.20 | -2.20 | -0.42 |
| N,N-Dimethyl-2-pentylnonylamine | 538.10 | 526.10 | 524.80 | 13.30 | 2.47 |
| N-Octylisoquinolinium thiocyanate | 522.00 | 528.30 |  | -6.30 | -1.21 |
| 1-Ethyl-3-methylimidazolium 2-(2-methoxyethoxy)ethylsulfate | 526.00 | 530.50 | 531.50 | -5.50 | -1.05 |
| 1-Octyl-3-methylimidazolium hexafluorophosphate | 536.10 | 533.30 | 532.90 | 3.20 | 0.60 |
| 1-Isopropyl-3-methylimidazolium bis(trifluoromethanesulfonyl) amide | 529.90 | 535.10 | 538.60 | -8.70 | -1.64 |
| N-Ethyl-2-methylpyridinium bis(trifluoromethylsulfonyl)amide | 534.50 | 535.30 | 535.20 | -0.70 | -0.13 |
| 1-Propyl-3-methylimidazolium bis(trifluoromethanesulfonyl) amide | 534.90 | 536.70 | 536.70 | -1.80 | -0.34 |
| Pentaethylene glycol monomethyl ether | 537.12 | 537.60 | 536.80 | 0.32 | 0.06 |
| 6,10,14-Trimethylpentadeca-3,5-diene-2-one | 555.20 | 542.60 | 543.50 | 11.70 | 2.11 |
| 1,14-Tetradecanedioic acid | 557.60 | 544.00 | 542.20 | 15.40 | 2.76 |
| Pentaethylene glycol dimethyl ether | 547.40 | 545.30 | 544.10 | 3.30 | 0.60 |
| 1-Hexadecanol | 523.80 | 546.10 | 547.10 | -23.30 | -4.45 |
| 1-Butyl-3-methylimidazolium toluenesulfonate | 548.40 | 546.40 | 544.50 | 3.90 | 0.71 |
| 1-Cyclopropylmethyl-3-methylimidazolium bis(trifluoromethanesulfonyl) amide | 539.10 | 551.50 | 551.60 | -12.50 | -2.32 |
| N-Butylpyridinium bis(trifluoromethanesulfonyl) amide | 566.52 | 551.60 | 551.80 | 14.72 | 2.60 |
| Octadecane | 564.40 | 555.00 | 554.20 | 10.20 | 1.81 |
| Glyceryl tributanoate | 555.30 | 556.70 | 556.80 | -1.50 | -0.27 |
| Tetrapropylene glycol | 559.80 | 558.20 | 558.70 | 1.10 | 0.20 |
| Trimethyl butylammonium bis(trifluoromethylsulfonyl)amide | 559.20 | 561.10 | 574.30 | -15.10 | -2.70 |
| 1-Methyl-1-propylpyrrolidinium bis(trifluoromethanesulfonyl) amide | 554.00 | 561.40 | 562.00 | -8.00 | -1.44 |
| 1H,1H-Perfluorodecan-1-ol | 563.90 | 562.20 | 561.60 | 2.30 | 0.41 |
| 1-Nonyl-3-methylimidazolium hexafluorophosphate | 569.40 | 563.30 | 563.70 | 5.70 | 1.00 |
| 1-Isobutyl-3-methylimidazolium bis(trifluoromethanesulfonyl) amide | 557.10 | 564.80 | 565.30 | -8.20 | -1.47 |
| 1-sec-Butyl-3-methylimidazolium bis(trifluoromethanesulfonyl) amide | 557.10 | 565.10 | 568.70 | -11.60 | -2.08 |
| N-Propyl-2-methylpyridinium bis(trifluoromethylsulfonyl)amide | 557.96 | 565.30 | 566.40 | -8.44 | -1.51 |
| 1,2-Diethylpyridinium bis(trifluoromethanesulfonyl) amide | 566.10 | 565.30 | 565.30 | 0.80 | 0.14 |
| 1-Butyl-3-methylimidazolium bis(trifluoromethanesulfonyl) amide | 565.90 | 566.80 | 566.60 | -0.70 | -0.12 |
| 1-Isobutyl-3-methylpyridinium bis(trifluoromethylsulfonyl)amide | 579.00 | 576.20 | 576.10 | 2.90 | 0.50 |
| Pristane | 569.76 | 577.20 | 577.60 | -7.84 | -1.38 |
| N-Butyl-3-methylpyridinium bis(trifluoromethylsulfonyl)amide | 578.10 | 578.20 | 577.30 | 0.80 | 0.14 |
| 1-Chlorooctadecane | 606.70 | 581.70 | 580.90 | 25.80 | 4.25 |
| Trihexylamine | 568.12 | 583.10 | 583.70 | -15.58 | -2.74 |
| Nonadecane | 604.00 | 585.10 | 585.30 | 18.70 | 3.10 |
| 1-Isobutyl-1-methylpyrrolidinium bis(trifluoromethylsulfonyl)amide | 582.20 | 589.50 | 588.30 | -6.10 | -1.05 |
| 1-Butyl-3-cyanopyridinium bis(trifluoromethylsulfonyl)amide | 586.00 | 590.00 | 589.70 | -3.70 | -0.63 |
| 1-Benzyl-3-methylimidazolium bis(trifluoromethylsulfonyl) amide | 607.80 | 591.20 | 591.30 | 16.50 | 2.71 |
| N-Ethyl-4-dimethylaminopyridinium bis(trifluoromethanesulfonyl) amide | 594.30 | 591.20 | 590.30 | 4.00 | 0.67 |
| 1-Butyl-1-methylpyrrolidinium bis(trifluoromethylsulfonyl)amide | 589.30 | 591.50 | 591.70 | -2.40 | -0.41 |
| 1-Decyl-3-methylimidazolium hexafluorophosphate | 603.30 | 593.40 | 592.40 | 10.90 | 1.81 |
| 1-Butyltetrahydrothiophenium bis(trifluoromethylsulfonyl) amide | 596.00 | 593.40 | 592.50 | 3.50 | 0.59 |
| (9Z)-Octadecenoic acid | 577.00 | 595.10 | 595.90 | -18.90 | -3.28 |
| 1-Ethyl-2-propylpyridinium bis(trifluoromethanesulfonyl) amide | 593.90 | 595.40 | 596.50 | -2.60 | -0.44 |
| Tetrabutoxysilane | 580.20 | 595.90 | 597.80 | -17.60 | -3.03 |
| 1-Pentyl-3-methylimidazolium bis(trifluoromethanesulfonyl) amide | 595.60 | 596.90 | 597.00 | -1.40 | -0.24 |
| 1,1-Diphenyldodecane | 593.70 | 601.90 | 601.20 | -7.50 | -1.26 |
| Hexadecanedioic acid | 621.40 | 604.10 | 602.80 | 18.60 | 2.99 |
| N-Hexylpyridinium bis(trifluoromethanesulfonyl) amide | 612.00 | 611.70 | 613.40 | -1.40 | -0.23 |
| 1H,1H-Perfluoroundecan-1-ol | 602.40 | 613.10 | 615.50 | -13.10 | -2.17 |
| Dibutyl decanedioate | 619.00 | 615.90 | 615.50 | 3.50 | 0.57 |
| 1-Cyclohexylmethyl-3-methylimidazolium bis(trifluoromethanesulfonyl) amide | 617.10 | 617.50 | 617.90 | -0.80 | -0.13 |
| 1-Butyl-1-methylpiperidinium bis(trifluoromethylsulfonyl)amide | 607.50 | 617.60 | 618.20 | -10.70 | -1.76 |
| 1-Methyl-1-pentylpyrrolidinium bis(trifluoromethanesulfonyl) amide | 622.60 | 621.60 | 622.80 | -0.20 | -0.03 |
| 1-Pentyltetrahydrothiophenium bis(trifluoromethylsulfonyl) amide | 627.00 | 623.50 | 622.90 | 4.10 | 0.65 |
| 1-Butyl-3-methylimidazolium octylsulfate | 635.00 | 623.80 | 622.00 | 13.00 | 2.05 |
| 1,1,3,3-Tetraethyl-5,5-diphenylcylotrisiloxane | 629.60 | 624.80 | 638.70 | -9.10 | -1.45 |
| 1-Ethyl-2-butylpyridinium bis(trifluoromethanesulfonyl) amide | 623.60 | 625.50 | 625.70 | -2.10 | -0.34 |
| 1-Hexyl-3-methylimidazolium bis(trifluoromethanesulfonyl) amide | 629.20 | 626.90 | 627.80 | 1.40 | 0.22 |
| 1-Phenyl-1-cyclohexyldodecane | 611.10 | 628.20 | 628.20 | -17.10 | -2.80 |
| 1-Hexyl-3-methylpyridinium bis(trifluoromethylsulfonyl)amide | 624.00 | 638.30 | 639.10 | -15.10 | -2.42 |
| 1,1-Diphenyl-3,3,5,5,7,7-hexamethylcyclotetrasiloxane | 633.00 | 639.70 | 647.00 | -14.00 | -2.21 |
| 1-Hexyl-4-cyanopyridinium bis(trifluoromethylsulfonyl)amide | 633.00 | 650.10 | 649.70 | -16.70 | -2.64 |
| 1-Hexyl-3-cyanopyridinium bis(trifluoromethylsulfonyl)amide | 658.00 | 650.10 | 650.10 | 7.90 | 1.20 |
| N-Butyl-4-dimethylaminopyridinium bis(trifluoromethanesulfonyl) amide | 657.71 | 651.30 | 652.00 | 5.71 | 0.87 |
| 1-Hexyl-1-methylpyrrolidinium bis(trifluoromethanesulfonyl) amide | 655.10 | 651.60 | 649.50 | 5.60 | 0.85 |
| 1-Dodecyl-3-methylimidazolium hexafluorophosphate | 666.30 | 653.50 | 652.80 | 13.50 | 2.03 |
| 1-Hexyltetrahydrothiophenium bis(trifluoromethylsulfonyl) amide | 646.00 | 653.60 | 654.70 | -8.70 | -1.35 |
| 1-Ethyl-2-pentylpyridinium bis(trifluoromethanesulfonyl) amide | 652.70 | 655.50 | 655.80 | -3.10 | -0.47 |
| 1-Heptyl-3-methylimidazolium bis(trifluoromethanesulfonyl) amide | 659.20 | 657.00 | 657.50 | 1.70 | 0.26 |
| 1H,1H-Perfluorododecan-1-ol | 645.20 | 663.90 | 667.50 | -22.30 | -3.46 |
| Ricinelaidic acid | 646.00 | 668.70 | 669.60 | -23.60 | -3.65 |
| 1-Octylpyridinium bis(trifluoromethylsulfonyl)amide | 686.00 | 671.90 | 670.20 | 15.80 | 2.30 |
| Docosane | 698.00 | 675.30 | 674.30 | 23.70 | 3.40 |
| Pentapropylene glycol | 685.80 | 677.20 | 673.50 | 12.30 | 1.79 |
| 1-Methyl-1-heptylpyrrolidinium bis(trifluoromethanesulfonyl) amide | 685.10 | 681.70 | 682.50 | 2.60 | 0.38 |
| 1-Heptyltetrahydrothiophenium bis(trifluoromethylsulfonyl) amide | 685.00 | 683.60 | 683.60 | 1.40 | 0.20 |
| 1-Ethyl-2-hexylpyridinium bis(trifluoromethanesulfonyl) amide | 685.50 | 685.60 | 685.60 | -0.10 | -0.01 |
| 1-Octyl-3-methylimidazolium bis(trifluoromethanesulfonyl) amide | 690.20 | 687.00 | 686.60 | 3.60 | 0.52 |
| 3,7,11,15-Tetramethyl-1-hexadecyn-3-ol | 712.50 | 697.20 | 690.00 | 22.50 | 3.16 |
| 1-Octyl-3-cyanopyridinium bis(trifluoromethylsulfonyl)amide | 709.00 | 710.20 | 710.70 | -1.70 | -0.24 |
| 4-Dimethylamino-1-hexylpyridinium bis(trifluoromethanesulfonyl) amide | 731.00 | 711.50 | 708.30 | 22.70 | 3.11 |
| 1-Methyl-1-octylpyrrolidinium bis(trifluoromethanesulfonyl) amide | 716.30 | 711.70 | 711.20 | 5.10 | 0.71 |
| 1-Octyltetrahydrothiophenium bis(trifluoromethylsulfonyl) amide | 719.00 | 713.70 | 713.00 | 6.00 | 0.83 |
| 1-Ethyl-2-heptylpyridinium bis(trifluoromethanesulfonyl) amide | 717.50 | 715.60 | 715.50 | 2.00 | 0.28 |
| Heptylpentaoxyethylene | 722.90 | 717.50 | 717.00 | 5.90 | 0.82 |
| 1-(3,4,5,6-Perfluorohexyl)-3-methylimidazolium-3-methylimidazolium bis(trifluoromethanesulfonyl) amide | 725.00 | 719.30 | 719.80 | 5.20 | 0.72 |
| DEHP | 708.98 | 723.10 | 724.60 | -15.62 | -2.20 |
| Di-n-octyl phthalate | 707.10 | 727.00 | 728.00 | -20.90 | -2.96 |
| Bis(1-ethylhexyl) o-phthalate | 704.60 | 728.10 | 727.60 | -23.00 | -3.26 |
| Butyl 1-butylnicotinate bis(trifluoromethylsulfonyl)amide | 707.00 | 728.90 | 731.30 | -24.30 | -3.44 |
| Dihexyl sebacate | 710.90 | 736.10 | 735.70 | -24.80 | -3.49 |
| Glycerol tricaproate | 753.10 | 737.00 | 735.90 | 17.20 | 2.28 |
| N-Hexyl-3-methyl-4-dimethylaminopyridinium bis(trifluoromethanesulfonyl) amide | 725.00 | 738.10 | 741.00 | -16.00 | -2.21 |
| 1-Nonyltetrahydrothiophenium bis(trifluoromethylsulfonyl) amide | 741.00 | 743.70 | 744.40 | -3.40 | -0.46 |
| 1-Ethyl-2-octylpyridinium bis(trifluoromethanesulfonyl) amide | 749.40 | 745.70 | 745.10 | 4.30 | 0.57 |
| 1-Decyl-3-methylimidazolium bis(trifluoromethanesulfonyl) amide | 754.80 | 747.20 | 747.10 | 7.70 | 1.02 |
| Tri-n-octylamine | 750.80 | 763.50 | 765.70 | -14.90 | -1.98 |
| Pentacosane | 769.00 | 765.40 | 765.80 | 3.20 | 0.42 |
| 1-Methyl-1-decylpyrrolidinium bis(trifluoromethanesulfonyl) amide | 778.90 | 771.90 | 771.30 | 7.60 | 0.98 |
| 1-Decyltetrahydrothiophenium bis(trifluoromethylsulfonyl) amide | 780.00 | 773.80 | 773.20 | 6.80 | 0.87 |
| 1-Ethyl-2-nonylpyridinium bis(trifluoromethanesulfonyl) amide | 778.50 | 775.80 | 775.40 | 3.10 | 0.40 |
| 1-Methyl-3-menthyloxymethylimidazolium bis(trifluoromethylsulfonyl)imide | 781.40 | 785.90 | 786.30 | -4.90 | -0.63 |
| Hexapropylene glycol | 807.50 | 796.20 | 796.00 | 11.50 | 1.42 |
| 1-Ethyl-2-decylpyridinium bis(trifluoromethanesulfonyl) amide | 811.20 | 805.80 | 805.60 | 5.60 | 0.69 |
| 1-Dodecyl-3-methylimidazolium bis(trifluoromethanesulfonyl) amide | 820.20 | 807.30 | 806.60 | 13.60 | 1.66 |
| 1-Ethyl-3-menthyloxymethylimidazolium bis(trifluoromethylsulfonyl)imide | 818.40 | 813.40 | 813.90 | 4.50 | 0.55 |
| Bis(2-ethylhexyl) azelate | 799.70 | 822.30 | 823.50 | -23.80 | -2.98 |
| Heptacosane | 828.40 | 825.60 | 827.20 | 1.20 | 0.14 |
| 1-Propyl-3-menthyloxymethylimidazolium bis(trifluoromethylsulfonyl)imide | 839.40 | 843.40 | 844.00 | -4.60 | -0.55 |
| Bis(2-ethylhexyl) sebacate | 849.40 | 852.40 | 850.20 | -0.80 | -0.09 |
| 1-Butyl-3-menthyloxymethylimidazolium bis(trifluoromethylsulfonyl)imide | 866.20 | 873.50 | 874.20 | -8.00 | -0.92 |
| Vitamin E acetate | 897.90 | 888.10 | 889.40 | 8.50 | 0.95 |
| 1-Pentyl-3-menthyloxymethylimidazolium bis(trifluoromethylsulfonyl)imide | 905.00 | 903.60 | 904.30 | 0.70 | 0.08 |
| Dodecahydrosqualene | 886.36 | 903.90 | 906.10 | -19.74 | -2.23 |
| 1-Hexyl-3-menthyloxymethylimidazolium bis(trifluoromethylsulfonyl)imide | 944.60 | 933.60 | 932.60 | 12.00 | 1.27 |
| 11-Decylheneicosane | 949.80 | 943.80 | 944.30 | 5.50 | 0.58 |
| 1-Heptyl-3-menthyloxymethylimidazolium bis(trifluoromethylsulfonyl)imide | 960.50 | 963.70 | 963.60 | -3.10 | -0.32 |
| 1-Octyl-3-menthyloxymethylimidazolium bis(trifluoromethylsulfonyl)imide | 995.00 | 993.70 | 994.00 | 1.00 | 0.10 |
| 1-Nonyl-3-menthyloxymethylimidazolium bis(trifluoromethylsulfonyl)imide | 1023.60 | 1023.80 | 1023.90 | -0.30 | -0.03 |
| 1-Decyl-3-menthyloxymethylimidazolium bis(trifluoromethylsulfonyl)imide | 1045.90 | 1053.90 | 1054.60 | -8.70 | -0.83 |
| Tetraoctoxysilane | 1095.40 | 1076.80 | 1070.50 | 24.90 | 2.27 |
| 1-Undecyl-3-menthyloxymethylimidazolium bis(trifluoromethylsulfonyl)imide | 1088.30 | 1083.90 | 1084.10 | 4.20 | 0.39 |
| Glyceryl tricaprate | 1108.80 | 1097.70 | 1096.10 | 12.70 | 1.15 |
| 1-Dodecyl-3-menthyloxymethylimidazolium bis(trifluoromethylsulfonyl)imide | 1117.90 | 1114.00 | 1113.30 | 4.60 | 0.41 |
| Tetradecyl trihexylphosphonium bis(trifluoromethylsulfonyl)amide | 1298.80 | 1312.00 |  | -13.20 | -1.02 |
| Benzene hexa-n-hexanoate | 1300.00 | 1313.20 | 1324.30 | -24.30 | -1.87 |
| Benzene hexa-n-heptanoate | 1500.00 | 1493.60 | 1487.60 | 12.40 | 0.83 |
| TMPTO | 1849.00 | 1856.80 | 1859.50 | -10.50 | -0.57 |
|  |  |  |  |  |  |
