# Supplementary material for: Calculation of the Isobaric Heat Capacities of the Liquid and Solid Phase of Organic Compounds at 298.15K by Means of the Group-Additivity Method
Source: Molecules. 2020 Mar 4;25(5):1147. doi: 10.3390/molecules25051147 (PMC7179130; doi:10.3390/molecules25051147)
Supplement: Supplementary file 1 [file molecules-25-01147-s001.zip › S04. Experimental vs. calculated Cp(sol,298) Data Table.doc]

| Molecules sorted by calc values |  |  |  |  |  |
| --- | --- | --- | --- | --- | --- |
| Molecule name | Cp(sol,298) exp | Cp(sol,298) calc | Cp(sol,298) test | Deviation | Dev. in % |
| 1,2,4-Triazole | 78.70 | 70.80 | 54.40 | 24.30 | 30.88 |
| Imidazole | 83.93 | 86.10 | 86.20 | -2.27 | -2.70 |
| Urea | 93.80 | 86.70 | 86.70 | 7.10 | 7.57 |
| 1H-Pyrazole | 81.00 | 89.00 | 90.90 | -9.90 | -12.22 |
| Acetamide | 86.65 | 89.90 | 89.60 | -2.95 | -3.40 |
| Glycine | 99.30 | 89.90 | 89.30 | 10.00 | 10.07 |
| Methylammonium iodide | 93.93 | 92.90 |  | 1.03 | 1.10 |
| Pyrazine | 102.35 | 96.30 | 94.00 | 8.35 | 8.16 |
| Thiourea | 96.90 | 98.00 | 99.80 | -2.90 | -2.99 |
| Thioacetamide | 100.30 | 99.10 |  | 1.20 | 1.20 |
| Diformylhydrazine | 99.12 | 100.30 |  | -1.18 | -1.19 |
| Propanone | 96.20 | 101.90 | 102.00 | -5.80 | -6.03 |
| Hydrazinecarboxamide | 110.60 | 101.90 |  | 8.70 | 7.87 |
| Dicyanomethane | 110.29 | 104.70 | 104.90 | 5.39 | 4.89 |
| 1,3,5-Trioxane | 111.40 | 105.60 |  | 5.80 | 5.21 |
| Oxalamide | 110.86 | 105.60 | 105.00 | 5.86 | 5.29 |
| 2-Methylimidazole | 105.09 | 107.80 | 108.90 | -3.81 | -3.63 |
| Benzene | 97.90 | 107.80 | 107.00 | -9.10 | -9.30 |
| 1,3-Dioxolan-2-one | 117.12 | 108.40 | 105.20 | 11.92 | 10.18 |
| Acrylamide | 110.58 | 109.70 | 109.60 | 0.98 | 0.89 |
| 2,4-Imidazolidinedione | 117.09 | 109.90 | 108.80 | 8.29 | 7.08 |
| 3-Amino-5-methylisoxazole | 125.10 | 111.00 | 92.80 | 32.30 | 25.82 |
| Oxalic acid | 91.00 | 112.10 | 111.40 | -20.40 | -22.42 |
| Purine | 106.90 | 112.40 |  | -5.50 | -5.14 |
| Hydrazinecarbothioamide | 110.90 | 113.20 |  | -2.30 | -2.07 |
| Dimethylene urethane | 113.80 | 114.30 | 114.80 | -1.00 | -0.88 |
| 2,5-Furandione | 120.10 | 114.60 | 115.90 | 4.20 | 3.50 |
| beta-Alanine | 116.40 | 115.40 | 115.30 | 1.10 | 0.95 |
| Methylcarbamide | 114.74 | 115.70 | 117.00 | -2.26 | -1.97 |
| 1,3-Oxazolidine-2-thione | 117.10 | 115.80 |  | 1.30 | 1.11 |
| (L)-Alanine | 119.90 | 116.40 | 115.30 | 4.60 | 3.84 |
| Squaric acid | 121.80 | 116.70 | 105.90 | 15.90 | 13.05 |
| 2,5-Pyrrolidinedione | 123.88 | 118.70 | 118.20 | 5.68 | 4.59 |
| 2-Thioxo-4-imidazolidinone | 137.05 | 121.20 | 116.60 | 20.45 | 14.92 |
| Serine | 135.60 | 121.80 | 117.40 | 18.20 | 13.42 |
| Sarcosine | 118.20 | 122.40 |  | -4.20 | -3.55 |
| 1,4-Benzoquinone | 129.00 | 123.20 | 121.30 | 7.70 | 5.97 |
| Glycine anhydride | 134.00 | 123.70 | 121.90 | 12.10 | 9.03 |
| 1,3-Dithiane | 123.84 | 123.80 |  | 0.04 | 0.03 |
| Phenol | 127.44 | 123.80 | 124.20 | 3.24 | 2.54 |
| Uracil | 120.50 | 124.40 | 125.00 | -4.50 | -3.73 |
| Cytosine | 132.60 | 124.90 | 122.20 | 10.40 | 7.84 |
| 2-Mercapto-2-thiazoline | 125.30 | 125.50 |  | -0.20 | -0.16 |
| N-Methylmaleimide | 146.65 | 126.00 | 126.80 | 19.85 | 13.54 |
| 1,3-Dioxolan-2-one | 133.90 | 128.10 | 122.00 | 11.90 | 8.89 |
| Carbonothioic dihydrazide | 125.30 | 128.50 |  | -3.20 | -2.55 |
| Lactic acid | 127.60 | 128.70 | 129.30 | -1.70 | -1.33 |
| Nortricyclene | 129.00 | 130.50 | 129.40 | -0.40 | -0.31 |
| Hypoxanthine | 134.50 | 131.00 | 128.50 | 6.00 | 4.46 |
| Malonamide | 133.38 | 131.10 | 134.10 | -0.72 | -0.54 |
| 2-Aminopyridine | 142.47 | 132.40 | 125.40 | 17.07 | 11.98 |
| Propylenecarbonate | 122.51 | 132.70 | 135.20 | -12.69 | -10.36 |
| Methimazole | 129.80 | 132.80 | 137.20 | -7.40 | -5.70 |
| 2-Ethylimidazole | 147.08 | 133.20 | 127.00 | 20.08 | 13.65 |
| Imidodicarbonic diamide | 131.30 | 133.80 | 132.70 | -1.40 | -1.07 |
| 2,4-Hexadiyne | 133.57 | 134.00 |  | -0.43 | -0.32 |
| 2-Pyrazinecarboxylic acid | 143.50 | 134.00 | 131.10 | 12.40 | 8.64 |
| 2-Furoic acid | 132.26 | 135.40 |  | -3.14 | -2.37 |
| Tetrahydro-2H-1,3-oxazine-2-thione | 139.70 | 135.40 |  | 4.30 | 3.08 |
| 2-Methyl-2-propanol | 146.11 | 135.60 | 134.40 | 11.71 | 8.01 |
| Methacrylamide | 143.80 | 135.70 | 133.20 | 10.60 | 7.37 |
| Barbituric acid | 141.10 | 136.30 | 136.80 | 4.30 | 3.05 |
| 4-Fluorophenol | 144.60 | 136.90 | 136.50 | 8.10 | 5.60 |
| Proline | 150.40 | 137.20 |  | 13.20 | 8.78 |
| 5-Methylhydantoin | 143.11 | 137.30 | 136.60 | 6.51 | 4.55 |
| Cyanuric acid | 133.50 | 137.80 | 140.30 | -6.80 | -5.09 |
| Iodobenzene | 112.10 | 138.30 |  | -26.20 | -23.37 |
| Malonic acid | 127.63 | 138.70 | 137.10 | -9.47 | -7.42 |
| Isobutyramide | 148.10 | 139.00 | 139.10 | 9.00 | 6.08 |
| 1,3-Dihydroxybenzene | 135.53 | 139.80 | 138.30 | -2.77 | -2.04 |
| 1,4-Dihydroxybenzene | 136.40 | 139.80 | 139.80 | -3.40 | -2.49 |
| 1,2-Dihydroxybenzene | 144.30 | 139.80 | 139.20 | 5.10 | 3.53 |
| 4-Chlorophenol | 161.06 | 140.20 | 140.60 | 20.46 | 12.70 |
| 1,4-Dichlorobenzene | 147.76 | 140.50 | 140.80 | 6.96 | 4.71 |
| 1,3,5,7-Tetroxane | 142.22 | 140.80 |  | 1.42 | 1.00 |
| 4-Aminobutyric acid | 146.40 | 140.80 | 140.10 | 6.30 | 4.30 |
| Benzimidazole | 128.80 | 140.80 | 142.00 | -13.20 | -10.25 |
| 1H-Indazole | 128.80 | 140.80 | 145.30 | -16.50 | -12.81 |
| 3-Thiophenecarboxylic acid | 134.17 | 141.10 | 141.00 | -6.83 | -5.09 |
| 2-Acetylpyrrole | 132.05 | 141.30 | 144.40 | -12.35 | -9.35 |
| 2-Aminobutyric acid | 146.40 | 141.90 | 142.70 | 3.70 | 2.53 |
| 6-Aminopurine | 147.00 | 142.80 |  | 4.20 | 2.86 |
| Vitamin B3 | 151.30 | 143.50 | 141.40 | 9.90 | 6.54 |
| 2-Acetylfuran | 140.94 | 144.20 |  | -3.26 | -2.31 |
| Norbornene | 129.90 | 144.50 | 145.60 | -15.70 | -12.09 |
| N,N'-Dimethylurea | 133.30 | 144.70 | 148.90 | -15.60 | -11.70 |
| 2-Thiophenecarboxylic acid | 153.78 | 145.20 | 143.00 | 10.78 | 7.01 |
| cis-3-Chloro-2-butenoic acid | 140.20 | 145.30 |  | -5.10 | -3.64 |
| trans-3-Chloro-2-butenoic acid | 159.80 | 145.30 |  | 14.50 | 9.07 |
| N-Methylsuccinimide | 150.45 | 145.90 | 145.50 | 4.95 | 3.29 |
| 4-Chloroaniline | 151.20 | 146.00 | 147.20 | 4.00 | 2.65 |
| Fumaric acid | 141.80 | 146.00 | 146.00 | -4.20 | -2.96 |
| Maleic acid | 135.60 | 146.00 | 148.60 | -13.00 | -9.59 |
| Nicotinic acid | 151.30 | 146.70 | 145.50 | 5.80 | 3.83 |
| 1,4-Dibromobenzene | 145.70 | 146.80 | 144.30 | 1.40 | 0.96 |
| Diethanolamine | 137.00 | 147.40 |  | -10.40 | -7.59 |
| Thiobarbituric acid | 137.20 | 147.60 | 152.30 | -15.10 | -11.01 |
| Ethylene oxalate | 141.90 | 148.10 | 148.30 | -6.40 | -4.51 |
| Thianaphthene | 163.01 | 148.90 | 149.00 | 14.01 | 8.59 |
| Xanthine | 151.34 | 149.00 | 147.20 | 4.14 | 2.74 |
| Benzamide | 153.80 | 149.20 | 149.60 | 4.20 | 2.73 |
| p-Cresol | 150.20 | 149.50 | 149.30 | 0.90 | 0.60 |
| o-Cresol | 154.56 | 149.50 | 149.40 | 5.16 | 3.34 |
| 1,4-Dioxane-2,5-dione | 133.28 | 149.50 | 149.60 | -16.32 | -12.24 |
| 5-Methyluracil | 150.20 | 150.30 | 146.90 | 3.30 | 2.20 |
| N,N-Dimethylurea | 134.30 | 150.40 | 157.70 | -23.40 | -17.42 |
| Bicyclo[2.2.2]oct-2-ene | 156.73 | 150.90 | 151.60 | 5.13 | 3.27 |
| 1,2-Diaminobenzene | 150.82 | 151.40 | 148.20 | 2.62 | 1.74 |
| 1,3-Diaminobenzene | 153.70 | 151.40 | 148.20 | 5.50 | 3.58 |
| Threonine | 155.31 | 152.00 | 153.60 | 1.71 | 1.10 |
| Hexamethylenetetramine | 152.29 | 152.10 |  | 0.19 | 0.12 |
| Benzoic acid | 147.07 | 152.40 | 153.10 | -6.03 | -4.10 |
| N-Acetylglycinamide | 152.70 | 153.20 | 152.80 | -0.10 | -0.07 |
| Glycylglycine | 163.97 | 153.20 | 152.50 | 11.47 | 7.00 |
| Isoquinoline | 177.68 | 154.00 | 153.30 | 24.38 | 13.72 |
| 1,4-Cyclohexanedione | 161.50 | 154.30 | 154.60 | 6.90 | 4.27 |
| p-Toluidine | 165.20 | 155.30 | 153.70 | 11.50 | 6.96 |
| 4-Hydroxybenzaldehyde | 149.90 | 155.40 | 156.50 | -6.60 | -4.40 |
| Succindiamide | 158.17 | 156.50 | 155.60 | 2.57 | 1.62 |
| 2-Pyridinealdoxime | 151.93 | 156.60 |  | -4.67 | -3.07 |
| 1,3,5-Trichlorobenzene | 158.35 | 156.90 | 157.10 | 1.25 | 0.79 |
| 1,3-Dithiane sulfone | 157.60 | 157.70 |  | -0.10 | -0.06 |
| epsilon-Caprolactam | 156.78 | 157.90 | 157.50 | -0.72 | -0.46 |
| Guanine | 160.70 | 157.90 |  | 2.80 | 1.74 |
| 2-Benzimidazolethiol | 171.23 | 158.10 | 152.60 | 18.63 | 10.88 |
| Butylene carbonate | 139.34 | 158.10 | 163.70 | -24.36 | -17.48 |
| 2,2-Dimethyl-1,3-propanediol | 183.18 | 158.10 | 158.60 | 24.58 | 13.42 |
| Benzenecarbothioamide | 152.80 | 158.40 |  | -5.60 | -3.66 |
| Asparagine | 159.80 | 158.70 | 157.90 | 1.90 | 1.19 |
| 1,3-Benzoxazole-2-thione | 153.10 | 158.70 |  | -5.60 | -3.66 |
| Naphthalene | 168.20 | 159.80 | 159.30 | 8.90 | 5.29 |
| 5-Methylisoxazole-3-carboxylic acid | 146.20 | 159.80 | 164.70 | -18.50 | -12.65 |
| 2-Aminobenzothiazole | 153.20 | 160.40 | 164.30 | -11.10 | -7.25 |
| Isopropylcarbamide | 170.30 | 161.60 | 161.10 | 9.20 | 5.40 |
| 3-Acetyl-2-oxazolidinone | 164.70 | 161.70 | 161.70 | 3.00 | 1.82 |
| Pivalamide | 159.80 | 162.00 | 162.60 | -2.80 | -1.75 |
| Aspartic acid | 155.18 | 162.00 | 159.20 | -4.02 | -2.59 |
| Methyl methacrylate | 165.30 | 162.40 | 163.00 | 2.30 | 1.39 |
| Butanedioic acid | 153.60 | 163.00 | 162.10 | -8.50 | -5.53 |
| Norbornane | 151.00 | 163.30 | 163.90 | -12.90 | -8.54 |
| Bicyclo[2.2.2]octane | 157.69 | 163.60 | 164.00 | -6.31 | -4.00 |
| Erythritol | 161.90 | 164.20 | 163.00 | -1.10 | -0.68 |
| Phthalonitrile | 161.30 | 164.20 | 164.50 | -3.20 | -1.98 |
| 1,4-Benzenedicarbonitrile | 161.20 | 164.20 | 164.50 | -3.30 | -2.05 |
| 1-Azabicyclo[2.2.2]octane | 169.37 | 164.20 | 164.30 | 5.07 | 2.99 |
| DABCO | 153.18 | 164.90 | 167.00 | -13.82 | -9.02 |
| 4-Nitrophenol | 143.90 | 165.00 | 166.20 | -22.30 | -15.50 |
| Trimethylacetic acid | 177.80 | 165.20 | 165.10 | 12.70 | 7.14 |
| Valine | 165.00 | 165.40 | 165.40 | -0.40 | -0.24 |
| 3-Chloronitrobenzene | 168.20 | 165.40 | 165.50 | 2.70 | 1.61 |
| 4-Chloronitrobenzene | 169.90 | 165.40 | 164.70 | 5.20 | 3.06 |
| 2,5-Dihydroxytoluene | 174.90 | 165.50 | 164.30 | 10.60 | 6.06 |
| Phthalic anhydride | 160.00 | 165.70 | 167.10 | -7.10 | -4.44 |
| Benzenesulfonamide | 172.91 | 165.90 | 165.30 | 7.61 | 4.40 |
| 5-Aminovaleric acid | 163.70 | 166.20 | 166.60 | -2.90 | -1.77 |
| 1,3-Benzothiazole-2-thione | 157.30 | 166.20 |  | -8.90 | -5.66 |
| 1,3,5-Tribromobenzene | 161.70 | 166.30 | 162.90 | -1.20 | -0.74 |
| 3-Thiopheneacetic acid | 167.76 | 166.50 | 167.50 | 0.26 | 0.15 |
| 2-Amino-2-methyl-1,3-propanediol | 161.00 | 166.70 | 169.40 | -8.40 | -5.22 |
| Uric acid | 166.10 | 167.00 | 170.20 | -4.10 | -2.47 |
| 2-Methylindole | 176.09 | 167.50 | 167.50 | 8.59 | 4.88 |
| trans-1,2-Cyclohexanediol | 163.20 | 167.80 | 168.50 | -5.30 | -3.25 |
| cis-1,2-Cyclohexanediol | 160.40 | 167.80 | 168.60 | -8.20 | -5.11 |
| Benzimidazole-2-thione | 171.23 | 167.90 | 168.30 | 2.93 | 1.71 |
| 3-Carboxyphenol | 157.30 | 168.40 | 168.00 | -10.70 | -6.80 |
| 4-Carboxyphenol | 155.20 | 168.40 | 168.70 | -13.50 | -8.70 |
| 4-Chlorobenzoic acid | 167.80 | 168.80 | 168.20 | -0.40 | -0.24 |
| 3-Chlorobenzoic acid | 163.60 | 168.80 | 169.80 | -6.20 | -3.79 |
| 2-Chlorobenzoic acid | 163.20 | 168.80 | 169.80 | -6.60 | -4.04 |
| 1,4-Diiodobenzene | 160.77 | 168.80 |  | -8.03 | -4.99 |
| 1,2-Diiodobenzene | 190.00 | 168.80 |  | 21.20 | 11.16 |
| Salicylic acid | 160.90 | 169.60 | 169.60 | -8.70 | -5.41 |
| FOX-7 | 176.59 | 170.00 | 165.30 | 11.29 | 6.39 |
| 1-Benzopyran-4-one | 176.80 | 170.30 |  | 6.50 | 3.68 |
| 2-Thiopheneacetic acid | 159.23 | 170.60 | 171.90 | -12.67 | -7.96 |
| 3-Nitroaniline | 158.84 | 170.80 | 170.80 | -11.96 | -7.53 |
| 4-Nitroaniline | 154.20 | 170.80 | 173.20 | -19.00 | -12.32 |
| Acenaphthylene | 183.59 | 170.90 | 171.40 | 12.19 | 6.64 |
| 3,4-Dihydroxybenzaldehyde | 205.90 | 171.50 | 167.90 | 38.00 | 18.46 |
| 2-Bromobenzoic acid | 154.00 | 171.90 | 170.60 | -16.60 | -10.78 |
| 3-Bromobenzoic acid | 151.40 | 171.90 | 172.40 | -21.00 | -13.87 |
| 4-Bromobenzoic acid | 151.40 | 171.90 | 172.80 | -21.40 | -14.13 |
| 2-Nitroaniline | 166.00 | 172.00 | 172.10 | -6.10 | -3.67 |
| 2,4-Dichlorobenzaldehyde | 186.50 | 172.20 | 172.30 | 14.20 | 7.61 |
| 1,2,4,5-Tetrachlorobenzene | 202.50 | 173.30 | 171.00 | 31.50 | 15.56 |
| 2-Amino-5-nitropyridine | 158.00 | 173.60 | 184.60 | -26.60 | -16.84 |
| p-Anthranilic acid | 177.80 | 174.20 | 174.70 | 3.10 | 1.74 |
| m-Anthranilic acid | 162.80 | 174.20 | 174.30 | -11.50 | -7.06 |
| TRIS | 171.27 | 174.30 | 174.30 | -3.03 | -1.77 |
| Pentaerythritol | 188.40 | 174.60 | 174.00 | 14.40 | 7.64 |
| 4-Nitrotoluene | 172.30 | 174.70 | 176.40 | -4.10 | -2.38 |
| 4-Ethylphenol | 206.90 | 175.00 | 174.50 | 32.40 | 15.66 |
| o-Anthranilic acid | 165.30 | 175.40 | 176.70 | -11.40 | -6.90 |
| 8-Quinolinol | 180.42 | 175.50 | 176.60 | 3.82 | 2.12 |
| 2-Naphthol | 172.80 | 175.80 | 176.60 | -3.80 | -2.20 |
| 1-Naphthol | 166.90 | 175.80 | 176.60 | -9.70 | -5.81 |
| 1,3,5,7,9-Pentoxecane | 171.50 | 176.00 |  | -4.50 | -2.62 |
| N-Methylphthalimide | 185.33 | 177.10 | 179.50 | 5.83 | 3.15 |
| 2,6-Pyridinedicarboxylic acid | 166.31 | 177.50 | 179.60 | -13.29 | -7.99 |
| 2-Methylbenzoic acid | 174.90 | 178.10 | 178.30 | -3.40 | -1.94 |
| 4-Methylbenzoic acid | 169.00 | 178.10 | 178.40 | -9.40 | -5.56 |
| 3-Methylbenzoic acid | 163.60 | 178.10 | 178.10 | -14.50 | -8.86 |
| Acetanilide | 179.30 | 178.80 | 179.60 | -0.30 | -0.17 |
| 1,3-Dimethyluracil | 170.00 | 178.80 | 181.90 | -11.90 | -7.00 |
| Alanylglycine | 168.00 | 179.70 | 180.50 | -12.50 | -7.44 |
| 5-Amino-2-mercaptobenzimidazole | 172.00 | 179.90 | 183.30 | -11.30 | -6.57 |
| Benzoylformic acid | 192.90 | 180.10 | 180.20 | 12.70 | 6.58 |
| N-Acetyl-L-alanine amide | 181.30 | 180.60 | 177.70 | 3.60 | 1.99 |
| 4,4'-Bipyridine | 185.60 | 181.30 | 184.10 | 1.50 | 0.81 |
| exo-Dicyclopentadiene | 188.70 | 181.80 | 179.80 | 8.90 | 4.72 |
| Enantholactam | 205.00 | 181.90 | 181.50 | 23.50 | 11.46 |
| N,N'-Diethylurea | 219.00 | 181.90 | 176.60 | 42.40 | 19.36 |
| Glutaramide | 170.21 | 182.00 | 182.20 | -11.99 | -7.04 |
| 2-(Acetylamino)-N-methylethanamide | 177.75 | 182.20 | 182.10 | -4.35 | -2.45 |
| 3-Phenylpropiolic acid | 180.00 | 182.30 |  | -2.30 | -1.28 |
| 2,4,6-Tribromophenol | 172.00 | 182.40 | 185.30 | -13.30 | -7.73 |
| Glutamine | 184.18 | 183.00 | 183.50 | 0.68 | 0.37 |
| Adamantane | 190.00 | 183.10 | 182.60 | 7.40 | 3.89 |
| Benzofuran-2-carboxylic acid | 184.80 | 184.10 | 183.70 | 1.10 | 0.60 |
| Indole-2-carboxylic acid | 178.40 | 184.30 | 184.50 | -6.10 | -3.42 |
| Hexachloroethane | 198.24 | 184.60 |  | 13.64 | 6.88 |
| n-Butylurea | 207.90 | 185.20 | 186.90 | 21.00 | 10.10 |
| 2-Methylnaphthalene | 195.80 | 185.50 | 185.80 | 10.00 | 5.11 |
| 3,5-Dimethylisoxazole-4-carboxylic acid | 177.80 | 185.80 | 185.30 | -7.50 | -4.22 |
| 2-Amino-4-methylbenzothiazole | 182.30 | 186.10 | 188.10 | -5.80 | -3.18 |
| 2-Amino-6-methylbenzothiazole | 197.08 | 186.10 | 180.70 | 16.38 | 8.31 |
| Glutamic acid | 175.06 | 186.20 | 186.80 | -11.74 | -6.71 |
| trans-Cinnamic acid | 197.50 | 186.30 | 185.80 | 11.70 | 5.92 |
| 4-Amino-3-furazanecarboxamidoxime | 180.40 | 186.40 |  | -6.00 | -3.33 |
| N-Isobutylacetamide | 190.00 | 186.60 | 186.50 | 3.50 | 1.84 |
| 2-Methylbutanedioic acid | 199.60 | 186.60 | 185.60 | 14.00 | 7.01 |
| sec-Butylurea | 207.90 | 187.10 | 183.60 | 24.30 | 11.69 |
| N,N-Diethylurea | 183.30 | 187.50 | 188.40 | -5.10 | -2.78 |
| 4-Dimethylaminopyridine | 181.32 | 187.50 | 190.70 | -9.38 | -5.17 |
| 4-Aminobenzenesulfonamide | 199.75 | 187.70 | 186.50 | 13.25 | 6.63 |
| 2-Aminobenzenesulfonamide | 173.92 | 187.70 | 191.10 | -17.18 | -9.88 |
| Hexamethyleneglycol | 190.00 | 187.90 | 187.10 | 2.90 | 1.53 |
| t-Butylcarbamide | 185.50 | 187.90 | 191.10 | -5.60 | -3.02 |
| Mandelic acid | 199.20 | 188.00 | 187.10 | 12.10 | 6.07 |
| 2,4,6-Tribromoaniline | 181.40 | 188.10 | 183.90 | -2.50 | -1.38 |
| Pentanedioic acid | 186.90 | 188.40 | 189.60 | -2.70 | -1.44 |
| Cyclohexanone oxime | 199.38 | 188.60 |  | 10.78 | 5.41 |
| 2-(Dinitromethylene)-1,3-diazacyclopentane | 205.41 | 188.60 | 182.90 | 22.51 | 10.96 |
| 1-Norbornyl cyanide | 172.90 | 188.90 | 191.80 | -18.90 | -10.93 |
| Tartaric acid | 184.50 | 189.10 | 190.90 | -6.40 | -3.47 |
| endo-2-Cyanonorbornane | 193.93 | 189.40 | 189.20 | 4.73 | 2.44 |
| exo-Bicyclo[2.2.1]heptane-2-carbonitrile | 200.00 | 189.40 | 189.80 | 10.20 | 5.10 |
| Pentafluorophenol | 201.30 | 189.40 | 188.10 | 13.20 | 6.56 |
| 3,4,5,6-Tetrahydrophthalic anhydride | 193.39 | 189.80 | 189.80 | 3.59 | 1.86 |
| 1,2-Dinitrobenzene | 195.00 | 190.20 | 192.00 | 3.00 | 1.54 |
| 1,4-Dinitrobenzene | 192.00 | 190.20 | 188.00 | 4.00 | 2.08 |
| 1,3-Dinitrobenzene | 188.30 | 190.20 | 193.90 | -5.60 | -2.97 |
| Acenaphthene | 191.38 | 190.70 | 190.70 | 0.68 | 0.36 |
| Isoleucine | 188.28 | 190.90 | 189.90 | -1.62 | -0.86 |
| Leucine | 200.80 | 190.90 | 190.20 | 10.60 | 5.28 |
| N-Methylpivalamide | 183.00 | 191.00 | 192.00 | -9.00 | -4.92 |
| t-Butylacetamide | 190.00 | 191.10 | 190.50 | -0.50 | -0.26 |
| 4-Nitrophthalimide | 199.62 | 191.10 | 189.30 | 10.32 | 5.17 |
| 1-Benzylimidazole | 182.10 | 191.10 | 193.40 | -11.30 | -6.21 |
| 3-Nitrophthalimide | 222.29 | 191.10 | 190.00 | 32.29 | 14.53 |
| Ethriol | 213.80 | 191.20 | 189.80 | 24.00 | 11.23 |
| 2,5-Dibromobenzoic acid | 223.00 | 191.40 | 188.90 | 34.10 | 15.29 |
| 3,5-Dibromobenzoic acid | 223.10 | 191.40 | 188.10 | 35.00 | 15.69 |
| 2-Methylbenzenesulfonamide | 193.48 | 191.60 | 194.30 | -0.82 | -0.42 |
| 4-Methylbenzenesulfonamide | 205.46 | 191.60 | 189.40 | 16.06 | 7.82 |
| epsilon-Aminocaproic acid | 175.70 | 191.70 | 192.90 | -17.20 | -9.79 |
| Methyl 3-hydroxybenzoate | 205.00 | 192.00 | 191.50 | 13.50 | 6.59 |
| 4-Methoxybenzoic acid | 207.50 | 192.00 | 191.80 | 15.70 | 7.57 |
| Methyl 4-hydroxybenzoate | 214.41 | 192.00 | 191.90 | 22.51 | 10.50 |
| Arabinose | 184.10 | 192.10 | 192.20 | -8.10 | -4.40 |
| Xylose | 180.40 | 192.10 | 193.60 | -13.20 | -7.32 |
| Biphenyl | 198.40 | 192.70 | 192.80 | 5.60 | 2.82 |
| 2-Acetylbenzofuran | 192.10 | 192.90 | 193.10 | -1.00 | -0.52 |
| Dimethyl fumarate | 199.20 | 193.10 | 193.60 | 5.60 | 2.81 |
| 2-Adamantanol | 207.20 | 193.20 | 190.80 | 16.40 | 7.92 |
| 1,4-Phenylene diisocyanate | 211.70 | 193.20 |  | 18.50 | 8.74 |
| Dibenzofuran | 199.01 | 193.50 | 195.00 | 4.01 | 2.01 |
| 4-Nitrobenzoic acid | 180.30 | 193.60 | 193.90 | -13.60 | -7.54 |
| 3-Nitrobenzoic acid | 179.90 | 193.60 | 193.60 | -13.70 | -7.62 |
| Thiophenedicarboxylic acid | 204.87 | 194.10 | 178.20 | 26.67 | 13.02 |
| Ornithine | 191.20 | 194.30 | 193.90 | -2.70 | -1.41 |
| 2-Nitrobenzoic acid | 191.60 | 194.80 | 195.50 | -3.90 | -2.04 |
| 2-Hydroxyacetanilide | 182.40 | 194.80 | 196.60 | -14.20 | -7.79 |
| 4-Hydroxy-3-methoxybenzaldehyde | 187.60 | 195.00 | 194.80 | -7.20 | -3.84 |
| D-Ribose | 185.97 | 195.00 | 196.20 | -10.23 | -5.50 |
| 1-Adamantanol | 196.70 | 195.40 | 195.30 | 1.40 | 0.71 |
| Carbazole | 194.00 | 195.60 | 195.00 | -1.00 | -0.52 |
| Methyl N-phenylurethane | 203.30 | 196.10 | 193.30 | 10.00 | 4.92 |
| N-Phenylglycine | 177.40 | 196.10 | 197.40 | -20.00 | -11.27 |
| Chloranil | 193.80 | 196.20 |  | -2.40 | -1.24 |
| 1,3,5-Trichlorotrifluorobenzene | 197.00 | 196.30 | 196.50 | 0.50 | 0.25 |
| 2-Methyl-4-nitroaniline | 217.10 | 196.50 | 196.70 | 20.40 | 9.40 |
| 2-Ethyl-4-pyridinecarbothioamide | 201.30 | 196.90 |  | 4.40 | 2.19 |
| Terephthalic acid | 199.60 | 197.00 | 197.50 | 2.10 | 1.05 |
| Isophthalic acid | 201.70 | 197.00 | 196.80 | 4.90 | 2.43 |
| Phthalic acid | 188.11 | 197.00 | 197.60 | -9.49 | -5.04 |
| Fluorene | 203.13 | 197.50 | 197.30 | 5.83 | 2.87 |
| 2-Mercapto-5-methoxybenzimidazole | 195.40 | 197.70 | 200.30 | -4.90 | -2.51 |
| 4-Methyl-2-nitroaniline | 205.40 | 197.70 | 199.10 | 6.30 | 3.07 |
| Methyl 4-aminobenzoate | 200.00 | 197.80 | 198.70 | 1.30 | 0.65 |
| Thionaphthene-2-carboxylic acid | 179.00 | 197.80 | 201.70 | -22.70 | -12.68 |
| 1,6-Anhydro-beta-D-glucopyranose | 187.20 | 197.90 | 199.10 | -11.90 | -6.36 |
| Lactide | 184.30 | 198.10 | 198.10 | -13.80 | -7.49 |
| cis-Cyclohexane-1,2-dicarboxylic anhydride | 207.40 | 199.90 | 200.20 | 7.20 | 3.47 |
| endo-5-Norbornene-2,3-dicarboxylic anhydride | 183.30 | 200.30 | 202.20 | -18.90 | -10.31 |
| trans-Decahydroquinoline | 193.79 | 200.40 |  | -6.61 | -3.41 |
| Gallic acid | 199.55 | 200.50 | 199.70 | -0.15 | -0.08 |
| Mesitol | 189.60 | 200.90 | 200.60 | -11.00 | -5.80 |
| 2-Chloro-6-(trichloromethyl)pyridine | 189.35 | 200.90 |  | -11.55 | -6.10 |
| Phenylalanine | 203.10 | 201.10 | 201.10 | 2.00 | 0.98 |
| Dibenzo[b,d]thiophene | 223.73 | 201.60 | 198.80 | 24.93 | 11.14 |
| Methyl 4-methylbenzoate | 199.10 | 201.70 | 201.80 | -2.70 | -1.36 |
| Tyramine | 213.75 | 201.90 | 197.10 | 16.65 | 7.79 |
| trans-4-Coumaric acid | 193.00 | 202.30 | 201.50 | -8.50 | -4.40 |
| Arabitol | 201.35 | 203.20 | 204.40 | -3.05 | -1.51 |
| 3-Chloromandelic acid | 211.10 | 204.40 | 203.30 | 7.80 | 3.69 |
| N-(2-Cyanoethyl)aniline | 174.60 | 205.10 | 205.10 | -30.50 | -17.47 |
| Pentachlorophenol | 201.96 | 205.70 | 204.70 | -2.74 | -1.36 |
| beta-Alanyl-beta-alanine | 196.00 | 206.00 | 204.00 | -8.00 | -4.08 |
| Hexachlorobenzene | 201.29 | 206.10 | 205.10 | -3.81 | -1.89 |
| Xylitol | 207.00 | 206.80 | 206.00 | 1.00 | 0.48 |
| 3-Nitrophthalic anhydride | 224.02 | 206.90 | 205.60 | 18.42 | 8.22 |
| 4-Nitrophthalic anhydride | 175.74 | 206.90 | 209.20 | -33.46 | -19.04 |
| Alanylalanine | 194.90 | 207.00 | 207.60 | -12.70 | -6.52 |
| 3-Nitrobenzenesulfonamide | 199.97 | 207.10 | 211.80 | -11.83 | -5.92 |
| 4-Nitrobenzenesulfonamide | 219.98 | 207.10 | 204.60 | 15.38 | 6.99 |
| (L)-Methionine | 205.16 | 207.40 |  | -2.24 | -1.09 |
| Adipamide | 203.50 | 207.40 | 207.30 | -3.80 | -1.87 |
| N-Acetyl-L-prolinamide | 208.90 | 207.60 | 212.60 | -3.70 | -1.77 |
| Theophylline | 196.80 | 207.80 | 212.40 | -15.60 | -7.93 |
| 2-Chloro-5-(trichloromethyl)pyridine | 192.00 | 207.80 |  | -15.80 | -8.23 |
| 3-Aminocinnamic acid | 227.60 | 208.10 | 207.80 | 19.80 | 8.70 |
| 2-Nitrobenzenesulfonamide | 179.54 | 208.30 | 212.50 | -32.96 | -18.36 |
| 1-Cyanoadamantane | 218.60 | 208.60 | 208.40 | 10.20 | 4.67 |
| 2-Hydroxybiphenyl | 227.61 | 208.70 | 208.50 | 19.11 | 8.40 |
| 2-Chlorobiphenyl | 211.92 | 209.10 | 208.90 | 3.02 | 1.43 |
| 4-Chlorobiphenyl | 243.76 | 209.10 | 209.00 | 34.76 | 14.26 |
| 2-(Acetylamino)-N-methylpropanamide | 201.30 | 209.60 | 212.10 | -10.80 | -5.37 |
| Trimellitic anhydride | 248.90 | 210.30 | 208.30 | 40.60 | 16.31 |
| 2-Deoxy-d-glucose | 200.20 | 210.40 | 210.00 | -9.80 | -4.90 |
| 2-(Dinitromethylene)-1,3-diazacyclohexane | 207.76 | 210.60 | 210.80 | -3.04 | -1.46 |
| 1,2,4,5-Tetramethylbenzene | 204.02 | 210.60 | 211.40 | -7.38 | -3.62 |
| 1,10-Phenanthroline | 198.74 | 211.10 | 213.10 | -14.36 | -7.23 |
| Guajen | 216.47 | 211.20 | 211.70 | 4.77 | 2.20 |
| 2,6-Dimethylnaphthalene | 204.20 | 211.20 | 211.10 | -6.90 | -3.38 |
| 2,7-Dimethylnaphthalene | 204.39 | 211.20 | 211.70 | -7.31 | -3.58 |
| 1,8-Dimethylnaphthalene | 242.80 | 211.20 | 211.10 | 31.70 | 13.06 |
| Benzo[h]quinoline | 206.36 | 211.40 | 211.80 | -5.44 | -2.64 |
| Phenanthridine | 201.87 | 211.40 | 212.00 | -10.13 | -5.02 |
| Anthracene | 210.50 | 211.80 | 212.10 | -1.60 | -0.76 |
| Phenanthrene | 220.30 | 211.80 | 212.10 | 8.20 | 3.72 |
| DAAF | 215.53 | 212.50 |  | 3.03 | 1.41 |
| Dibenzo-p-dioxin | 206.70 | 212.70 | 214.60 | -7.90 | -3.82 |
| 4-Methoxycinnamaldehyde | 217.40 | 212.90 | 209.80 | 7.60 | 3.50 |
| 2,4,6-Trimethylbenzonitrile | 220.50 | 213.20 | 211.40 | 9.10 | 4.13 |
| 4-Methyl-4-cyclohexene-1,2-dicarboxylic anhydride | 203.51 | 213.20 | 213.60 | -10.09 | -4.96 |
| (1,1-Dimethylethyl)urea | 185.50 | 213.30 | 216.70 | -31.20 | -16.82 |
| 3-Cyano-4-methoxymethyl-6-methyl-(2-pyridone) | 211.76 | 213.50 | 218.20 | -6.44 | -3.04 |
| 1,6-Hexanedioic acid | 196.60 | 213.90 | 213.80 | -17.20 | -8.75 |
| Diphenyl ether | 216.56 | 214.10 | 213.80 | 2.76 | 1.27 |
| 2-Phenylaniline | 221.86 | 214.50 | 215.70 | 6.16 | 2.78 |
| DNTA hydrate | 213.13 | 215.40 | 212.70 | 0.43 | 0.20 |
| Hippuric acid | 217.00 | 215.70 | 216.20 | 0.80 | 0.37 |
| 4-Methyl-2-(dinitromethylene)-1,3-diazacyclopentane | 218.89 | 215.90 | 221.70 | -2.81 | -1.28 |
| Dibenzo[b,e]pyridine | 205.07 | 216.80 | 218.90 | -13.83 | -6.74 |
| Trimethylhydroquinone | 217.60 | 217.00 | 217.80 | -0.20 | -0.09 |
| Tyrosine | 216.44 | 217.10 | 216.90 | -0.46 | -0.21 |
| Diphenylmethane | 233.50 | 218.20 | 218.00 | 15.50 | 6.64 |
| Caffeic acid | 181.96 | 218.30 | 222.00 | -40.04 | -22.00 |
| 4-Phenyltoluene | 238.74 | 218.40 | 217.90 | 20.84 | 8.73 |
| 3,4-Dimethoxybenzaldehyde | 213.50 | 218.60 | 218.00 | -4.50 | -2.11 |
| 4,4'-Difluorobiphenyl | 222.77 | 219.00 | 218.60 | 4.17 | 1.87 |
| Xanthone | 226.00 | 219.00 | 219.20 | 6.80 | 3.01 |
| N-Propylpiperidine | 202.30 | 219.20 | 220.10 | -17.80 | -8.80 |
| 9,10-Dihydrophenanthrene | 243.80 | 219.40 | 218.80 | 25.00 | 10.25 |
| 4,5-Dihydroxy-2-(dinitromethylene)imidazolidine | 217.76 | 219.60 | 226.30 | -8.54 | -3.92 |
| 4-Nitroacetanilide | 230.50 | 220.00 | 219.10 | 11.40 | 4.95 |
| Benzophenone | 224.80 | 220.40 | 220.10 | 4.70 | 2.09 |
| 1,2,4,5-Benzenetetracarbonitrile | 222.30 | 220.70 | 216.80 | 5.50 | 2.47 |
| 9,10-Dihydroanthracene | 219.06 | 220.80 | 222.20 | -3.14 | -1.43 |
| 2-Phenylbenzimidazole | 208.09 | 221.70 | 223.50 | -15.41 | -7.41 |
| 1-Adamantylcarboxamide | 220.50 | 221.80 | 221.70 | -1.20 | -0.54 |
| Fluoranthene | 230.25 | 221.90 | 222.00 | 8.25 | 3.58 |
| Phenazine | 234.30 | 221.90 | 205.90 | 28.40 | 12.12 |
| Diamantane | 223.40 | 222.10 | 221.50 | 1.90 | 0.85 |
| 4-Nitrophenetole | 246.00 | 222.30 | 220.00 | 26.00 | 10.57 |
| Tolane | 225.90 | 222.60 |  | 3.30 | 1.46 |
| N-Methylcarbazole | 217.84 | 222.80 | 224.50 | -6.66 | -3.06 |
| 5-Methyl-5-phenylhydantoin | 235.85 | 222.80 | 225.80 | 10.05 | 4.26 |
| Phenoxathiin | 213.77 | 223.00 | 234.40 | -20.63 | -9.65 |
| beta-D-Fructose | 227.70 | 223.10 | 227.00 | 0.70 | 0.31 |
| D-Tagatose | 228.00 | 223.10 | 223.20 | 4.80 | 2.11 |
| Inositol | 218.00 | 223.40 | 226.90 | -8.90 | -4.08 |
| Pyromellitic dianhydride | 219.90 | 223.70 | 227.00 | -7.10 | -3.23 |
| D-Mannose | 223.22 | 224.00 | 225.00 | -1.78 | -0.80 |
| alpha-D-Glucose | 219.19 | 224.00 | 225.30 | -6.11 | -2.79 |
| D-Galactose | 213.70 | 224.00 | 225.90 | -12.20 | -5.71 |
| D-Psicose | 221.30 | 224.20 | 225.40 | -4.10 | -1.85 |
| Diphenyl sulfide | 239.70 | 224.40 | 224.00 | 15.70 | 6.55 |
| Pentaerythritol tetrabromide | 218.57 | 224.70 |  | -6.13 | -2.80 |
| 4,4'-Dihydroxybiphenyl | 224.31 | 224.80 | 225.40 | -1.09 | -0.49 |
| 4-Dimethylaminobenzaldehyde | 234.00 | 224.90 | 215.10 | 18.90 | 8.08 |
| 9,10-Anthraquinone | 226.60 | 225.30 | 224.70 | 1.90 | 0.84 |
| Ethyl 4-hydroxybenzoate | 233.93 | 225.70 | 225.00 | 8.93 | 3.82 |
| Ethyl 3-hydroxybenzoate | 217.22 | 225.70 | 226.20 | -8.98 | -4.13 |
| N-Benzyl-beta-alanine | 221.70 | 226.00 |  | -4.30 | -1.94 |
| 1,4-Anthraquinone | 223.34 | 226.20 | 226.90 | -3.56 | -1.59 |
| trans-Stilbene | 235.00 | 226.60 | 226.70 | 8.30 | 3.53 |
| 2,3,5,6-Tetramethyl-1,4-benzoquinone | 231.54 | 226.90 | 222.50 | 9.04 | 3.90 |
| Cyclohexylbenzene | 263.17 | 226.90 | 227.90 | 35.27 | 13.40 |
| N-Benzylalanine | 222.40 | 227.00 | 228.90 | -6.50 | -2.92 |
| Citrulline | 232.60 | 227.30 | 226.80 | 5.80 | 2.49 |
| 4-Nitrocinnamic acid | 238.10 | 227.50 | 227.00 | 11.10 | 4.66 |
| 3-Nitrocinnamic acid | 240.20 | 227.50 | 227.70 | 12.50 | 5.20 |
| 2-Nitrocinnamic acid | 240.60 | 227.50 | 227.40 | 13.20 | 5.49 |
| Pyrene | 229.70 | 227.80 | 227.90 | 1.80 | 0.78 |
| 1,3-Diethylbarbituric acid | 248.50 | 227.80 | 222.10 | 26.40 | 10.62 |
| Diphenylmethanol | 236.80 | 228.30 | 228.00 | 8.80 | 3.72 |
| Ethyl vanillin | 212.20 | 228.80 | 230.40 | -18.20 | -8.58 |
| N-Acetyl valinamide | 232.10 | 229.60 | 229.10 | 3.00 | 1.29 |
| Ethyl N-phenylcarbamate | 227.20 | 229.80 | 226.80 | 0.40 | 0.18 |
| Camphene | 235.13 | 230.90 | 230.10 | 5.03 | 2.14 |
| Ethyl 4-aminobenzoate | 236.40 | 231.50 | 232.20 | 4.20 | 1.78 |
| 1,3,5-Trinitrobenzene | 222.70 | 231.50 | 237.20 | -14.50 | -6.51 |
| 2-Chloro-N,N-dimethylnicotinamide | 259.40 | 232.00 |  | 27.40 | 10.56 |
| Pimelamide | 230.82 | 232.90 | 231.90 | -1.08 | -0.47 |
| Caffeine | 232.00 | 232.90 | 230.50 | 1.50 | 0.65 |
| trans-Azobenzene | 229.00 | 233.10 |  | -4.10 | -1.79 |
| Dopa | 260.60 | 233.20 | 231.30 | 29.30 | 11.24 |
| Thianthrene | 224.97 | 233.30 | 255.80 | -30.83 | -13.70 |
| Barbital | 228.70 | 233.80 | 235.70 | -7.00 | -3.06 |
| 1-Acetyladamantane | 217.50 | 233.80 | 235.50 | -18.00 | -8.28 |
| 1,2,3-Trichloro-4,5,6-trimethylbenzene | 252.34 | 234.10 | 232.80 | 19.54 | 7.74 |
| Tetrahydroanthracene | 247.02 | 234.40 | 234.90 | 12.12 | 4.91 |
| 2-(Dinitromethylene)-1,3-diazacycloheptane | 215.40 | 234.60 | 241.60 | -26.20 | -12.16 |
| 3,4-Dihydroxyhydrocinnamic acid | 218.62 | 235.30 | 234.60 | -15.98 | -7.31 |
| Bicyclo[3.3.3]undecane | 213.20 | 235.60 | 237.10 | -23.90 | -11.21 |
| Camphor | 242.70 | 235.90 | 235.30 | 7.40 | 3.05 |
| Pentamethylbenzene | 210.50 | 236.40 | 238.00 | -27.50 | -13.06 |
| Tryptophane | 238.15 | 237.40 | 235.80 | 2.35 | 0.99 |
| 4-Methylphenanthrene | 262.80 | 237.50 | 237.30 | 25.50 | 9.70 |
| 4-Nitrophthalic acid | 232.24 | 238.20 | 237.80 | -5.56 | -2.39 |
| 3-Nitrophthalic acid | 247.02 | 238.20 | 237.00 | 10.02 | 4.06 |
| 1,8-Octanediol | 236.36 | 238.80 | 239.40 | -3.04 | -1.29 |
| 1,3-Diethyl-2-thiobarbituric acid | 236.90 | 239.10 | 236.20 | 0.70 | 0.30 |
| Heptanedioic acid | 240.70 | 239.30 | 239.30 | 1.40 | 0.58 |
| 3,4-Dimethoxyphenylacetonitrile | 243.50 | 240.60 | 244.00 | -0.50 | -0.21 |
| 9-Ethyl-9H-carbazole | 238.20 | 241.40 | 239.30 | -1.10 | -0.46 |
| 2,4,6-Trimethylphenyl isocyanide | 241.80 | 241.80 |  | 0.00 | 0.00 |
| 1,2,3,4-Tetrahydro-N-methylcarbazole | 241.35 | 241.80 | 243.20 | -1.85 | -0.77 |
| Ferulic acid | 235.94 | 241.90 | 243.10 | -7.16 | -3.03 |
| 1,8-Dinitronaphthalene | 254.40 | 242.20 | 240.20 | 14.20 | 5.58 |
| 1,5-Dinitronaphthalene | 259.40 | 242.20 | 241.20 | 18.20 | 7.02 |
| Sorbitol | 239.00 | 242.30 | 239.70 | -0.70 | -0.29 |
| 8-Aminocaprylic acid | 251.70 | 242.60 | 243.80 | 7.90 | 3.14 |
| 1-Methoxy-a-D-glucopyranoside | 233.70 | 242.80 | 242.80 | -9.10 | -3.89 |
| Alanylglycylglycine | 236.00 | 243.00 | 241.70 | -5.70 | -2.42 |
| Isoborneol | 261.06 | 243.10 | 241.90 | 19.16 | 7.34 |
| Borneol | 261.06 | 243.10 | 238.80 | 22.26 | 8.53 |
| 3-Fluoro-5-(3-pyridinyloxy)-benzenamine | 216.03 | 243.30 | 246.10 | -30.07 | -13.92 |
| 9-Hydroxy-1,4-anthraquinone | 237.67 | 243.40 | 242.00 | -4.33 | -1.82 |
| 1,2-Diphenylethane | 253.76 | 243.60 | 242.80 | 10.96 | 4.32 |
| 4,4'-Dimethylbiphenyl | 242.26 | 244.20 | 244.50 | -2.24 | -0.92 |
| Dimethylterephthalate | 261.10 | 244.20 | 241.90 | 19.20 | 7.35 |
| Citric acid | 226.40 | 244.40 | 251.30 | -24.90 | -11.00 |
| N-Butylpiperidine | 242.97 | 244.70 | 246.20 | -3.23 | -1.33 |
| 2-Benzylbenzimidazole | 228.92 | 245.00 | 246.80 | -17.88 | -7.81 |
| Naphthalene-1,5-diisocyanate | 223.40 | 245.20 |  | -21.80 | -9.76 |
| Glyceryl-2-benzoate | 236.40 | 245.80 | 247.70 | -11.30 | -4.78 |
| Dulcose | 238.50 | 245.90 | 247.60 | -9.10 | -3.82 |
| 4,5,9,10-Tetrahydropyrene | 257.12 | 246.00 | 243.40 | 13.72 | 5.34 |
| Benzil | 245.70 | 248.10 | 248.60 | -2.90 | -1.18 |
| Diphenyldisulfide | 241.70 | 248.30 |  | -6.60 | -2.73 |
| 1-Methoxycarbonyladamantane | 264.20 | 248.60 | 247.60 | 16.60 | 6.28 |
| 2,9-Dimethyl-1,10-phenanthroline | 253.00 | 248.70 | 246.30 | 6.70 | 2.65 |
| 2,4,6-Trinitrophenol | 239.70 | 248.70 | 250.90 | -11.20 | -4.67 |
| Diphenylcarbonate | 263.13 | 248.70 | 238.80 | 24.33 | 9.25 |
| 2,6-Naphthalenedicarboxylic acid | 230.30 | 249.00 | 249.10 | -18.80 | -8.16 |
| Diphenylsulfone | 244.30 | 250.00 | 252.40 | -8.10 | -3.32 |
| Bisphenol F | 246.30 | 250.20 | 250.30 | -4.00 | -1.62 |
| Menthol | 250.10 | 250.40 | 251.90 | -1.80 | -0.72 |
| Pentamethylchlorobenzene | 244.80 | 252.70 | 253.90 | -9.10 | -3.72 |
| 1-Benzoylglycerol | 238.90 | 252.90 | 252.70 | -13.80 | -5.78 |
| N-Acetyl-D-leucine amide | 259.60 | 255.00 | 255.20 | 4.40 | 1.69 |
| 2,3,4,5-Tetramethylbenzoic acid | 249.50 | 255.30 | 255.10 | -5.60 | -2.24 |
| 1,4-Bis-(2-hydroxyethyl)piperazine | 250.68 | 255.30 | 259.60 | -8.92 | -3.56 |
| Propyl phenylcarbamate | 263.60 | 255.30 | 254.40 | 9.20 | 3.49 |
| 2,3,5,6-Tetramethylbenzoic acid | 231.70 | 255.30 | 256.20 | -24.50 | -10.57 |
| 5-Cyano-5H-dibenzo[a,d]cycloheptene | 259.80 | 255.40 | 255.60 | 4.20 | 1.62 |
| Monuron | 256.00 | 255.60 | 254.50 | 1.50 | 0.59 |
| Alanylvaline | 240.00 | 256.10 | 258.30 | -18.30 | -7.63 |
| Propyl 4-aminobenzoate | 292.46 | 257.00 | 257.00 | 35.46 | 12.12 |
| 1,2,3,4,5,6,7,8-Octahydroanthracene | 277.96 | 257.10 | 258.30 | 19.66 | 7.07 |
| TNT | 243.30 | 257.20 | 263.00 | -19.70 | -8.10 |
| 1-Benzylpyrazole | 259.61 | 257.30 |  | 2.31 | 0.89 |
| Bis(4-aminophenyl) ether | 278.20 | 257.70 | 257.10 | 21.10 | 7.58 |
| Suberamide | 245.33 | 258.30 | 260.20 | -14.87 | -6.06 |
| 3-Dimethylaminomethylindole | 283.70 | 258.70 | 248.00 | 35.70 | 12.58 |
| N-Acetylnorvaline-N'-methylamide | 239.69 | 260.50 | 263.20 | -23.51 | -9.81 |
| 1,2,3,6,7,8-Hexahydropyrene | 255.54 | 261.10 | 260.40 | -4.86 | -1.90 |
| 4,4-Methylenedianiline | 270.90 | 261.80 | 258.50 | 12.40 | 4.58 |
| Hexamethylbenzene | 245.60 | 262.10 | 262.10 | -16.50 | -6.72 |
| 3-Hydroxyflavone | 270.10 | 263.10 | 260.00 | 10.10 | 3.74 |
| Tetracene | 260.50 | 263.80 | 263.80 | -3.30 | -1.27 |
| 9,10-Benzophenanthrene | 259.20 | 263.80 | 263.90 | -4.70 | -1.81 |
| Benz[a]anthracene | 273.60 | 263.80 | 264.50 | 9.10 | 3.33 |
| 1,9-Nonanediol | 256.74 | 264.20 | 263.80 | -7.06 | -2.75 |
| Octanedioic acid | 267.60 | 264.80 | 265.10 | 2.50 | 0.93 |
| 9-Methoxy-1,4-anthraquinone | 269.21 | 265.80 | 264.60 | 4.61 | 1.71 |
| 7-Hydroxyflavone | 253.10 | 266.00 | 267.10 | -14.00 | -5.53 |
| 6-Hydroxyflavone | 250.30 | 266.00 | 267.50 | -17.20 | -6.87 |
| 4'-Bromomethyl-2-cyanobiphenyl | 257.00 | 266.40 |  | -9.40 | -3.66 |
| 5-Hydroxyflavone | 279.80 | 267.20 | 265.40 | 14.40 | 5.15 |
| [2,2]Metacyclophane | 240.60 | 268.80 | 268.20 | -27.60 | -11.47 |
| 6-Aminoflavone | 277.90 | 271.80 | 272.10 | 5.80 | 2.09 |
| Bisphenol E | 276.41 | 273.80 | 275.50 | 0.91 | 0.33 |
| p-Methacryloyloxybenzoic acid | 257.90 | 273.80 | 278.50 | -20.60 | -7.99 |
| Benzo[k]fluoranthene | 273.90 | 273.90 | 273.60 | 0.30 | 0.11 |
| Benzo[b]fluoranthene | 279.40 | 273.90 | 274.70 | 4.70 | 1.68 |
| Antipyrine | 268.20 | 274.00 |  | -5.80 | -2.16 |
| anti-trans-Truxane | 275.30 | 274.10 | 272.10 | 3.20 | 1.16 |
| N-Acetyl-D-glucosamine | 276.90 | 275.60 | 273.40 | 3.50 | 1.26 |
| 1-(4-Formylphenyl)naphthalene | 278.30 | 276.40 | 276.40 | 1.90 | 0.68 |
| [2,2]Metaparacyclophane | 261.50 | 277.50 | 277.00 | -15.50 | -5.93 |
| [2.2]Paracyclophane | 252.70 | 277.50 | 277.80 | -25.10 | -9.93 |
| p-Terphenyl | 279.60 | 277.70 | 277.40 | 2.20 | 0.79 |
| m-Terphenyl | 281.00 | 277.70 | 278.80 | 2.20 | 0.78 |
| o-Terphenyl | 274.75 | 277.70 | 277.30 | -2.55 | -0.93 |
| Benzoylglycylglycine | 277.40 | 278.90 | 278.90 | -1.50 | -0.54 |
| Perhydrophenanthrene | 289.50 | 279.10 | 280.60 | 8.90 | 3.07 |
| Arabinosylhypoxanthine | 283.30 | 279.20 | 279.10 | 4.20 | 1.48 |
| Perylene | 274.90 | 279.80 | 280.50 | -5.60 | -2.04 |
| Pentamethylbenzoic acid | 249.90 | 281.00 | 282.20 | -32.30 | -12.93 |
| trans-1,2-Dibenzoylethylene | 275.30 | 282.00 | 283.20 | -7.90 | -2.87 |
| 5,5-Diphenylhydantoin | 287.59 | 282.00 | 278.10 | 9.49 | 3.30 |
| Bisphenol S | 280.30 | 282.10 | 283.40 | -3.10 | -1.11 |
| Butyl 4-aminobenzoate | 308.00 | 282.40 | 282.90 | 25.10 | 8.15 |
| 4,4'-Dichlorodiphenyl sulphone | 269.30 | 282.80 | 289.60 | -20.30 | -7.54 |
| Alanylnorleucine | 276.00 | 283.40 | 283.50 | -7.50 | -2.72 |
| Azelamide | 270.30 | 283.80 | 285.80 | -15.50 | -5.73 |
| 1-(4-Methoxyphenyl)naphthalene | 282.50 | 284.30 | 285.00 | -2.50 | -0.88 |
| N-Benzoyl-N-methylbenzamide | 287.14 | 284.80 | 284.80 | 2.34 | 0.81 |
| N-Methylephedrine | 298.89 | 285.30 | 280.80 | 18.09 | 6.05 |
| Octyl urea | 278.70 | 287.00 | 289.00 | -10.30 | -3.70 |
| Vidarabine | 290.10 | 288.80 |  | 1.30 | 0.45 |
| 1,3-Di-t-butyl urea | 295.60 | 289.10 | 282.60 | 13.00 | 4.40 |
| 1,10-Decanediol | 279.26 | 289.70 | 290.00 | -10.74 | -3.85 |
| Indeno[1,2,3-cd]pyrene | 297.90 | 290.00 | 289.80 | 8.10 | 2.72 |
| Nonanedioic acid | 294.50 | 290.20 | 289.60 | 4.90 | 1.66 |
| cis-5a,6,11a,12-Tetrahdro[1,4]benzoxazino[3,2-b]-[1,4]-benzoxazine | 290.71 | 290.30 | 283.30 | 7.41 | 2.55 |
| 4-t-Butylbiphenyl | 304.73 | 290.50 | 290.20 | 14.53 | 4.77 |
| 4-Aminoantipyrine | 294.60 | 291.00 |  | 3.60 | 1.22 |
| Triptycene | 283.10 | 291.70 | 293.90 | -10.80 | -3.81 |
| 4,4'-Diaminodiphenyldisulfide | 294.00 | 291.90 |  | 2.10 | 0.71 |
| 2-(6-Methoxynaphthalen-2-yl)propanoic acid | 296.30 | 293.00 | 292.70 | 3.60 | 1.21 |
| 4,4'-Diaminodiphenyl sulfone | 314.90 | 293.70 | 287.60 | 27.30 | 8.67 |
| 3,3'-Dihydroxy-4,4'-diaminodiphenylmethane | 281.20 | 293.90 | 296.10 | -14.90 | -5.30 |
| Dibenzo[a,e]cyclooctene-5,11-dicarbonitrile | 299.80 | 294.60 | 294.10 | 5.70 | 1.90 |
| Dimethyl-2,6-naphthalinedicarboxylate | 283.80 | 296.20 | 297.10 | -13.30 | -4.69 |
| Bisphenol A | 301.34 | 296.90 | 298.00 | 3.34 | 1.11 |
| Tetramethylthiuram disulfide | 301.70 | 297.10 |  | 4.60 | 1.52 |
| 4'-Propylbiphenyl-4-carbonitrile | 302.97 | 297.60 | 297.10 | 5.87 | 1.94 |
| 1,2-Dibenzoylethane | 291.20 | 299.00 | 299.60 | -8.40 | -2.88 |
| Triphenylamine | 297.50 | 299.10 | 299.50 | -2.00 | -0.67 |
| 1,2-Diphenylbenzimidazole | 318.70 | 300.30 | 297.20 | 21.50 | 6.75 |
| Tritane | 308.80 | 301.00 | 300.50 | 8.30 | 2.69 |
| 3,3',4,4'-Tetraaminodiphenyl ether | 319.70 | 301.40 | 294.70 | 25.00 | 7.82 |
| Ibuprofen | 328.01 | 301.60 | 302.00 | 26.01 | 7.93 |
| 1-Benzyl-3-methylimidazolium chloride | 302.40 | 302.40 |  | 0.00 | 0.00 |
| 2-(4-Nitrobenzoylhydrazone)-propionic acid | 315.93 | 302.80 | 289.90 | 26.03 | 8.24 |
| 2,2'-Diindanyl | 332.60 | 302.80 | 301.00 | 31.60 | 9.50 |
| 1,4-Di-t-butylbenzene | 323.15 | 303.30 | 301.90 | 21.25 | 6.58 |
| 4,4'-Diisocyanatodiphenylmethane | 307.00 | 303.60 |  | 3.40 | 1.11 |
| Pentyl 4-aminobenzoate | 308.70 | 307.90 | 306.90 | 1.80 | 0.58 |
| Tetracyclopropylsuccinonitrile | 321.80 | 308.90 | 308.90 | 12.90 | 4.01 |
| Sebacamide | 289.66 | 309.20 | 308.20 | -18.54 | -6.40 |
| PMC | 315.30 | 309.50 | 307.40 | 7.90 | 2.51 |
| 4-(Phenylmethoxy)benzeneacetic acid | 340.00 | 310.40 | 311.00 | 29.00 | 8.53 |
| Ellagic acid | 328.85 | 311.20 | 304.10 | 24.75 | 7.53 |
| Triphenylethylene | 309.20 | 311.80 | 312.70 | -3.50 | -1.13 |
| 4-t-Butyldiphenyl ether | 304.06 | 311.90 | 312.30 | -8.24 | -2.71 |
| Coronene | 317.17 | 312.00 | 313.70 | 3.47 | 1.09 |
| 1-Methyl-7-isopropylphenanthrene | 294.60 | 312.20 | 313.50 | -18.90 | -6.42 |
| t-Butyl 1-phenylethylcarbamate | 313.81 | 312.30 | 308.80 | 5.01 | 1.60 |
| Triphenylmethanol | 318.80 | 313.30 | 312.90 | 5.90 | 1.85 |
| 3,4,4'-Trichlorocarbanilide | 284.13 | 313.50 | 317.20 | -33.07 | -11.64 |
| Isoproturon | 293.28 | 314.00 | 322.10 | -28.82 | -9.83 |
| 1,11-Undecanediol | 297.79 | 315.10 | 315.40 | -17.61 | -5.91 |
| Decanedioic acid | 321.40 | 315.70 | 316.00 | 5.40 | 1.68 |
| Pentacene | 311.50 | 315.80 | 316.30 | -4.80 | -1.54 |
| 1-(3-Formyl-4-methoxyphenyl)naphthalene | 313.80 | 316.00 | 318.90 | -5.10 | -1.63 |
| [3,3]Paracyclophane | 324.30 | 316.90 | 316.10 | 8.20 | 2.53 |
| 1,2'-Dinaphthylmethane | 314.60 | 322.20 | 322.80 | -8.20 | -2.61 |
| 1-(3,4-Dimethoxyphenyl)naphthalene | 321.60 | 323.90 | 327.80 | -6.20 | -1.93 |
| Perfluorobiphenyl | 317.40 | 323.90 | 338.90 | -21.50 | -6.77 |
| 1,1,1-Triphenylethane | 316.70 | 324.00 | 324.40 | -7.70 | -2.43 |
| 1,1,2-Triphenylethane | 319.70 | 326.40 | 327.60 | -7.90 | -2.47 |
| Trolox | 332.00 | 328.40 | 328.70 | 3.30 | 0.99 |
| 1,8-Diphenylnaphthalene | 328.30 | 329.70 | 328.90 | -0.60 | -0.18 |
| 1-(Biphenyl-4-yl)naphthalene | 328.30 | 329.70 | 330.30 | -2.00 | -0.61 |
| 1,4-Diphenylnaphthalene | 324.00 | 329.70 | 329.10 | -5.10 | -1.57 |
| Hexyl phenylcarbamate | 336.80 | 331.60 | 328.20 | 8.60 | 2.55 |
| 3-Phenyl-5-phenoxymethyl-2-oxazolidinone | 310.60 | 332.50 | 335.60 | -25.00 | -8.05 |
| Hexyl 4-aminobenzoate | 342.20 | 333.30 | 332.20 | 10.00 | 2.92 |
| Undecanediamide | 314.53 | 334.70 | 334.50 | -19.97 | -6.35 |
| Dicumyl | 321.10 | 336.90 | 336.70 | -15.60 | -4.86 |
| Tri-t-butylmethane | 354.80 | 339.50 | 338.20 | 16.60 | 4.68 |
| 1,12-Dodecanediol | 330.23 | 340.60 | 341.50 | -11.27 | -3.41 |
| Undecanedioic acid | 348.30 | 341.10 | 341.10 | 7.20 | 2.07 |
| 2,4,6-Triphenylpyridine | 358.60 | 343.10 | 340.30 | 18.30 | 5.10 |
| N-Benzoyl-o-aminodiphenylamine | 356.60 | 348.60 | 348.70 | 7.90 | 2.22 |
| Tri-t-butylmethanol | 350.60 | 351.80 | 356.80 | -6.20 | -1.77 |
| 4-Nitro-4'-t-butyl-diphenylether | 359.52 | 353.10 | 352.20 | 7.32 | 2.04 |
| Bisphenol AP | 365.85 | 356.10 | 357.70 | 8.15 | 2.23 |
| Perchlorobiphenyl | 344.00 | 356.60 | 369.60 | -25.60 | -7.44 |
| 1-Tridecanol | 378.00 | 358.40 | 358.50 | 19.50 | 5.16 |
| Dodecanediamide | 327.84 | 360.10 | 363.40 | -35.56 | -10.85 |
| 1,3,5-Triphenylbenzene | 361.00 | 362.60 | 362.00 | -1.00 | -0.28 |
| p-Quaterphenyl | 363.50 | 362.60 | 362.20 | 1.30 | 0.36 |
| m-Quaterphenyl | 359.50 | 362.60 | 362.00 | -2.50 | -0.70 |
| o-Quaterphenyl | 359.10 | 362.60 | 363.20 | -4.10 | -1.14 |
| 1,2,3-Triphenylbenzene | 352.37 | 362.60 | 363.70 | -11.33 | -3.22 |
| 3,5-Di-t-butylsalicylic acid | 383.00 | 365.20 | 364.00 | 19.00 | 4.96 |
| 1,13-Tridecanediol | 366.88 | 366.00 | 365.40 | 1.48 | 0.40 |
| Dodecanedioic acid | 375.20 | 366.60 | 365.80 | 9.40 | 2.51 |
| N-(3-Fluoro-5-(3-pyridinyloxy)phenyl)-N'-3-pyridinylurea | 362.32 | 372.40 | 378.90 | -16.58 | -4.58 |
| Tridecanoic acid | 387.60 | 373.10 | 373.10 | 14.50 | 3.74 |
| 1,1'-Diphenyl-1,1'-bicyclopentyl | 375.50 | 377.60 | 376.60 | -1.10 | -0.29 |
| Tetraisopropoxysilane | 359.48 | 382.70 | 391.90 | -32.42 | -9.02 |
| Tetraphenylmethane | 368.20 | 383.30 | 385.00 | -16.80 | -4.56 |
| Myristyl alcohol | 388.00 | 383.80 | 383.70 | 4.30 | 1.11 |
| Tetrapropoxysilane | 363.39 | 387.40 | 396.40 | -33.01 | -9.08 |
| 4,4'-Di-t-butylbiphenyl | 412.41 | 388.30 | 387.10 | 25.31 | 6.14 |
| 1,14-Tetradecanediol | 379.61 | 391.50 | 391.10 | -11.49 | -3.03 |
| 1,13-Tridecanedioic acid | 402.10 | 392.00 | 392.20 | 9.90 | 2.46 |
| 1,8-Di(4-formylphenyl)naphthalene | 393.50 | 393.00 | 392.80 | 0.70 | 0.18 |
| Indapamide | 396.31 | 394.00 |  | 2.31 | 0.58 |
| Tetraphenylsilane | 395.80 | 395.70 |  | 0.10 | 0.03 |
| Tetraphenylethylene | 388.70 | 397.00 | 396.60 | -7.90 | -2.03 |
| 1-Monocaprin | 410.00 | 397.30 | 396.50 | 13.50 | 3.29 |
| Tetradecanoic acid | 432.01 | 398.50 | 399.30 | 32.71 | 7.57 |
| 2-Pentadecanone | 426.80 | 407.30 | 407.00 | 19.80 | 4.64 |
| 1,1,1,2-Tetraphenylethane | 395.40 | 408.70 | 408.80 | -13.40 | -3.39 |
| 1,8-Di(4-methoxyphenyl)naphthalene | 399.20 | 408.90 | 409.70 | -10.50 | -2.63 |
| 1-Pentadecanol | 400.00 | 409.30 | 409.40 | -9.40 | -2.35 |
| 1,1,2,2-Tetraphenylethane | 399.60 | 409.30 | 410.40 | -10.80 | -2.70 |
| Nonyl 4-aminobenzoate | 422.60 | 409.70 | 409.40 | 13.20 | 3.12 |
| 4-Hexyloxybenzylideneaniline | 404.52 | 411.00 | 412.30 | -7.78 | -1.92 |
| Tetradecanediamide | 383.31 | 411.00 | 414.40 | -31.09 | -8.11 |
| N-(4-Ethoxybenzylidene)-4-butylaniline | 429.09 | 411.20 | 409.70 | 19.39 | 4.52 |
| Lactose | 412.50 | 414.90 | 415.80 | -3.30 | -0.80 |
| 1,15-Pentadecanediol | 377.45 | 416.90 | 417.80 | -40.35 | -10.69 |
| Maltose | 434.70 | 417.30 | 412.40 | 22.30 | 5.13 |
| Cellobiose | 436.10 | 417.30 | 412.20 | 23.90 | 5.48 |
| 1,14-Tetradecanedioic acid | 429.00 | 417.50 | 415.60 | 13.40 | 3.12 |
| Saccharose | 424.30 | 418.20 | 417.70 | 6.60 | 1.56 |
| 1-Pentadecanoic acid | 443.28 | 424.00 | 421.80 | 21.48 | 4.85 |
| 4-Hexyloxybenzylideneamino-4'-fluorobenzene | 442.55 | 424.10 | 422.30 | 20.25 | 4.58 |
| 1,1'-Diphenyl-1,1'-bicyclohexyl | 403.80 | 425.60 | 427.60 | -23.80 | -5.89 |
| 4-Hexyloxybenzylideneamino-4'-chlorobenzene | 434.94 | 427.40 | 427.20 | 7.74 | 1.78 |
| Cetane | 441.80 | 430.50 | 430.50 | 11.30 | 2.56 |
| 1-Hexadecanol | 422.00 | 434.70 | 434.80 | -12.80 | -3.03 |
| 4-Hexyloxybenzylidene-4'-toluidine | 441.40 | 436.70 | 436.50 | 4.90 | 1.11 |
| N-(4-Hexyloxybenzylidene)-4-aminobenzonitrile | 432.51 | 439.20 | 439.50 | -6.99 | -1.62 |
| cis-(5,12)-7,7,14,14-Hexamethyl-1,4,8,11-tetraazacyclotetradecane | 444.80 | 440.50 |  | 4.30 | 0.97 |
| Glyceryl-2-laurate | 436.40 | 441.10 | 442.00 | -5.60 | -1.28 |
| 1,16-Hexadecanediol | 426.18 | 442.40 | 443.10 | -16.92 | -3.97 |
| Vitamin B2 | 442.20 | 445.90 | 448.10 | -5.90 | -1.33 |
| N,N'-Bis-(m-methoxyphenyl)terephtalamide | 458.10 | 447.40 | 447.20 | 10.90 | 2.38 |
| N,N'-Bis(p-methoxyphenyl)terephthalamide | 467.80 | 447.40 | 442.60 | 25.20 | 5.39 |
| m-Quinquephenyl | 443.70 | 447.60 | 448.50 | -4.80 | -1.08 |
| o-Quinquephenyl | 444.30 | 447.60 | 449.30 | -5.00 | -1.13 |
| p-Quinquephenyl | 455.50 | 447.60 | 447.60 | 7.90 | 1.73 |
| 1-Glyceryl laurate | 447.70 | 448.20 | 449.90 | -2.20 | -0.49 |
| Palmitic acid | 448.00 | 449.40 | 449.60 | -1.60 | -0.36 |
| Dibenzo-18-crown-6 | 439.70 | 461.00 | 466.50 | -26.80 | -6.10 |
| p-Diacetylbenzene diethyl ketal | 462.00 | 468.30 | 477.60 | -15.60 | -3.38 |
| Hexadecanedioic acid | 482.80 | 468.40 | 467.50 | 15.30 | 3.17 |
| Methylprednisolone | 472.10 | 469.80 | 464.10 | 8.00 | 1.69 |
| 1,8-Di(3-formyl-4-methoxyphenyl)naphthalene | 458.20 | 472.20 | 478.40 | -20.20 | -4.41 |
| Methyl hexadecanoate | 474.47 | 473.00 | 472.70 | 1.77 | 0.37 |
| Margaric acid | 475.70 | 474.90 | 475.60 | 0.10 | 0.02 |
| 2,4,6-Trimethyl-2,4,6-triphenylcyclotrisiloxane | 506.70 | 477.60 |  | 29.10 | 5.74 |
| Octadecane | 485.64 | 481.40 | 480.30 | 5.34 | 1.10 |
| Tetra-2-methyl-1-propoxysilane | 477.59 | 481.70 | 481.20 | -3.61 | -0.76 |
| Bis-(o-aminophenyl)-2,2'-dibenzimidazole oxide | 471.10 | 483.70 | 489.30 | -18.20 | -3.86 |
| Tetra-2-butoxysilane | 487.27 | 484.50 | 485.00 | 2.27 | 0.47 |
| N-(4-Pentyloxybenzylidene)-4-butylaniline | 478.20 | 487.60 | 489.00 | -10.80 | -2.26 |
| 1,8-Di(3,4-dimethoxyphenyl)naphthalene | 470.10 | 488.10 | 495.00 | -24.90 | -5.30 |
| Di(4'-methoxyphenyl)cyclohexane-1,4-dicarboxylate | 478.60 | 488.90 | 489.40 | -10.80 | -2.26 |
| Tetrabutoxysilane | 493.68 | 489.20 | 489.00 | 4.68 | 0.95 |
| 1,4-Bis(diphenylamino)benzene | 470.60 | 490.50 | 502.30 | -31.70 | -6.74 |
| Pentaphenylethane | 473.60 | 491.50 | 492.80 | -19.20 | -4.05 |
| 2-Monomyristin | 506.30 | 492.00 | 490.70 | 15.60 | 3.08 |
| Risperidal | 502.00 | 494.40 | 487.20 | 14.80 | 2.95 |
| 1,8-Bis(biphenyl-4-yl)naphthalene | 490.90 | 499.60 | 499.90 | -9.00 | -1.83 |
| 1,2,3,4-Tetraphenylnaphthalene | 491.60 | 499.60 | 501.40 | -9.80 | -1.99 |
| Stearic acid | 501.60 | 500.30 | 497.60 | 4.00 | 0.80 |
| 4-Nitrophenyl-4'-octyloxybenzoate | 510.00 | 510.00 | 510.40 | -0.40 | -0.08 |
| N-(4-Hexyloxybenzylidene)-4-butylaniline | 512.02 | 513.00 | 513.50 | -1.48 | -0.29 |
| 1-Nonadecanoic acid | 525.40 | 525.80 | 526.20 | -0.80 | -0.15 |
| 4,4'-Bis-(N-carbazolyl) -1,1'-biphenyl | 539.36 | 529.80 | 524.40 | 14.96 | 2.77 |
| 2,3-Dimethyl-2,3-bis(4-t-butylphenyl)butane | 529.50 | 532.40 | 532.70 | -3.20 | -0.60 |
| 2-Monopalmitin | 558.60 | 542.90 | 541.70 | 16.90 | 3.03 |
| 1-Palmitoylglycerol | 566.90 | 550.00 | 549.30 | 17.60 | 3.10 |
| Arachidic acid | 545.10 | 551.20 | 551.10 | -6.00 | -1.10 |
| 2,11-Dicyclohexyldodecane | 557.30 | 562.70 | 563.70 | -6.40 | -1.15 |
| 1,1-Dicyclohexyldodecane | 562.60 | 564.60 | 565.30 | -2.70 | -0.48 |
| N-Formylmethionine-leucyl-phenylalanine | 567.60 | 569.70 |  | -2.10 | -0.37 |
| Dibenzo-24-crown-8-ether | 577.00 | 570.20 | 567.20 | 9.80 | 1.70 |
| Methyl arachidate | 578.99 | 574.80 | 571.90 | 7.09 | 1.22 |
| Docosane | 563.60 | 583.20 | 586.00 | -22.40 | -3.97 |
| 3,4-Dimethyl-3,4-bis(4-t-butylphenyl)hexane | 587.90 | 583.30 | 583.40 | 4.50 | 0.77 |
| Tetra-3-methyl-1-butoxysilane | 591.28 | 583.50 | 581.90 | 9.38 | 1.59 |
| Tetra-2-pentoxysilane | 610.23 | 586.30 | 580.50 | 29.73 | 4.87 |
| Simvastatin | 577.00 | 589.80 | 591.50 | -14.50 | -2.51 |
| Tetrapentoxysilane | 621.56 | 591.00 | 584.00 | 37.56 | 6.04 |
| N-Formylmethionine-leucyl-phenylalanine methyl ether | 597.60 | 593.20 |  | 4.40 | 0.74 |
| 2-Stearoylglycerol | 610.40 | 593.80 | 593.50 | 16.90 | 2.77 |
| 1-Glyceryl stearate | 610.40 | 600.90 | 600.30 | 10.10 | 1.65 |
| Methylprednisolone aceponate | 604.80 | 615.50 | 617.20 | -12.40 | -2.05 |
| 3,4-Diethyl-3,4-bis(4-t-butylphenyl)-hexane | 631.30 | 634.80 | 637.50 | -6.20 | -0.98 |
| Tetramethyltetraphenylcyclotetrasiloxane | 615.00 | 636.80 |  | -21.80 | -3.54 |
| 2,3,6-Tris(phenylimino)-1,3,5-triphenyl-1,3,5-triazacyclohexane | 672.40 | 673.20 |  | -0.80 | -0.12 |
| Dibenzo-30-crown-10 | 680.40 | 679.50 | 679.60 | 0.80 | 0.12 |
| 2,4,5,7-Tetramethyl-4,5-bis(4-t-butylphenyl)octane | 683.90 | 681.40 | 679.60 | 4.30 | 0.63 |
| Hexacosane | 661.20 | 685.00 | 688.20 | -27.00 | -4.08 |
| 4,5-Dipropyl-4,5-bis-(4-t-butylphenyl)octane | 724.20 | 736.00 | 739.60 | -15.40 | -2.13 |
| Triacontane | 808.80 | 786.80 | 781.80 | 27.00 | 3.34 |
| 1,1,3,3,5,5-Hexaphenyl-7,7-dimethylcyclotetrasiloxane | 815.50 | 815.10 |  | 0.40 | 0.05 |
| Dotriacontane | 806.00 | 837.70 | 840.80 | -34.80 | -4.32 |
| 5,6-Dibutyl-5,6-bis(4-t-butylphenyl)decane | 805.50 | 837.80 | 841.60 | -36.10 | -4.48 |
| Tetratriacontane | 887.40 | 888.60 | 892.50 | -5.10 | -0.57 |
| Tetra-2-ethyl-1-hexoxysilane | 870.88 | 888.90 | 897.90 | -27.02 | -3.10 |
| Pentatriacontane | 915.90 | 914.10 | 913.80 | 2.10 | 0.23 |
| Octaphenylpentacyclosilsesquioxane | 1129.00 | 1146.60 |  | -17.60 | -1.56 |
