# Supplementary material for: Calculation of the Isobaric Heat Capacities of the Liquid and Solid Phase of Organic Compounds at 298.15K by Means of the Group-Additivity Method
Source: Molecules. 2020 Mar 4;25(5):1147. doi: 10.3390/molecules25051147 (PMC7179130; doi:10.3390/molecules25051147)
Supplement: Supplementary file 1 [file molecules-25-01147-s001.zip › Table 1.doc]

| **Atom Type** | **Neighbours** | **Meaning^a^** | **Example** |
| --- | --- | --- | --- |
| B(-) | F4 | **B**F_4_^-^ | tetrafluoroborate |
| C sp3 | H2CN(+) | C**C**H_2_N(+) | C1 in tetraalkylammonium |
| C sp3 | H2CP(+) | C**C**H_2_P(+) | C1 in tetraalkylphosphonium |
| C sp3 | H2CS(+) | C**C**H_2_S(+) | C1 in trialkylsulfonium |
| C(-) sp3 | C3 | C_3_**C**^-^ | central C^-^ in tricyanocarbeniate |
| C aromatic | H:C:N(+) | C:**C**H:N^+^ | C2 in pyridinium |
| C(+) aromatic | H:N2 | N:**C**^+^H:N | C2 in imidazolium |
| C sp | B#N(-) | B^-^(**C**#N) | C in tetracyanoborate |
| C sp | C#N(-) | C^-^(**C**#N) | cyano-C in tricyanocarbeniate |
| C sp | N#N(-) | N^-^(**C**#N) | C in dicyanoamide |
| C sp | =N=S(-) | N=**C**=S^-^ | thiocyanate |
| N(+) sp3 | C4 | **N**^+^C_4_ | tetraalkylammonium |
| N(+) sp2 | O2=O(-) | **N**O_3_^-^ | nitrate |
| N aromatic | C2:C(+) | C-**N**(C):C^+^ | N1 and N3 in 1,3-dialkylimidazolium |
| N(+) aromatic | C:C2 | C:**N**^+^(C):C | N in 1-alkylpyridinium |
| N(-) | C2 | C-**N**^-^-C | N^-^ in dicyanoamide |
| N(-) | CS | C-**N**^-^-S | N^-^ in saccharinate |
| N(-) | S2 | S-**N**^-^-S | bis(trifluoromethanesulfonyl)amide |
| P4 | CO2=O(-) | C**P**O3^-^ | alkylphosphonate |
| P(+) | C4 | **P**C_4_^+^ | tetraalkylphosphonium |
| P(-) | C3F3 | F_3_**P**^-^C_3_ | tris(pentafluoroethyl)trifluorophosphate |
| P(-) | F6 | **P**F_6_^-^ | hexafluorophosphate |
| S(+) | C3 | C_3_**S**^+^ | trialkylsulfonium |
| S4 | CN=O2(-) | C**S**(O_2_)N^-^ | bis(trifluoromethanesulfonyl)amide |
| S4 | CO=O2(-) | C**S**O_3_^-^ | alkylsulfonate |
| S4 | O2=O2(-) | **S**O_4_^-^ | alkylsulfate |
