# Supplementary material for: Calculation of the Isobaric Heat Capacities of the Liquid and Solid Phase of Organic Compounds at 298.15K by Means of the Group-Additivity Method
Source: Molecules. 2020 Mar 4;25(5):1147. doi: 10.3390/molecules25051147 (PMC7179130; doi:10.3390/molecules25051147)
Supplement: Supplementary file 1 [file molecules-25-01147-s001.zip › Table 10.doc]

| **Molecule name** | **C_p_(sol,298) GA-calc.** | | **C_p_(sol,298) exp.** | **C_p_(sol,298) V_m_-calc.[1]** | |
| --- | --- | --- | --- | --- | --- |
|  | **Non-ionic** | **Zwitter-ionic** |  | **Zwitter-ionic** | **Non-ionic** |
| Glycine | 120.20 | 89.90 | 99.30 | 95.90 | 91.30 |
| Alanine | 147.60 | 116.50 | 119.90 | 118.50 | 116.10 |
| N-Methylglycine | 136.00 | 122.40 | 118.20 | 119.10 | 116.90 |
| Serine | 152.70 | 121.70 | 135.60 | 126.20 | 126.50 |
| α-Aminobutyric acid | 173.00 | 142.00 | 146.40 | 140.90 | 140.70 |
| Proline | 162.20 | 137.30 | 150.40 | 149.80 | 150.90 |
| Threonine | 183.00 | 152.00 | 155.31 | 153.30 | 154.70 |
| Aspartic acid | 193.00 | 162.00 | 155.18 | 155.10 | 156.00 |
| Asparagine | 189.80 | 158.70 | 159.80 | 157.60 | 159.30 |
| Valine | 196.70 | 165.60 | 165.00 | 162.90 | 165.40 |
| 5-Aminopentanoic acid | 196.60 | 166.30 | 163.70 | 164.30 | 166.60 |
| Ornithine | 225.40 | 194.40 | 191.20 | 179.20 | 183.60 |
| Glutamine | 214.00 | 183.00 | 184.18 | 182.30 | 186.50 |
| Leucine | 222.10 | 191.10 | 200.80 | 183.90 | 188.20 |
| Isoleucine | 222.10 | 191.10 | 188.28 | 184.70 | 189.90 |
| Methionine | 238.50 | 207.40 | 205.16 | 189.10 | 194.30 |
| N-Phenylglycine | 196.10 | 162.20 | 177.40 | 194.60 | 198.60 |
| Phenylalanine | 232.20 | 201.10 | 203.10 | 215.50 | 221.60 |
| 8-Aminooctanoic acid | 273.00 | 242.70 | 251.70 | 232.80 | 241.90 |
| Tyrosine | 248.20 | 217.10 | 216.44 | 236.20 | 238.10 |
| Tryptophane | 268.30 | 237.20 | 238.15 | 252.00 | 263.90 |
