# Supplementary material for: Calculation of the Isobaric Heat Capacities of the Liquid and Solid Phase of Organic Compounds at 298.15K by Means of the Group-Additivity Method
Source: Molecules. 2020 Mar 4;25(5):1147. doi: 10.3390/molecules25051147 (PMC7179130; doi:10.3390/molecules25051147)
Supplement: Supplementary file 1 [file molecules-25-01147-s001.zip › Table 2.doc]

| **Atom Type** | **Neighbours** | **Meaning** |
| --- | --- | --- |
| O(prim) | HC | Primary alcohol |
| O(sec) | HC | Secondary alcohol |
| O(tert) | HC | Tertiary alcohol |
| Endocyclic bonds | No of single bonds | Count single bonds in cyclic ring |
| Angle60 |  | Bond angle <60 deg |
| Angle 90 |  | Bond angle between 60 and 90 deg |
| Angle102 |  | Bond angle between 90 and 102 deg |
| (COH)n | n>1 | Molecule contains more than 1 OH group |
| H | H Acceptor | Intramolecular H bridge between acidic H (on O, N or S) and basic acceptor (O, N or F) |
