# Supplementary material for: Calculation of the Isobaric Heat Capacities of the Liquid and Solid Phase of Organic Compounds at 298.15K by Means of the Group-Additivity Method
Source: Molecules. 2020 Mar 4;25(5):1147. doi: 10.3390/molecules25051147 (PMC7179130; doi:10.3390/molecules25051147)
Supplement: Supplementary file 1 [file molecules-25-01147-s001.zip › Table 3.doc]

| **Entry** | **Atom Type** | **Neighbours** | **Contribution** | **Occurrences** | **Molecules** |
| --- | --- | --- | --- | --- | --- |
| 1 | B | C3 | 240 | 1 | 1 |
| 2 | B(-) | C4 | 698.66 | 2 | 2 |
| 3 | B(-) | F4 | 51.21 | 6 | 6 |
| 4 | C sp3 | H3C | 37.03 | 1555 | 790 |
| 5 | C sp3 | H3N | 100.02 | 127 | 101 |
| 6 | C sp3 | H3N(+) | 147.91 | 20 | 18 |
| 7 | C sp3 | H3O | 81.29 | 84 | 66 |
| 8 | C sp3 | H3S | 84.43 | 17 | 13 |
| 9 | C sp3 | H3S(+) | 172.17 | 1 | 1 |
| 10 | C sp3 | H3P | 217.59 | 1 | 1 |
| 11 | C sp3 | H3Si | 71 | 71 | 18 |
| 12 | C sp3 | H2BC | -37.03 | 3 | 1 |
| 13 | C sp3 | H2C2 | 30.06 | 3249 | 696 |
| 14 | C sp3 | H2CN | 90.52 | 222 | 146 |
| 15 | C sp3 | H2CN(+) | 142.85 | 78 | 52 |
| 16 | C sp3 | H2CO | 73.86 | 477 | 243 |
| 17 | C sp3 | H2CS | 75.25 | 38 | 27 |
| 18 | C sp3 | H2CS(+) | 136.25 | 29 | 10 |
| 19 | C sp3 | H2CP | 252.08 | 2 | 1 |
| 20 | C sp3 | H2CP(+) | 71.8 | 12 | 3 |
| 21 | C sp3 | H2CCl | 63.67 | 38 | 30 |
| 22 | C sp3 | H2CBr | 63.93 | 26 | 21 |
| 23 | C sp3 | H2CJ | 67.73 | 10 | 9 |
| 24 | C sp3 | H2CSi | 60.71 | 18 | 8 |
| 25 | C sp3 | H2N2 | 151.55 | 4 | 2 |
| 26 | C sp3 | H2NO | 157.51 | 12 | 12 |
| 27 | C sp3 | H2O2 | 111.47 | 4 | 4 |
| 28 | C sp3 | H2S2 | 128.41 | 1 | 1 |
| 29 | C sp3 | HC3 | 21.11 | 303 | 196 |
| 30 | C sp3 | HC2N | 81.86 | 14 | 14 |
| 31 | C sp3 | HC2N(+) | 159.6 | 3 | 3 |
| 32 | C sp3 | HC2O | 67.45 | 107 | 87 |
| 33 | C sp3 | HC2S | 67.46 | 10 | 9 |
| 34 | C sp3 | HC2Si | 36.48 | 1 | 1 |
| 35 | C sp3 | HC2Cl | 56.56 | 9 | 9 |
| 36 | C sp3 | HC2Br | 56.84 | 4 | 4 |
| 37 | C sp3 | HC2J | 62.21 | 2 | 2 |
| 38 | C sp3 | HCNO(+) | 176.8 | 3 | 1 |
| 39 | C sp3 | HCO2 | 96.7 | 3 | 3 |
| 40 | C sp3 | HCF2 | 157.26 | 1 | 1 |
| 41 | C sp3 | HCFCl | 73.89 | 1 | 1 |
| 42 | C sp3 | HCCl2 | 86.15 | 9 | 8 |
| 43 | C sp3 | HCClBr | 89.68 | 1 | 1 |
| 44 | C sp3 | HCBr2 | 82.85 | 2 | 1 |
| 45 | C sp3 | C4 | 7.78 | 62 | 51 |
| 46 | C sp3 | C3N | 81.33 | 5 | 4 |
| 47 | C sp3 | C3N(+) | 55.23 | 3 | 3 |
| 48 | C sp3 | C3O | 57.44 | 23 | 21 |
| 49 | C sp3 | C3S | 57.43 | 7 | 5 |
| 50 | C sp3 | C3F | 43.66 | 5 | 3 |
| 51 | C sp3 | C3Cl | 50.92 | 1 | 1 |
| 52 | C sp3 | C3Br | 54.62 | 1 | 1 |
| 53 | C sp3 | C2N2(+) | 223.66 | 2 | 2 |
| 54 | C sp3 | C2O2 | 99.71 | 1 | 1 |
| 55 | C sp3 | C2F2 | 50.88 | 78 | 13 |
| 56 | C sp3 | C2FCl | 64.31 | 5 | 2 |
| 57 | C sp3 | C2Cl2 | 87.42 | 2 | 2 |
| 58 | C sp3 | CNF2 | 112.99 | 3 | 1 |
| 59 | C sp3 | CF3 | 66.92 | 31 | 23 |
| 60 | C sp3 | CSF2 | 0 | 1 | 1 |
| 61 | C sp3 | CPF2(-) | 44.23 | 6 | 2 |
| 62 | C sp3 | CF2Cl | 89.67 | 4 | 4 |
| 63 | C sp3 | CF2Br | 86.41 | 7 | 4 |
| 64 | C sp3 | CFCl2 | 88.11 | 3 | 2 |
| 65 | C sp3 | CCl3 | 102.55 | 8 | 8 |
| 66 | C sp3 | SF3 | 102.78 | 151 | 78 |
| 67 | C(-) sp3 | C3 | 131.76 | 1 | 1 |
| 68 | C sp2 | H2=C | 35.64 | 61 | 59 |
| 69 | C sp2 | HC=C | 22.79 | 195 | 107 |
| 70 | C sp2 | HC=N | 95.94 | 4 | 4 |
| 71 | C sp2 | HC=O | 54.97 | 23 | 23 |
| 72 | C sp2 | H=CN | 92.25 | 166 | 87 |
| 73 | C sp2 | H=CO | 42.8 | 11 | 10 |
| 74 | C sp2 | H=CS | 87.41 | 5 | 5 |
| 75 | C sp2 | H=CCl | 56.41 | 5 | 3 |
| 76 | C sp2 | H=CSi | 43.69 | 4 | 4 |
| 77 | C sp2 | HN=N | 32.91 | 3 | 3 |
| 78 | C sp2 | HN=O | 100.63 | 3 | 3 |
| 79 | C sp2 | HO=O | 58.82 | 7 | 7 |
| 80 | C sp2 | H=NS | 15.48 | 2 | 2 |
| 81 | C sp2 | C2=C | 16.22 | 54 | 44 |
| 82 | C sp2 | C2=N | 333.48 | 1 | 1 |
| 83 | C sp2 | C=CN | 89.61 | 3 | 2 |
| 84 | C sp2 | C2=O | 50.28 | 49 | 49 |
| 85 | C sp2 | C=CO | 36.16 | 5 | 5 |
| 86 | C sp2 | C=CS | 74.31 | 5 | 4 |
| 87 | C sp2 | C=CCl | 160.28 | 1 | 1 |
| 88 | C sp2 | CN=O | 87.09 | 12 | 12 |
| 89 | C sp2 | CN=O(-) | 87.78 | 1 | 1 |
| 90 | C sp2 | C=NS | 8.06 | 1 | 1 |
| 91 | C sp2 | CO=O | 43.25 | 216 | 158 |
| 92 | C sp2 | CO=O(-) | 27.63 | 8 | 7 |
| 93 | C sp2 | C=OS | 0 | 1 | 1 |
| 94 | C sp2 | C=OCl | 70.53 | 7 | 6 |
| 95 | C sp2 | =CF2 | 56.5 | 2 | 1 |
| 96 | C sp2 | =CCl2 | 77.26 | 5 | 4 |
| 97 | C sp2 | N2=N | 56.88 | 1 | 1 |
| 98 | C sp2 | N2=O | 131.83 | 3 | 3 |
| 99 | C sp2 | NO=O | 98.32 | 1 | 1 |
| 100 | C sp2 | O2=O | 50.04 | 5 | 5 |
| 101 | C aromatic | H:C2 | 22 | 1115 | 238 |
| 102 | C aromatic | H:C:N | 42.32 | 19 | 13 |
| 103 | C aromatic | H:C:N(+) | -9.45 | 53 | 32 |
| 104 | C aromatic | H:N2 | 0 |  | 0 |
| 105 | C aromatic | :C3 | 9.57 | 19 | 11 |
| 106 | C aromatic | C:C2 | 11.58 | 251 | 152 |
| 107 | C aromatic | C:C:N | 30.59 | 8 | 7 |
| 108 | C aromatic | C:C:N(+) | -2.69 | 11 | 11 |
| 109 | C aromatic | :C2N | 71.34 | 31 | 29 |
| 110 | C aromatic | :C2N(+) | 118.05 | 11 | 8 |
| 111 | C aromatic | :C2:N | 31.72 | 3 | 3 |
| 112 | C aromatic | :C2O | 33.82 | 46 | 28 |
| 113 | C aromatic | :C2S | 88.68 | 7 | 7 |
| 114 | C aromatic | :C2Si | 37.2 | 10 | 7 |
| 115 | C aromatic | :C2F | 37.06 | 54 | 17 |
| 116 | C aromatic | :C2Cl | 41.28 | 19 | 15 |
| 117 | C aromatic | :C2Br | 52.57 | 11 | 8 |
| 118 | C aromatic | :C2J | 43.43 | 3 | 3 |
| 119 | C(+) aromatic | H:N2 | -155.06 | 74 | 74 |
| 120 | C sp | B#N(-) | -130.93 | 8 | 2 |
| 121 | C sp | H#C | 38.78 | 6 | 5 |
| 122 | C sp | C#C | 23.99 | 10 | 7 |
| 123 | C sp | =C2 | 25.16 | 4 | 4 |
| 124 | C sp | C#N | 48.78 | 35 | 31 |
| 125 | C sp | C#N(-) | -9.81 | 3 | 1 |
| 126 | C sp | #CSi | 49.58 | 2 | 1 |
| 127 | C sp | N#N(-) | -2.88 | 12 | 6 |
| 128 | C sp | =N2 | -89.69 | 1 | 1 |
| 129 | C sp | =N=O | -20.75 | 8 | 5 |
| 130 | C sp | =N=S(-) | 43.63 | 3 | 3 |
| 131 | N sp3 | H2C | -4.35 | 33 | 28 |
| 132 | N sp3 | H2C(pi) | 0.31 | 9 | 9 |
| 133 | N sp3 | H2N | 48.67 | 5 | 4 |
| 134 | N sp3 | HC2 | -71.79 | 21 | 20 |
| 135 | N sp3 | HC2(pi) | -72.44 | 14 | 14 |
| 136 | N sp3 | HC2(2pi) | -103.28 | 6 | 6 |
| 137 | N sp3 | HCN | -15.74 | 4 | 3 |
| 138 | N sp3 | HCN(pi) | -13 | 1 | 1 |
| 139 | N sp3 | HCS(pi) | -21.52 | 1 | 1 |
| 140 | N sp3 | C3 | -160.23 | 33 | 28 |
| 141 | N sp3 | C3(pi) | -149.65 | 17 | 14 |
| 142 | N sp3 | C3(2pi) | -180.02 | 3 | 3 |
| 143 | N sp3 | C3(3pi) | -165.46 | 1 | 1 |
| 144 | N sp3 | C2N | -91.6 | 2 | 2 |
| 145 | N sp3 | C2N(2pi) | -143.37 | 2 | 2 |
| 146 | N sp3 | C2N(3pi) | -160.71 | 1 | 1 |
| 147 | N sp2 | H=C | -243.13 | 1 | 1 |
| 148 | N sp2 | C=C | 15.82 | 17 | 13 |
| 149 | N sp2 | C=N | -20.24 | 2 | 1 |
| 150 | N sp2 | C=N(+) | -42.22 | 1 | 1 |
| 151 | N sp2 | =CN | 0 | 3 | 3 |
| 152 | N sp2 | =CO | -53.43 | 1 | 1 |
| 153 | N aromatic | C2:C(+) | -0.31 | 148 | 74 |
| 154 | N aromatic | :C2 | -16.75 | 15 | 15 |
| 155 | N(+) sp3 | H3C | -44.33 | 1 | 1 |
| 156 | N(+) sp3 | H2C2 | -140.41 | 4 | 4 |
| 157 | N(+) sp3 | HC3 | -291.64 | 1 | 1 |
| 158 | N(+) sp3 | C4 | -372.93 | 13 | 13 |
| 159 | N(+) sp2 | C=NO(-) | 0 | 1 | 1 |
| 160 | N(+) sp2 | CO=O(-) | -45.71 | 25 | 17 |
| 161 | N(+) sp2 | O2=O(-) | 5.99 | 4 | 4 |
| 162 | N(+) aromatic | C:C2 | 14.22 | 32 | 32 |
| 163 | N(-) | C2 | 62.36 | 6 | 6 |
| 164 | N(-) | CS | -32.57 | 1 | 1 |
| 165 | N(-) | S2 | 33.36 | 73 | 73 |
| 166 | O(prim) | HC | 14.35 | 102 | 89 |
| 167 | O(sec) | HC | 36.17 | 47 | 47 |
| 168 | O(tert) | HC | 58 | 11 | 11 |
| 169 | O | HC(pi) | 48.39 | 57 | 46 |
| 170 | O | HP | -119.34 | 1 | 1 |
| 171 | O | HS | 39.11 | 1 | 1 |
| 172 | O | C2 | -59.32 | 170 | 98 |
| 173 | O | C2(pi) | -26.57 | 191 | 149 |
| 174 | O | C2(2pi) | -15.47 | 22 | 12 |
| 175 | O | CN(+)(pi) | 55.55 | 3 | 3 |
| 176 | O | CN(2pi) | 0 | 1 | 1 |
| 177 | O | CS | 16.03 | 8 | 8 |
| 178 | O | CP(pi) | 22.25 | 3 | 1 |
| 179 | O | CSi | -22.04 | 20 | 5 |
| 180 | O | Si2 | -21.83 | 19 | 7 |
| 181 | P4 | C2O=O(-) | -344.96 | 1 | 1 |
| 182 | P4 | CO2=O(-) | 0 | 1 | 1 |
| 183 | P4 | O3=O | 0 | 1 | 1 |
| 184 | P(+) | C4 | -95.06 | 3 | 3 |
| 185 | P(-) | C3F3 | 33.12 | 2 | 2 |
| 186 | P(-) | F6 | 96.53 | 9 | 9 |
| 187 | S2 | HC | 0.94 | 19 | 19 |
| 188 | S2 | HC(pi) | -25.44 | 1 | 1 |
| 189 | S2 | C2 | -53.94 | 19 | 19 |
| 190 | S2 | C2(pi) | -77.07 | 2 | 2 |
| 191 | S2 | C2(2pi) | -89.86 | 7 | 7 |
| 192 | S2 | CS | -11.11 | 8 | 4 |
| 193 | S4 | C2=O | -23.45 | 2 | 2 |
| 194 | S4 | C2=O2 | -18.86 | 1 | 1 |
| 195 | S4 | CN=O2 | 0 | 1 | 1 |
| 196 | S4 | CN=O2(-) | 5.69 | 147 | 74 |
| 197 | S4 | CO=O2(-) | 4.61 | 9 | 9 |
| 198 | S4 | O2=O2(-) | 0 | 9 | 9 |
| 199 | S(+) | C3 | -203.28 | 10 | 10 |
| 200 | Si | C4 | -89.09 | 11 | 10 |
| 201 | Si | C3O | -49.41 | 6 | 3 |
| 202 | Si | C3Cl | -25.49 | 1 | 1 |
| 203 | Si | C2O2 | 9.5 | 16 | 6 |
| 204 | Si | C2Cl2 | 25.44 | 3 | 3 |
| 205 | Si | CCl3 | 86.83 | 3 | 3 |
| 206 | Si | O4 | 0 | 5 | 5 |
| 207 | (COH)n | n>1 | -27.73 | 20 | 19 |
| 208 | H | H Acceptor | -21.43 | 3 | 3 |
| 209 | Endocyclic bonds | No of single bds | -3.92 | 1341 | 243 |
| 210 | Angle60 |  | 4.13 | 69 | 19 |
| 211 | Angle90 |  | 1.7 | 63 | 19 |
| A | Based on | Valid groups | 134 |  | 1176 |
| B | Goodness of fit | R^2^ | 0.998 |  | 1111 |
| C | Deviation | Average | 6.09 |  | 1111 |
| D | Deviation | Standard | 8.24 |  | 1111 |
| E | K-fold cv | K | 10 |  | 1060 |
| F | Goodness of fit | Q^2^ | 0.9975 |  | 1060 |
| G | Deviation | Average (cv) | 6.85 |  | 1060 |
| H | Deviation | Standard (cv) | 9.19 |  | 1060 |
