# Supplementary material for: Calculation of the Isobaric Heat Capacities of the Liquid and Solid Phase of Organic Compounds at 298.15K by Means of the Group-Additivity Method
Source: Molecules. 2020 Mar 4;25(5):1147. doi: 10.3390/molecules25051147 (PMC7179130; doi:10.3390/molecules25051147)
Supplement: Supplementary file 1 [file molecules-25-01147-s001.zip › Table 4.doc]

| **Molecule name** | **C_p_(liq,298) calc. (GA)** | **Dev. (%)** | **C_p_(liq,298) exp.** | **Dev. (%)** | **C_p_(liq,298) calc. (V_m_)** |  |
| --- | --- | --- | --- | --- | --- | --- |
| 1-Propanol | 155.30 | -5.73 | 146.88 | -8.12 | 158.80 |  |
| 2-Propanol | 177.70 | -15.07 | 154.43 | -2.83 | 158.80 |  |
| 2-Methyl-1-propanol | 183.40 | -1.30 | 181.05 | -5.05 | 190.20 |  |
| 1-Butanol | 185.40 | -4.65 | 177.16 | -7.81 | 191.00 |  |
| Cyclopentanol | 204.20 | -10.14 | 185.40 | -9.76 | 203.50 |  |
| 2-Butanol | 207.70 | -5.61 | 196.67 | 3.34 | 190.10 |  |
| Isopentyl alcohol | 213.50 | -1.86 | 209.60 | -5.92 | 222.00 |  |
| 1-Pentanol | 215.40 | -3.49 | 208.14 | -7.19 | 223.10 |  |
| 2-Methyl-2-propanol | 226.50 | -3.61 | 218.60 | 12.76 | 190.70 |  |
| Cyclohexanol | 230.40 | -7.97 | 213.40 | -9.89 | 234.50 |  |
| 3-Methyl-2-butanol | 235.80 | 4.11 | 245.90 | 9.68 | 222.10 |  |
| Cyclohexanemethanol | 236.10 | 0.17 | 236.50 | -12.05 | 265.00 |  |
| 3,3-Dimethyl-1-butanol | 237.10 | -0.43 | 236.08 | -7.51 | 253.80 |  |
| 3-Pentanol | 237.80 | 0.79 | 239.70 | 6.76 | 223.50 |  |
| 2-Ethyl-1-butanol | 243.50 | 1.28 | 246.65 | -2.74 | 253.40 |  |
| 2-Methyl-1-pentanol | 243.50 | 1.97 | 248.40 | -2.62 | 254.90 |  |
| 1-Hexanol | 245.50 | -1.15 | 242.70 | -5.44 | 255.90 |  |
| Cycloheptanol | 256.50 | -2.51 | 250.22 | -6.03 | 265.30 |  |
| 2-Methyl-2-butanol | 256.60 | -3.76 | 247.30 | 10.31 | 221.80 |  |
| trans-2-Methylcyclohexanol | 258.50 | 1.70 | 262.98 | -1.30 | 266.40 |  |
| cis-2-Methylcyclohexanol | 258.50 | 3.89 | 268.95 | 1.51 | 264.90 |  |
| 4-Methyl-2-pentanol | 265.90 | 2.36 | 272.34 | 6.66 | 254.20 |  |
| 3-Methyl-2-pentanol | 265.90 | 3.62 | 275.89 | 7.93 | 254.00 |  |
| Cyclohexaneethanol | 266.20 | -0.08 | 266.00 | -12.03 | 298.00 |  |
| 2-Hexanol | 267.90 | -4.52 | 256.31 | 0.20 | 255.80 |  |
| 3-Hexanol | 267.90 | 0.51 | 269.27 | 5.49 | 254.50 |  |
| 1-Heptanol | 275.50 | -0.25 | 274.81 | -4.84 | 288.10 |  |
| 1-Methylcyclohexanol | 279.20 | -0.05 | 279.05 | 5.03 | 265.00 |  |
| 2-Methyl-2-pentanol | 286.70 | 0.81 | 289.03 | 11.84 | 254.80 |  |
| 3-Methyl-3-pentanol | 286.70 | 2.25 | 293.30 | 13.57 | 253.50 |  |
| 2,4-Dimethyl-3-pentanol | 294.00 | 5.77 | 312.00 | 8.53 | 285.40 |  |
| 5-Methyl-2-hexanol | 296.00 | -0.27 | 295.20 | 2.74 | 287.10 |  |
| Cyclohexanepropanol | 296.20 | -1.09 | 293.00 | -12.70 | 330.20 |  |
| 2-Heptanol | 297.90 | 0.24 | 298.63 | 3.59 | 287.90 |  |
| 3-Heptanol | 297.90 | 5.19 | 314.20 | 8.37 | 287.90 |  |
| 4-Heptanol | 297.90 | 2.89 | 306.77 | 6.15 | 287.90 |  |
| 2-Ethyl-1-hexanol | 303.60 | 4.38 | 317.50 | -0.28 | 318.40 |  |
| 2-Methyl-1-heptanol | 303.60 | 3.00 | 313.00 | -2.01 | 319.30 |  |
| 5-Methyl-1-heptanol | 303.60 | 0.20 | 304.20 | -4.73 | 318.60 |  |
| 1-Octanol | 305.60 | 2.08 | 312.10 | -2.40 | 319.60 |  |
| 2-Methyl-2-hexanol | 316.70 | -1.01 | 313.54 | 8.50 | 286.90 |  |
| 2,5-Dimethyl-3-hexanol | 324.00 | 4.54 | 339.40 | 6.25 | 318.20 |  |
| 2-Methyl-4-heptanol | 326.00 | 1.75 | 331.80 | 4.01 | 318.50 |  |
| 4-Methyl-2-heptanol | 326.00 | -4.32 | 312.50 | -1.70 | 317.80 |  |
| 4-Methyl-3-heptanol | 326.00 | -5.43 | 309.20 | -2.81 | 317.90 |  |
| 6-Methyl-2-heptanol | 326.00 | -3.46 | 315.10 | -1.30 | 319.20 |  |
| 6-Methyl-3-heptanol | 326.00 | -4.99 | 310.50 | -2.77 | 319.10 |  |
| 2-Octanol | 328.00 | 0.64 | 330.10 | 3.00 | 320.20 |  |
| 3-Octanol | 328.00 | 3.10 | 338.50 | 5.44 | 320.10 |  |
| 4-Octanol | 328.00 | 1.23 | 332.09 | 3.64 | 320.00 |  |
| 1-Nonanol | 335.70 | 1.55 | 341.00 | -3.37 | 352.50 |  |
| 2-Methyl-2-heptanol | 346.80 | -2.73 | 337.60 | 5.45 | 319.20 |  |
| 4-Methyl-4-heptanol | 346.80 | 5.48 | 366.90 | 13.25 | 318.30 |  |
| 2-Nonanol | 358.00 | -0.47 | 356.32 | 1.10 | 352.40 |  |
| 3-Nonanol | 358.00 | 4.18 | 373.63 | 5.71 | 352.30 |  |
| 4-Nonanol | 358.00 | 2.68 | 367.86 | 4.26 | 352.20 |  |
| 5-Nonanol | 358.00 | 3.44 | 370.75 | 4.98 | 352.30 |  |
| 3,7-Dimethyl-1-octanol | 361.80 | 1.47 | 367.21 | -4.05 | 382.10 |  |
| n-Decyl alcohol | 365.70 | 3.00 | 377.00 | -1.83 | 383.90 |  |
| 5-Decanol | 388.10 | 4.35 | 405.77 | 5.24 | 384.50 |  |
| 1-Undecanol | 395.80 | 2.59 | 406.34 | -2.60 | 416.90 |  |
| 1-Dodecanol | 425.80 | 2.88 | 438.42 | -2.44 | 449.10 |  |
| 1-Tridecanol | 455.90 | 4.22 | 476.00 | -1.11 | 481.30 |  |
| Myristyl alcohol | 486.00 | 3.91 | 505.80 | -1.52 | 513.50 |  |
| 1-Pentadecanol | 516.00 | 3.57 | 535.10 | -1.98 | 545.70 |  |
| 1-Hexadecanol | 546.10 | -4.26 | 523.80 | -10.33 | 577.90 |  |
| MAPD |  | 3.02 |  | 5.51 |  |  |
