# Supplementary material for: Calculation of the Isobaric Heat Capacities of the Liquid and Solid Phase of Organic Compounds at 298.15K by Means of the Group-Additivity Method
Source: Molecules. 2020 Mar 4;25(5):1147. doi: 10.3390/molecules25051147 (PMC7179130; doi:10.3390/molecules25051147)
Supplement: Supplementary file 1 [file molecules-25-01147-s001.zip › Table 5.doc]

| **Molecule name** | **C_p_(liq,298) calc. (GA)** | **Dev. (%)** | **C_p_(liq,298) exp.** | **Dev. (%)** | **C_p_(liq,298) calc.(V_m_)** |
| --- | --- | --- | --- | --- | --- |
| Cyclopentane | 130.70 | -1.48 | 128.80 | -15.30 | 148.50 |
| Pentane | 164.30 | 1.73 | 167.19 | -0.01 | 167.20 |
| Cyclohexane | 156.80 | 0.82 | 158.10 | -12.14 | 177.30 |
| Hexane | 194.30 | 1.70 | 197.66 | 0.08 | 197.50 |
| Cycloheptane | 183.00 | -1.32 | 180.61 | -13.23 | 204.50 |
| Heptane | 224.40 | 0.41 | 225.33 | -1.10 | 227.80 |
| Cyclooctane | 209.10 | 2.98 | 215.53 | -10.29 | 237.70 |
| Octane | 254.40 | 0.50 | 255.68 | -0.99 | 258.20 |
