# Supplementary material for: Calculation of the Isobaric Heat Capacities of the Liquid and Solid Phase of Organic Compounds at 298.15K by Means of the Group-Additivity Method
Source: Molecules. 2020 Mar 4;25(5):1147. doi: 10.3390/molecules25051147 (PMC7179130; doi:10.3390/molecules25051147)
Supplement: Supplementary file 1 [file molecules-25-01147-s001.zip › Table 6.doc]

| Molecule name | C_p_(liq,298) GA-calc. | Dev. (%) | C_p_(liq,298) exp. | Dev. (%) | C_p_(liq,298) V_m_-calc. |
| --- | --- | --- | --- | --- | --- |
| 1-Ethyl-3-methylimidazolium bromide | 256.40 | 3.17 | 264.80 | 3.29 | 256.10 |
| 1-Propyl-3-methylimidazolium bromide | 286.50 | -1.81 | 281.40 | -1.92 | 286.80 |
| 1-Ethyl-3-methylimidazolium thiocyanate | 300.00 | -6.59 | 281.45 | -4.96 | 295.40 |
| 1-Ethyl-3-methylimidazolium acetate | 321.10 | 0.25 | 321.90 | 7.86 | 296.60 |
| 1-Ethyl-3-methylimidazolium tetrafluoroborate | 307.60 | 0.16 | 308.10 | 1.95 | 302.10 |
| 1,3-Dimethylimidazolium methosulfate | 326.20 | 4.34 | 341.00 | 10.50 | 305.20 |
| 1-Butyl-3-methylimidazolium chloride | 316.50 | 0.16 | 317.00 | 3.63 | 305.50 |
| 1-Ethyl-3-methylimidazolium dicyanamide | 313.00 | 0.52 | 314.64 | -0.08 | 314.90 |
| 1-Butyl-3-methylimidazolium bromide | 316.50 | 0.06 | 316.70 | -0.38 | 317.90 |
| N-Methyl-2-hydroxyethylammonium propionate | 333.30 | -1.62 | 328.00 | 2.10 | 321.10 |
| 1-Ethyl-3-methylimidazolium methanesulfonate | 345.40 | 0.03 | 345.50 | 6.80 | 322.00 |
| 1-Ethyltetrahydrothiophenium dicyanamide | 339.60 | -1.26 | 335.38 | 1.34 | 330.90 |
| 1-Ethyl-3-methylimidazolium methylsulfate | 353.70 | -3.72 | 341.00 | 0.67 | 338.70 |
| 1-Ethyl-3-methylimidazolium hexafluorophosphate | 353.00 | -2.74 | 343.60 | 0.67 | 341.30 |
| 1-Butyl-3-methylimidazolium iodide | 316.50 | -0.80 | 314.00 | -8.82 | 341.70 |
| 1-Benzyl-3-methylimidazolium chloride | 341.00 | -0.47 | 339.40 | -1.30 | 343.80 |
| 1-Ethylpyridinium triflate | 348.70 | 0.88 | 351.80 | 0.71 | 349.30 |
| 1-Ethyl-3-methylimidazolium trifluoromethylsulfonate | 363.80 | -0.28 | 362.80 | 3.64 | 349.60 |
| N-Methyl-2-hydroxyethylammonium butanoate | 363.40 | -0.66 | 361.00 | 2.13 | 353.30 |
| 1-Butyl-3-methylimidazolium thiocyanate | 360.20 | 6.44 | 385.00 | 7.53 | 356.00 |
| 1-Butyl-3-methylimidazolium acetate | 381.20 | 0.52 | 383.20 | 6.16 | 359.60 |
| 1-Butyl-3-methylimidazolium tetrafluoroborate | 367.70 | -0.79 | 364.80 | 0.63 | 362.50 |
| 1-Ethyl-3-methylimidazolium ethosulfate | 383.30 | -1.40 | 378.00 | 2.99 | 366.70 |
| 1-Propyl-3-methylimidazolium hexafluorophosphate | 383.00 | -2.30 | 374.40 | 0.64 | 372.00 |
| 1-Butyl-3-methylimidazolium dicyanoamide | 373.10 | -2.22 | 365.00 | -2.19 | 373.00 |
| 1-Butyl-3-methylimidazolium trifluoroacetate | 411.10 | -0.71 | 408.20 | 5.66 | 385.10 |
| N-Methyl-2-hydroxyethylammonium pentanoate | 393.40 | 1.90 | 401.00 | 3.87 | 385.50 |
| 1-Butyltetrahydrothiophenium dicyanamide | 399.70 | -1.14 | 395.19 | 0.96 | 391.40 |
| 1-Butyl-3-methylpyridinium tetrafluoroborate | 379.20 | 2.27 | 388.00 | -1.26 | 392.90 |
| 1-Ethyl-3-methylpyridinium ethylsulfate | 394.70 | -1.47 | 389.00 | -2.08 | 397.10 |
| 1-Butyl-3-methylimidazolium methosulfate | 413.80 | 0.53 | 416.00 | 4.50 | 397.30 |
| 1-Benzyl-3-methylimidazolium tetrafluoroborate | 392.20 | -1.21 | 387.50 | -3.23 | 400.00 |
| 1-Butyl-3-methylimidazolium hexafluorophosphate | 413.10 | -1.32 | 407.70 | 1.37 | 402.10 |
| 1-Butyl-1-methylpyrrolidinium dicyanamide | 397.80 | 3.68 | 413.00 | 1.86 | 405.30 |
| 1-Butyl-3-methylimidazolium trifluoromethylsulfonate | 423.90 | -1.65 | 417.00 | 1.75 | 409.70 |
| 1-Hexyl-3-methylimidazolium tetrafluoroborate | 427.90 | -2.86 | 416.00 | -1.32 | 421.50 |
| 1-Pentyl-3-methylimidazolium hexafluorophosphate | 443.10 | -1.30 | 437.40 | 1.03 | 432.90 |
| 1-Butyl-1-methylpyrrolidinium trifluoromethanesulfonate | 448.70 | -3.15 | 435.00 | -0.55 | 437.40 |
| 1-Ethyl-3-methylimidazolium toluenesulfonate | 486.30 | -0.43 | 484.20 | 6.84 | 451.10 |
| 1-Hexyl-3-methylimidazolium trifluoromethylsulfonate | 484.00 | 3.64 | 502.30 | 6.43 | 470.00 |
| 1-Octyl-3-methylimidazolium tetrafluoroborate | 488.00 | 2.01 | 498.00 | 2.99 | 483.10 |
| 1-Ethyl-3-methylimidazolium bis(trifluoromethanesulfonyl) amide | 506.70 | -1.34 | 500.00 | 3.36 | 483.20 |
| N-Ethylpyridinium bis(trifluoromethylsulfonyl)amide | 491.50 | 2.12 | 502.15 | 2.98 | 487.20 |
| 1-Ethyl-3-methylimidazolium 2-(2-methoxyethoxy)ethylsulfate | 530.50 | -0.86 | 526.00 | 6.29 | 492.90 |
| 1-Heptyl-3-methylimidazolium hexafluorophosphate | 503.30 | -0.54 | 500.60 | 1.34 | 493.90 |
| 1-Isopropyl-3-methylimidazolium bis(trifluoromethanesulfonyl) amide | 535.20 | -1.00 | 529.90 | 3.25 | 512.70 |
| 1-Propyl-3-methylimidazolium bis(trifluoromethanesulfonyl) amide | 536.70 | -0.34 | 534.90 | 4.08 | 513.10 |
| 1-Butyl-3-methylimidazolium toluenesulfonate | 546.40 | 0.36 | 548.40 | 6.20 | 514.40 |
| N-Ethyl-2-methylpyridinium bis(trifluoromethylsulfonyl)amide | 535.30 | -0.15 | 534.50 | 3.55 | 515.50 |
| 1-Octyl-3-methylimidazolium hexafluorophosphate | 533.30 | 0.52 | 536.10 | 2.16 | 524.50 |
| 1-Cyclopropylmethyl-3-methylimidazolium bis(trifluoromethanesulfonyl) amide | 551.50 | -2.30 | 539.10 | 1.21 | 532.60 |
| Trimethyl butylammonium bis(trifluoromethylsulfonyl)amide | 561.10 | -0.34 | 559.20 | 4.02 | 536.70 |
| 1,2-Diethylpyridinium bis(trifluoromethanesulfonyl) amide | 565.40 | 0.12 | 566.10 | 4.82 | 538.80 |
| 1-Butyl-3-methylimidazolium bis(trifluoromethanesulfonyl) amide | 566.80 | -0.16 | 565.90 | 4.22 | 542.00 |
| 1-sec-Butyl-3-methylimidazolium bis(trifluoromethanesulfonyl) amide | 565.30 | -1.47 | 557.10 | 2.64 | 542.40 |
| 1-Methyl-1-propylpyrrolidinium bis(trifluoromethanesulfonyl) amide | 561.50 | -1.35 | 554.00 | 1.93 | 543.30 |
| 1-Isobutyl-3-methylimidazolium bis(trifluoromethanesulfonyl) amide | 564.80 | -1.38 | 557.10 | 2.48 | 543.30 |
| N-Propyl-2-methylpyridinium bis(trifluoromethylsulfonyl)amide | 565.40 | -1.33 | 557.96 | 2.18 | 545.80 |
| N-Butylpyridinium bis(trifluoromethanesulfonyl) amide | 551.60 | 2.63 | 566.52 | 3.50 | 546.70 |
| 1-Nonyl-3-methylimidazolium hexafluorophosphate | 563.40 | 1.05 | 569.40 | 2.79 | 553.50 |
| N-Octylisoquinolinium thiocyanate | 528.30 | -1.21 | 522.00 | -6.88 | 557.90 |
| 1-Butyltetrahydrothiophenium bis(trifluoromethylsulfonyl) amide | 593.40 | 0.44 | 596.00 | 6.12 | 559.50 |
| N-Ethyl-4-dimethylaminopyridinium bis(trifluoromethanesulfonyl) amide | 591.20 | 0.52 | 594.30 | 4.37 | 568.30 |
| 1-Pentyl-3-methylimidazolium bis(trifluoromethanesulfonyl) amide | 596.90 | -0.22 | 595.60 | 4.08 | 571.30 |
| 1-Ethyl-2-propylpyridinium bis(trifluoromethanesulfonyl) amide | 595.40 | -0.25 | 593.90 | 3.25 | 574.60 |
| 1-Isobutyl-1-methylpyrrolidinium bis(trifluoromethylsulfonyl)amide | 589.60 | -1.27 | 582.20 | 1.03 | 576.20 |
| 1-Isobutyl-3-methylpyridinium bis(trifluoromethylsulfonyl)amide | 576.30 | 0.47 | 579.00 | 0.40 | 576.70 |
| N-Butyl-3-methylpyridinium bis(trifluoromethylsulfonyl)amide | 578.20 | -0.02 | 578.10 | 0.14 | 577.30 |
| 1-Butyl-1-methylpyrrolidinium bis(trifluoromethylsulfonyl)amide | 591.50 | -3.41 | 572.00 | -1.05 | 578.00 |
| 1-Butyl-3-cyanopyridinium bis(trifluoromethylsulfonyl)amide | 590.00 | -0.68 | 586.00 | 1.11 | 579.50 |
| 1-Benzyl-3-methylimidazolium bis(trifluoromethylsulfonyl) amide | 591.20 | 2.73 | 607.80 | 4.34 | 581.40 |
| 1-Decyl-3-methylimidazolium hexafluorophosphate | 593.40 | 1.64 | 603.30 | 3.03 | 585.00 |
| 1-Pentyltetrahydrothiophenium bis(trifluoromethylsulfonyl) amide | 623.50 | 0.56 | 627.00 | 6.19 | 588.20 |
| 1-Hexyl-3-methylimidazolium bis(trifluoromethanesulfonyl) amide | 626.90 | 0.37 | 629.20 | 4.04 | 603.80 |
| 1-Butyl-1-methylpiperidinium bis(trifluoromethylsulfonyl)amide | 617.70 | -1.68 | 607.50 | 0.23 | 606.10 |
| 1-Ethyl-2-butylpyridinium bis(trifluoromethanesulfonyl) amide | 625.50 | -0.30 | 623.60 | 2.79 | 606.20 |
| N-Hexylpyridinium bis(trifluoromethanesulfonyl) amide | 611.80 | 0.03 | 612.00 | 0.83 | 606.90 |
| 1-Methyl-1-pentylpyrrolidinium bis(trifluoromethanesulfonyl) amide | 621.60 | 0.16 | 622.60 | 2.28 | 608.40 |
| 1-Butyl-3-methylimidazolium octylsulfate | 623.80 | 1.76 | 635.00 | 3.92 | 610.10 |
| 1-Cyclohexylmethyl-3-methylimidazolium bis(trifluoromethanesulfonyl) amide | 617.50 | -0.06 | 617.10 | 0.26 | 615.50 |
| 1-Hexyltetrahydrothiophenium bis(trifluoromethylsulfonyl) amide | 653.60 | -1.18 | 646.00 | 4.30 | 618.20 |
| N-Butyl-4-dimethylaminopyridinium bis(trifluoromethanesulfonyl) amide | 651.40 | 0.96 | 657.71 | 4.71 | 626.70 |
| 1-Heptyl-3-methylimidazolium bis(trifluoromethanesulfonyl) amide | 657.00 | 0.33 | 659.20 | 4.31 | 630.80 |
| 1-Ethyl-2-pentylpyridinium bis(trifluoromethanesulfonyl) amide | 655.60 | -0.44 | 652.70 | 2.47 | 636.60 |
| 1-Hexyl-3-methylpyridinium bis(trifluoromethylsulfonyl)amide | 638.30 | -2.29 | 624.00 | -2.20 | 637.70 |
| 1-Hexyl-1-methylpyrrolidinium bis(trifluoromethanesulfonyl) amide | 651.70 | 0.52 | 655.10 | 2.49 | 638.80 |
| 1-Hexyl-4-cyanopyridinium bis(trifluoromethylsulfonyl)amide | 650.10 | -2.70 | 633.00 | -1.04 | 639.60 |
| 1-Hexyl-3-cyanopyridinium bis(trifluoromethylsulfonyl)amide | 650.10 | 1.20 | 658.00 | 2.78 | 639.70 |
| 1-Dodecyl-3-methylimidazolium hexafluorophosphate | 653.60 | 1.91 | 666.30 | 2.97 | 646.50 |
| 1-Heptyltetrahydrothiophenium bis(trifluoromethylsulfonyl) amide | 683.60 | 0.20 | 685.00 | 5.37 | 648.20 |
| 1-Octyl-3-methylimidazolium bis(trifluoromethanesulfonyl) amide | 687.10 | 0.45 | 690.20 | 3.80 | 664.00 |
| 1-Methyl-1-heptylpyrrolidinium bis(trifluoromethanesulfonyl) amide | 681.70 | 0.50 | 685.10 | 2.96 | 664.80 |
| 1-Octylpyridinium bis(trifluoromethylsulfonyl)amide | 671.90 | 2.06 | 686.00 | 2.93 | 665.90 |
| 1-Ethyl-2-hexylpyridinium bis(trifluoromethanesulfonyl) amide | 685.60 | -0.01 | 685.50 | 2.68 | 667.10 |
| 1-Octyltetrahydrothiophenium bis(trifluoromethylsulfonyl) amide | 713.70 | 0.74 | 719.00 | 5.63 | 678.50 |
| 1-(3,4,5,6-Perfluorohexyl)-3-methylimidazolium-3-methylimidazolium bis(trifluoromethanesulfonyl) amide | 719.30 | 0.79 | 725.00 | 6.40 | 678.60 |
| 4-Dimethylamino-1-hexylpyridinium bis(trifluoromethanesulfonyl) amide | 711.50 | 2.67 | 731.00 | 5.99 | 687.20 |
| 1-Ethyl-2-heptylpyridinium bis(trifluoromethanesulfonyl) amide | 715.70 | 0.25 | 717.50 | 2.80 | 697.40 |
| 1-Methyl-1-octylpyrrolidinium bis(trifluoromethanesulfonyl) amide | 711.80 | 0.63 | 716.30 | 2.36 | 699.40 |
| 1-Octyl-3-cyanopyridinium bis(trifluoromethylsulfonyl)amide | 710.20 | -0.17 | 709.00 | 1.26 | 700.10 |
| N-Hexyl-3-methyl-4-dimethylaminopyridinium bis(trifluoromethanesulfonyl) amide | 738.10 | -1.81 | 725.00 | 1.94 | 710.90 |
| 1-Nonyltetrahydrothiophenium bis(trifluoromethylsulfonyl) amide | 743.70 | -0.36 | 741.00 | 4.01 | 711.30 |
| Butyl 1-butylnicotinate bis(trifluoromethylsulfonyl)amide | 728.90 | -3.10 | 707.00 | -0.91 | 713.40 |
| 1-Decyl-3-methylimidazolium bis(trifluoromethanesulfonyl) amide | 747.20 | 1.01 | 754.80 | 4.03 | 724.40 |
| 1-Ethyl-2-octylpyridinium bis(trifluoromethanesulfonyl) amide | 745.70 | 0.49 | 749.40 | 3.26 | 725.00 |
| 1-Methyl-3-menthyloxymethylimidazolium bis(trifluoromethylsulfonyl)imide | 785.90 | -0.58 | 781.40 | 4.20 | 748.60 |
| 1-Ethyl-2-nonylpyridinium bis(trifluoromethanesulfonyl) amide | 775.80 | 0.35 | 778.50 | 3.48 | 751.40 |
| 1-Methyl-1-decylpyrrolidinium bis(trifluoromethanesulfonyl) amide | 771.90 | 0.90 | 778.90 | 2.40 | 760.20 |
| 1-Ethyl-3-menthyloxymethylimidazolium bis(trifluoromethylsulfonyl)imide | 813.40 | 0.61 | 818.40 | 4.55 | 781.20 |
| 1-Dodecyl-3-methylimidazolium bis(trifluoromethanesulfonyl) amide | 807.30 | 1.57 | 820.20 | 4.04 | 787.10 |
| 1-Ethyl-2-decylpyridinium bis(trifluoromethanesulfonyl) amide | 805.80 | 0.67 | 811.20 | 2.79 | 788.60 |
| 1-Propyl-3-menthyloxymethylimidazolium bis(trifluoromethylsulfonyl)imide | 843.50 | -0.49 | 839.40 | 3.71 | 808.30 |
| 1-Butyl-3-menthyloxymethylimidazolium bis(trifluoromethylsulfonyl)imide | 873.50 | -0.84 | 866.20 | 3.20 | 838.50 |
| 1-Pentyl-3-menthyloxymethylimidazolium bis(trifluoromethylsulfonyl)imide | 903.60 | 0.15 | 905.00 | 3.55 | 872.90 |
| 1-Hexyl-3-menthyloxymethylimidazolium bis(trifluoromethylsulfonyl)imide | 933.70 | 1.15 | 944.60 | 4.74 | 899.80 |
| 1-Heptyl-3-menthyloxymethylimidazolium bis(trifluoromethylsulfonyl)imide | 963.70 | -0.33 | 960.50 | 3.25 | 929.30 |
| 1-Octyl-3-menthyloxymethylimidazolium bis(trifluoromethylsulfonyl)imide | 993.80 | 0.12 | 995.00 | 3.59 | 959.30 |
| 1-Nonyl-3-menthyloxymethylimidazolium bis(trifluoromethylsulfonyl)imide | 1023.80 | -0.02 | 1023.60 | 2.83 | 994.60 |
| 1-Decyl-3-menthyloxymethylimidazolium bis(trifluoromethylsulfonyl)imide | 1053.90 | -0.76 | 1045.90 | 2.31 | 1021.70 |
| 1-Undecyl-3-menthyloxymethylimidazolium bis(trifluoromethylsulfonyl)imide | 1084.00 | 0.40 | 1088.30 | 3.55 | 1049.70 |
| 1-Dodecyl-3-menthyloxymethylimidazolium bis(trifluoromethylsulfonyl)imide | 1114.00 | 0.35 | 1117.90 | 2.85 | 1086.00 |
| Tetradecyl trihexylphosphonium bis(trifluoromethylsulfonyl)amide | 1312.10 | -1.02 | 1298.80 | -1.35 | 1316.30 |
| MAPD |  | 1.13 |  | 3.08 |  |
