# Supplementary material for: Calculation of the Isobaric Heat Capacities of the Liquid and Solid Phase of Organic Compounds at 298.15K by Means of the Group-Additivity Method
Source: Molecules. 2020 Mar 4;25(5):1147. doi: 10.3390/molecules25051147 (PMC7179130; doi:10.3390/molecules25051147)
Supplement: Supplementary file 1 [file molecules-25-01147-s001.zip › Table 7.doc]

| **Entry** | **Atom Type** | **Neighbours** | **Contribution** | **Occurrences** | **Molecules** |
| --- | --- | --- | --- | --- | --- |
| 1 | B(-) | F4 | 2.99 | 1 | 1 |
| 2 | C sp3 | H3C | 37.12 | 569 | 246 |
| 3 | C sp3 | H3N | 101.28 | 50 | 34 |
| 4 | C sp3 | H3N(+) | 99.59 | 7 | 4 |
| 5 | C sp3 | H3O | 68.06 | 51 | 35 |
| 6 | C sp3 | H3S | 45.85 | 3 | 3 |
| 7 | C sp3 | H3P | 131.12 | 1 | 1 |
| 8 | C sp3 | H3Si | 59.18 | 14 | 5 |
| 9 | C sp3 | H2C2 | 25.45 | 1427 | 249 |
| 10 | C sp3 | H2CN | 82.72 | 89 | 56 |
| 11 | C sp3 | H2CN(+) | 81.35 | 19 | 15 |
| 12 | C sp3 | H2CO | 64.67 | 210 | 108 |
| 13 | C sp3 | H2CS | 72.27 | 22 | 12 |
| 14 | C sp3 | H2CF | 53.68 | 4 | 1 |
| 15 | C sp3 | H2CCl | 50.12 | 4 | 1 |
| 16 | C sp3 | H2CBr | 54.73 | 5 | 2 |
| 17 | C sp3 | H2CJ | 52.94 | 4 | 1 |
| 18 | C sp3 | H2CSi | 63.12 | 1 | 1 |
| 19 | C sp3 | H2N2 | 134.39 | 13 | 3 |
| 20 | C sp3 | H2O2 | 108.47 | 12 | 3 |
| 21 | C sp3 | H2S2 | -6.67 | 3 | 3 |
| 22 | C sp3 | HC3 | 11.92 | 164 | 73 |
| 23 | C sp3 | HC2N | 72.94 | 28 | 23 |
| 24 | C sp3 | HC2N(+) | 70.74 | 29 | 28 |
| 25 | C sp3 | HC2O | 51.84 | 161 | 63 |
| 26 | C sp3 | HC2S | 47.22 | 4 | 2 |
| 27 | C sp3 | HC2Si | 142.27 | 1 | 1 |
| 28 | C sp3 | HCN2 | 137.04 | 1 | 1 |
| 29 | C sp3 | HCNO | 119.89 | 7 | 5 |
| 30 | C sp3 | HCNS | 116.08 | 2 | 1 |
| 31 | C sp3 | HCO2 | 112.62 | 17 | 14 |
| 32 | C sp3 | HCF2 | 246.8 | 1 | 1 |
| 33 | C sp3 | HCBr2 | 68.67 | 1 | 1 |
| 34 | C sp3 | C4 | -2.17 | 81 | 48 |
| 35 | C sp3 | C3N | 62.09 | 11 | 9 |
| 36 | C sp3 | C3N(+) | 19.23 | 2 | 2 |
| 37 | C sp3 | C3O | 27.59 | 10 | 10 |
| 38 | C sp3 | C3Cl | 78.13 | 1 | 1 |
| 39 | C sp3 | C3Br | 44.34 | 1 | 1 |
| 40 | C sp3 | C2NO | 89.86 | 1 | 1 |
| 41 | C sp3 | C2O2 | 91.75 | 6 | 5 |
| 42 | C sp3 | C2S2 | 43.86 | 5 | 2 |
| 43 | C sp3 | CF3 | 69.89 | 2 | 2 |
| 44 | C sp3 | CSF2 | 0 | 1 | 1 |
| 45 | C sp3 | CCl3 | 92.28 | 4 | 3 |
| 46 | C sp2 | H2=C | 39.96 | 6 | 6 |
| 47 | C sp2 | HC=C | 16.95 | 109 | 65 |
| 48 | C sp2 | HC=N | 99.99 | 13 | 13 |
| 49 | C sp2 | HC=O | 43.05 | 14 | 12 |
| 50 | C sp2 | H=CN | 33.94 | 25 | 17 |
| 51 | C sp2 | H=CO | 41.99 | 3 | 3 |
| 52 | C sp2 | H=CS | 36.19 | 7 | 5 |
| 53 | C sp2 | H=CCl | 19.44 | 1 | 1 |
| 54 | C sp2 | HN=N | 102.56 | 17 | 14 |
| 55 | C sp2 | HN=O | 28.91 | 4 | 3 |
| 56 | C sp2 | H=NO | 112.57 | 1 | 1 |
| 57 | C sp2 | C2=C | 5.77 | 29 | 22 |
| 58 | C sp2 | C2=N | 95.44 | 14 | 10 |
| 59 | C sp2 | C2=N(+) | -9.61 | 2 | 2 |
| 60 | C sp2 | C=CN | 18.4 | 16 | 15 |
| 61 | C sp2 | C2=O | 27.68 | 44 | 30 |
| 62 | C sp2 | C=CO | 25.28 | 15 | 13 |
| 63 | C sp2 | C=CS | 29.08 | 5 | 4 |
| 64 | C sp2 | C=CCl | 35.2 | 6 | 3 |
| 65 | C sp2 | =CN2 | 38.93 | 14 | 14 |
| 66 | C sp2 | =CN2(+) | 78.32 | 7 | 7 |
| 67 | C sp2 | CN=N | 87.15 | 19 | 13 |
| 68 | C sp2 | CN=N(+) | 118.23 | 2 | 1 |
| 69 | C sp2 | CN=O | 37.33 | 131 | 92 |
| 70 | C sp2 | =CNO | 55.09 | 1 | 1 |
| 71 | C sp2 | CN=S | 46.51 | 3 | 3 |
| 72 | C sp2 | CO=O | 51.25 | 208 | 155 |
| 73 | C sp2 | CO=O(-) | 15.25 | 41 | 40 |
| 74 | C sp2 | C=OCl | 61.46 | 2 | 1 |
| 75 | C sp2 | =CS2 | 45.53 | 12 | 2 |
| 76 | C sp2 | N2=N | 113.97 | 5 | 3 |
| 77 | C sp2 | N2=O | 55.72 | 43 | 38 |
| 78 | C sp2 | N=NO | 91.56 | 1 | 1 |
| 79 | C sp2 | N2=S | 67.02 | 7 | 7 |
| 80 | C sp2 | N=NS | 105.95 | 7 | 7 |
| 81 | C sp2 | NO=O | 63.43 | 8 | 8 |
| 82 | C sp2 | NO=S | 64.85 | 3 | 3 |
| 83 | C sp2 | =NOS | 108.01 | 1 | 1 |
| 84 | C sp2 | NS=S | 62.46 | 4 | 3 |
| 85 | C sp2 | O2=O | 58.47 | 5 | 5 |
| 86 | C sp2 | OS=S | 63.23 | 1 | 1 |
| 87 | C aromatic | H:C2 | 17.96 | 3232 | 437 |
| 88 | C aromatic | H:C:N | 24.16 | 37 | 20 |
| 89 | C aromatic | H:C:N(+) | 21.47 | 2 | 1 |
| 90 | C aromatic | H:N2 | 7.08 | 3 | 3 |
| 91 | C aromatic | :C3 | 8.04 | 171 | 57 |
| 92 | C aromatic | C:C2 | 6.56 | 699 | 307 |
| 93 | C aromatic | C:C:N | 5.85 | 13 | 9 |
| 94 | C aromatic | :C2N | 24.29 | 172 | 107 |
| 95 | C aromatic | :C2N(+) | 49.48 | 57 | 42 |
| 96 | C aromatic | :C2:N | 19.63 | 13 | 7 |
| 97 | C aromatic | :C2O | 29.21 | 184 | 113 |
| 98 | C aromatic | :C2P | 15.1 | 6 | 2 |
| 99 | C aromatic | :C2S | 27.43 | 43 | 28 |
| 100 | C aromatic | :C2Si | 58.51 | 53 | 12 |
| 101 | C aromatic | :C2F | 31.08 | 25 | 9 |
| 102 | C aromatic | :C2Cl | 34.35 | 57 | 25 |
| 103 | C aromatic | :C2Br | 37.48 | 18 | 9 |
| 104 | C aromatic | :C2J | 48.49 | 5 | 3 |
| 105 | C aromatic | C:N2 | 18.81 | 3 | 1 |
| 106 | C aromatic | :CN:N | 39.03 | 7 | 5 |
| 107 | C aromatic | :C:NO | 53.92 | 1 | 1 |
| 108 | C aromatic | :C:NCl | 49.04 | 3 | 3 |
| 109 | C aromatic | N:N2 | 36.22 | 4 | 2 |
| 110 | C aromatic | :N2O | 40.81 | 3 | 1 |
| 111 | C(+) aromatic | H:N2 | -39.63 | 3 | 3 |
| 112 | C(+) aromatic | :N3 | -20.94 | 2 | 2 |
| 113 | C sp | H#C | 103.03 | 2 | 1 |
| 114 | C sp | C#C | 14.95 | 8 | 3 |
| 115 | C sp | C#N | 39.64 | 28 | 20 |
| 116 | C sp | C#N(+) | 62.56 | 1 | 1 |
| 117 | C sp | #CSi | 0 | 2 | 1 |
| 118 | C sp | #NO | 58.07 | 2 | 1 |
| 119 | C sp | =N=O | 110.98 | 6 | 3 |
| 120 | N sp3 | H2C | -18.62 | 5 | 5 |
| 121 | N sp3 | H2C(pi) | 15.49 | 129 | 98 |
| 122 | N sp3 | H2N | 9.51 | 4 | 3 |
| 123 | N sp3 | H2S | 45.92 | 9 | 9 |
| 124 | N sp3 | HC2 | -104.07 | 6 | 3 |
| 125 | N sp3 | HC2(pi) | -56.77 | 78 | 57 |
| 126 | N sp3 | HC2(2pi) | -9.78 | 82 | 61 |
| 127 | N sp3 | HCN(pi) | 21.22 | 7 | 5 |
| 128 | N sp3 | HCN(2pi) | 36.09 | 7 | 7 |
| 129 | N sp3 | C3 | -159.23 | 15 | 10 |
| 130 | N sp3 | C3(pi) | -123.38 | 10 | 9 |
| 131 | N sp3 | C3(2pi) | -83.83 | 27 | 21 |
| 132 | N sp3 | C3(3pi) | -43.15 | 12 | 6 |
| 133 | N sp3 | C2N(pi) | -75.07 | 3 | 3 |
| 134 | N sp3 | C2N(+)(pi) | -52.69 | 7 | 2 |
| 135 | N sp3 | C2N(2pi) | 25.39 | 3 | 3 |
| 136 | N sp3 | C2N(+)(2pi) | -41.18 | 2 | 2 |
| 137 | N sp2 | C=C | -74.59 | 54 | 44 |
| 138 | N sp2 | C=N | 2.46 | 5 | 3 |
| 139 | N sp2 | =CN | -98.01 | 7 | 7 |
| 140 | N sp2 | =CN(+) | -14.76 | 1 | 1 |
| 141 | N sp2 | =CO | -44.63 | 24 | 11 |
| 142 | N sp2 | N=N | 17.43 | 3 | 2 |
| 143 | N aromatic | H2:C(+) | 4.65 | 4 | 2 |
| 144 | N aromatic | HC:C(+) | 40.44 | 1 | 1 |
| 145 | N aromatic | C2:C(+) | -3.11 | 7 | 4 |
| 146 | N aromatic | :C2 | -0.16 | 50 | 33 |
| 147 | N(+) sp3 | H3C | -6.7 | 35 | 34 |
| 148 | N(+) sp3 | H2C2 | -73.79 | 4 | 4 |
| 149 | N(+) sp3 | HC3 | -156.71 | 1 | 1 |
| 150 | N(+) sp3 | C4 | -233.93 | 2 | 2 |
| 151 | N(+) sp2 | CO=O(-) | 9.71 | 72 | 49 |
| 152 | N(+) sp2 | =CO2(-) | -7.42 | 2 | 2 |
| 153 | N(+) sp2 | NO=O(-) | -2.08 | 10 | 5 |
| 154 | N(+) aromatic | C:C2 | 0 | 1 | 1 |
| 155 | N(+) sp | C#C(-) | 25.34 | 3 | 3 |
| 156 | N(+) sp | #CO(-) | 0 | 1 | 1 |
| 157 | O(prim) | HC | -23.36 | 106 | 60 |
| 158 | O(sec) | HC | -16.25 | 117 | 50 |
| 159 | O(tert) | HC | -3.34 | 6 | 6 |
| 160 | O | HC(pi) | 4.78 | 218 | 157 |
| 161 | O | HN(pi) | 16.33 | 3 | 3 |
| 162 | O | HSi | 16.93 | 8 | 2 |
| 163 | O | C2 | -70.39 | 60 | 29 |
| 164 | O | C2(pi) | -39.71 | 125 | 88 |
| 165 | O | C2(2pi) | -23.91 | 47 | 41 |
| 166 | O | CN(2pi) | -28.51 | 5 | 5 |
| 167 | O | CSi | -30.4 | 36 | 9 |
| 168 | O | N2(2pi) | 1.1 | 7 | 4 |
| 169 | O | N2(+)(2pi) | -2.2 | 2 | 2 |
| 170 | O | Si2 | -7.14 | 39 | 8 |
| 171 | P3 | C3 | -2.25 | 1 | 1 |
| 172 | P4 | C3=O | 2.25 | 1 | 1 |
| 173 | P4 | C=OCl2 | 0 | 1 | 1 |
| 174 | S2 | HC | 10.73 | 1 | 1 |
| 175 | S2 | HC(pi) | 16.07 | 5 | 5 |
| 176 | S2 | C2 | -15.43 | 12 | 8 |
| 177 | S2 | C2(pi) | -30.85 | 14 | 6 |
| 178 | S2 | C2(2pi) | -10.04 | 24 | 17 |
| 179 | S2 | CS | -20.66 | 4 | 2 |
| 180 | S2 | CS(pi) | 6.91 | 6 | 3 |
| 181 | S4 | C2=O | 5.33 | 2 | 2 |
| 182 | S4 | C2=O2 | 15.56 | 5 | 5 |
| 183 | S4 | CN=O2 | 2.75 | 9 | 9 |
| 184 | S4 | CO=O2(-) | -118.41 | 1 | 1 |
| 185 | Si | C4 | -197.51 | 3 | 3 |
| 186 | Si | C3O | -100.79 | 4 | 2 |
| 187 | Si | C3Cl | -107.38 | 1 | 1 |
| 188 | Si | C2O2 | -38.26 | 11 | 3 |
| 189 | Si | CO3 | 10.05 | 20 | 3 |
| 190 | Si | CCl3 | 57.89 | 2 | 2 |
| 191 | Si | O4 | 0 | 9 | 9 |
| 192 | (COH)n | n>1 | 3.46 | 145 | 59 |
| 193 | H | H Acceptor | 1.19 | 61 | 43 |
| 194 | Endocyclic bonds | No of single bds | -1.44 | 998 | 149 |
| 195 | Angle60 |  | 0.97 | 15 | 2 |
| 196 | Angle90 |  | 0.31 | 12 | 6 |
| 197 | Angle102 |  | 2.16 | 284 | 83 |
| A | Based on | Valid groups | 126 |  | 802 |
| B | Goodness of fit | R^2^ | 0.9915 |  | 734 |
| C | Deviation | Average | 9.36 |  | 734 |
| D | Deviation | Standard | 12.21 |  | 734 |
| E | K-fold cv | K | 10 |  | 663 |
| F | Goodness of fit | Q^2^ | 0.9874 |  | 663 |
| G | Deviation | Average (cv) | 11.1 |  | 663 |
| H | Deviation | Standard (cv) | 14.23 |  | 663 |
