# Supplementary material for: Calculation of the Isobaric Heat Capacities of the Liquid and Solid Phase of Organic Compounds at 298.15K by Means of the Group-Additivity Method
Source: Molecules. 2020 Mar 4;25(5):1147. doi: 10.3390/molecules25051147 (PMC7179130; doi:10.3390/molecules25051147)
Supplement: Supplementary file 1 [file molecules-25-01147-s001.zip › Table 8.doc]

| **Molecule name** | **C_p_(sol,298) GA-calc** | **Dev. (%)** | **C_p_(sol,298) exp.** | **Dev. (%)** | **C_p_(sol,298) V_m_-calc.[1]** |
| --- | --- | --- | --- | --- | --- |
| 2-Methyl-2-propanol | 135.90 | 6.99 | 146.11 | 18.28 | 119.40 |
| 2,2-Dimethyl-1,3-propanediol | 158.00 | 13.75 | 183.18 | 13.42 | 158.60 |
| Erythritol | 164.20 | -1.42 | 161.90 | 0.99 | 160.30 |
| cis-1,2-Cyclohexanediol | 168.00 | -4.74 | 160.40 | -4.68 | 167.90 |
| trans-1,2-Cyclohexanediol | 168.00 | -2.94 | 163.20 | -3.00 | 168.10 |
| Pentaerythritol | 174.40 | 7.43 | 188.40 | 3.24 | 182.30 |
| Hexamethyleneglycol | 187.90 | 1.11 | 190.00 | 2.47 | 185.30 |
| Xylitol | 206.90 | 0.05 | 207.00 | 10.14 | 186.00 |
| Ethriol | 191.10 | 10.62 | 213.80 | 7.86 | 197.00 |
| Inositol | 223.60 | -2.57 | 218.00 | 0.50 | 216.90 |
| 2-Adamantanol | 193.30 | 6.71 | 207.20 | -6.13 | 219.90 |
| 1-Adamantanol | 195.60 | 0.56 | 196.70 | -12.10 | 220.50 |
| Dulcose | 246.00 | -3.14 | 238.50 | 3.31 | 230.60 |
| Isoborneol | 243.10 | 6.88 | 261.06 | 10.21 | 234.40 |
| Borneol | 243.10 | 6.88 | 261.06 | 9.68 | 235.80 |
| 1,8-Octanediol | 238.90 | -1.07 | 236.36 | -0.23 | 236.90 |
| Sorbitol | 242.30 | -1.38 | 239.00 | 0.50 | 237.80 |
| Menthol | 250.70 | -0.24 | 250.10 | -0.72 | 251.90 |
| 1,9-Nonanediol | 264.30 | -2.94 | 256.74 | -2.36 | 262.80 |
| 1,10-Decanediol | 289.80 | -3.77 | 279.26 | -3.34 | 288.60 |
| 1,11-Undecanediol | 315.20 | -5.85 | 297.79 | -5.58 | 314.40 |
| Tri-t-butylmethanol | 351.80 | -0.34 | 350.60 | 6.36 | 328.30 |
| 1,12-Dodecanediol | 340.70 | -3.17 | 330.23 | -3.05 | 340.30 |
| 1-Tridecanol | 358.60 | 5.13 | 378.00 | 8.41 | 346.20 |
| 1,13-Tridecanediol | 366.20 | 0.19 | 366.88 | 0.21 | 366.10 |
| Myristyl alcohol | 384.00 | 1.03 | 388.00 | 4.28 | 371.40 |
| 1,14-Tetradecanediol | 391.60 | -3.16 | 379.61 | -3.24 | 391.90 |
| 1-Pentadecanol | 409.50 | -2.38 | 400.00 | 0.88 | 396.50 |
| 1,15-Pentadecanediol | 417.10 | -10.50 | 377.45 | -10.66 | 417.70 |
| 1-Hexadecanol | 435.00 | -3.08 | 422.00 | 0.09 | 421.60 |
| 1,16-Hexadecanediol | 442.50 | -3.83 | 426.18 | -4.06 | 443.50 |
| MAPD |  | 4.00 |  | 5.16 |  |
