# Supplementary material for: Calculation of the Isobaric Heat Capacities of the Liquid and Solid Phase of Organic Compounds at 298.15K by Means of the Group-Additivity Method
Source: Molecules. 2020 Mar 4;25(5):1147. doi: 10.3390/molecules25051147 (PMC7179130; doi:10.3390/molecules25051147)
Supplement: Supplementary file 1 [file molecules-25-01147-s001.zip › Table 9.doc]

| **Molecule name** | **C_p_(sol,298) GA-calc.** | **Dev. (%)** | **C_p_(sol,298) exp.** | **Dev. (%)** | **C_p_(sol,298) V_m_-calc.[1]** |
| --- | --- | --- | --- | --- | --- |
| Nortricyclene | 130.70 | -1.32 | 129.00 | -9.15 | 140.80 |
| Norbornane | 163.80 | -8.48 | 151.00 | 0.93 | 149.60 |
| Bicyclo[2.2.2]octane | 163.80 | -3.87 | 157.69 | -8.44 | 171.00 |
| Adamantane | 183.30 | 3.53 | 190.00 | -5.95 | 201.30 |
| Bicyclo[3.3.3]undecane | 236.10 | -10.74 | 213.20 | -9.19 | 232.80 |
| Diamantane | 222.10 | 0.58 | 223.40 | -17.32 | 262.10 |
| Perhydrophenanthrene | 279.60 | 3.42 | 289.50 | -0.73 | 291.60 |
| Tri-t-butylmethane | 339.50 | 4.31 | 354.80 | 15.30 | 300.50 |
| Cetane | 431.00 | 2.44 | 441.80 | 13.26 | 383.20 |
| Octadecane | 481.90 | 0.77 | 485.64 | 11.70 | 428.80 |
| Docosane | 583.80 | -3.58 | 563.60 | 7.59 | 520.80 |
| 2,11-Dicyclohexyldodecane | 563.40 | -1.09 | 557.30 | 4.65 | 531.40 |
| 1,1-Dicyclohexyldodecane | 565.20 | -0.46 | 562.60 | 5.44 | 532.00 |
| Hexacosane | 685.60 | -3.69 | 661.20 | 7.43 | 612.10 |
| Triacontane | 787.40 | 2.65 | 808.80 | 13.03 | 703.40 |
| Dotriacontane | 838.40 | -4.02 | 806.00 | 7.07 | 749.00 |
| Tetratriacontane | 889.30 | -0.21 | 887.40 | 10.45 | 794.70 |
| Pentatriacontane | 914.70 | 0.13 | 915.90 | 10.74 | 817.50 |
| MAPD |  | 3.07 |  | 8.80 |  |
